# Long-Read Evidence for Repeat-Associated Structural Heterogeneity in the Mitochondrial Genome of *Rhododendron spinuliferum*

**DOI:** 10.3390/genes17101288

**Published:** 2026-10-15

**Authors:** Jie Chen, Yikai Feng, Xinyi Qian, Deju Zhang

**Affiliations:** 1 Industrial College of Traditional Chinese Medicine and Health, Lishui University, Lishui 323000, China; 2 College of Agriculture and Biotechnology, Lishui University, Lishui 323000, China; lsufyikai@126.com (Y.F.); lsxyqianxinyi@126.com (X.Q.); 3 School of Biological Sciences, The University of Hong Kong, Hong Kong, China; u3005757@connect.hku.hk

**Keywords:** *Rhododendron spinuliferum*, mitochondrial genome, PacBio HiFi, repeat-mediated recombination, RNA editing

## Abstract

**Background/Objectives:** Plant mitochondrial genomes are structurally dynamic, and assembled paths may not reflect all molecular configurations. We characterized the mitochondrial genome of *Rhododendron spinuliferum* and evaluated repeat-associated structural heterogeneity and transcript support for mitochondrial RNA processing. **Methods:** Public PacBio HiFi reads were assembled and curated; Illumina reads from a second individual provided cross-individual sequence support. Candidate local connections were assessed by strict single-read two-junction phasing. Gene content, repeats, plastid-derived sequences, comparative genomics, phylogeny, predicted C-to-U RNA-editing sites, and mature exon junctions were also examined. **Results:** Three representative assembly paths totaled 786,593 bp and contained 32 protein-coding loci, three rRNA genes, and 19 tRNA types. A total of 226 HiFi reads supported four local configurations associated with two repeat pairs, and all four persisted across 24 filtering combinations. Nine mitochondrial plastid DNA transfer blocks covered 6.77 kb. Public RNA-seq data provided read-level support for 211 of 475 predicted editing sites and all 12 examined mature exon junctions of *nad1*, *nad2*, and *nad5*. Across six congeners, 99.7% of resolved homologous positions corresponding to RNA-supported sites retained genomic C. **Conclusions:** These results support repeat-associated local structural heterogeneity in the *R. spinuliferum* mitogenome and provide a curated DNA- and RNA-supported resource for comparative *Rhododendron* mitochondrial genomics.

## 1. Introduction

*Rhododendron spinuliferum* is used in Yi ethnic medicine in southwestern China [1]. Its ethnomedicinal importance has stimulated chemical and pharmacological studies, including the identification of a chromone glycoside and the phytocannabinoid-like meroterpenoids spinulinoids A–F [1,2,3,4]. More recently, a long-read plastome assembly provided a structurally validated chloroplast resource for this species [5]. By comparison, mitochondrial genomic information for *R. spinuliferum* remains scarce, and its mitochondrial organization and RNA-processing features are poorly characterized.

Plant mitochondrial genomes retain a relatively conserved set of core genes while showing remarkable variation in genome size, sequence order, repeat content, and noncoding regions [6]. Recombination across repeated sequences and intracellular DNA transfer are major contributors to this structural plasticity [6,7]. Consequently, plant mitochondrial DNA may exist as a dynamic population of linear, circular, branched, and multipartite molecules rather than as a single stable master circle [6,7]. High-accuracy long reads and graph-based assembly approaches are therefore especially valuable for resolving repeated regions and testing alternative sequence connections [7,8]. However, an assembled path remains a sequence representation unless its connections are supported directly by spanning reads or other molecular evidence.

Repeat-associated alternative mitochondrial configurations have been demonstrated in several angiosperms, including *Gelsemium elegans*, *Phellodendron amurense*, and *Rhodomyrtus tomentosa* [9,10,11]. Within *Rhododendron*, mitochondrial resources are available for *R. simsii* and *R. delavayi* [12,13], and long-read analyses of *R. nivale* subsp. *boreale* and *R. vialii* have revealed structural complexity, repeat-associated alternative conformations, and differences between mitochondrial and plastid phylogenies [14]. These studies establish substantial mitochondrial diversity within the genus, but they do not resolve how repeat-associated local configurations are represented in *R. spinuliferum* or whether its mitochondrial gene models can be independently supported at the transcript level.

Here, we assembled and manually curated the *R. spinuliferum* mitochondrial genome from public PacBio HiFi reads and evaluated alternative repeat-associated connections using strict single-read two-junction phasing. We further characterized gene content, dispersed repeats, simple sequence repeats, and mitochondrial plastid DNA transfer, and compared sequence conservation, synteny, and phylogenetic relationships with available congeneric mitogenomes. Public RNA-seq data were used as an independent layer of evidence for predicted C-to-U editing sites and mature exon junctions of split genes.

We addressed three questions: which local mitochondrial configurations are directly supported by individual long reads; how the gene content and sequence organization of *R. spinuliferum* compare with those of other sampled *Rhododendron* species; and to what extent transcript data support predicted RNA processing. This approach distinguishes representative assembly paths from read-supported local connections and integrates DNA- and RNA-level evidence for comparative studies of *Rhododendron* organelle genomes.

## 2. Materials and Methods

### 2.1. Sampling and Sequencing

Fresh leaves of *R. spinuliferum* were collected from Zixi Mountain National Forest Park, Chuxiong, Yunnan, China (25.05° N, 101.43° E), on 15 March 2026. Voucher LSU_20260312 was deposited at the Herbarium of Lishui University, China. DNA was extracted using the Rapid Plant Genomic DNA Isolation Kit (Sangon Biotech, Shanghai, China). Illumina sequencing (Illumina, Inc., San Diego, CA, USA) yielded 11,213,581 read pairs, with 151 bp per read (PE400 library). The Illumina dataset was generated from the voucher individual and used only for independent cross-individual mapping support; it was not used for assembly polishing. De novo mitochondrial assembly used public PacBio Sequel II HiFi reads from a different *R. spinuliferum* individual (CRR1094297), retrieved from the Genome Sequence Archive at the National Genomics Data Center.

### 2.2. Mitochondrial Genome Assembly and Path Resolution

Read group RG3_21Z00144B (1,583,100 HiFi reads) was assembled with Oatk v1.0 [8] using -k 1001 -s 31 -c 200 and the OatkDB v20230921 embryophyte mitochondrial and plastid HMM databases. The coverage filter (-c 200) was intended to reduce nuclear-derived sequences, including potential NUMTs, while organelle-specific HMMs guided sequence classification and pathfinder selected representative graph traversals. To estimate the nonredundant sequence span, cross-path homology was collapsed in the order ctg1 > ctg3 > ctg2 with MUMmer v3.23 (NUCmer v3.1, --maxmatch) [15]. Alignments were retained at lengths ≥ 500 bp and identities ≥ 80%. Circular paths were extended by 100 kb across their origins before alignment to minimize edge effects.

### 2.3. Read-Mapping Support and Junction-Phasing Analysis

HiFi and PE400 reads were mapped separately to each path with minimap2 v2.31-r1302 (map-hifi) [16] and BWA-MEM v0.7.18-r1243-dirty [17], respectively. SAMtools v1.21 [18] summarized primary-alignment depth per base and in 5-kb windows, both with and without MAPQ ≥ 20 filtering; overlapping mates were counted once. Circular paths were midpoint-rotated before mapping and then restored to their original coordinates. Because the PE400 reads came from a different individual, their alignments were used only as cross-individual sequence support. For local phasing, HiFi reads from the assembly individual were mapped competitively against four references representing alternative configurations around u15. Secondary and supplementary alignments were excluded. Retained reads had to span both diagnostic junctions with ≥1000 bp on each side, MAPQ ≥ 30, identity ≥ 99.5%, and a minimum alignment span of 11 kb. The 11-kb cutoff was a preliminary length filter for the 9796-bp u15 region flanked by the 1144- and 1020-bp repeat pairs; the separate requirement for ≥1000 bp of outer flanking coverage at both junctions corresponded to approximately 11.8 kb of reference sequence. Sensitivity analyses combined minimum spans of 11, 12, and 12.5 kb; MAPQ thresholds of 20 and 30; and identity thresholds of 98%, 99%, 99.5%, and 99.9%. Phase fractions were summarized with Wilson 95% confidence intervals. For an additional mapping-bias assessment, 6553 previously identified u15 candidate reads were independently mapped to each of the four local references. Retained reads were examined for continuous two-junction spanning and flanking 31-mers unique among the seven mitochondrial GFA segments. Best-to-second-best alignment-score differences (ΔAS) and empirical-profile PBSIM3 simulations were used to assess configuration-dependent classification and retention. For cross-individual comparisons, SNPs and small indels were screened from competitive three-path mappings using mapping/base qualities ≥ 30, ≥20 observations in each dataset, PE400 alternate-allele fraction ≥ 0.90, HiFi reference-allele fraction ≥ 0.95, strand support, and read-level checks. Repetitive or nonunique regions, MTPTs, and branch/path-end contexts were excluded; coverage gaps were assessed before and after quality filtering.

### 2.4. Genome Annotation and tRNA Validation

The three paths were annotated with PMGA [19], and tRNA predictions were checked with tRNAscan-SE v2.0.9 in organellar mode [20]. Gene models were manually curated against congeneric mitochondrial references using three BLAST+ programs (BLASTP, BLASTN, and TBLASTN; all v2.12.0+) [21]. Protein-coding loci were counted once regardless of duplicated copies or exons distributed across different paths; partial and pseudogene-like fragments were reported separately.

### 2.5. Repeat and Recombination Analysis

Forward and palindromic interspersed repeats were identified with Vmatch v2.3.1 [22] using a minimum repeat length of 30 bp and a maximum of three mismatches. Reverse-complement matches were classified as palindromic (inverted) repeats. The best 50 Vmatch records (-best 50) were retained; records with identical or overlapping graph-coordinate relationships were grouped into 30 operational groups for downstream analysis, and supporting reads were deduplicated. MISA [23] was used to identify SSRs with minimum repeat counts of 10, 6, 5, 5, 5, and 5 for mono- through hexanucleotide motifs, respectively. SSRs separated by ≤100 bp were grouped as compound loci. Repeat-associated alternative connections around u15 were evaluated using the junction-phasing procedure described in Section 2.3. To examine alternative connections beyond the focal u15 region, the retained Vmatch records were assessed for repeat-flanking support using single HiFi-read spans across each repeat and ≥500 bp of both flanks (MAPQ ≥ 20, identity ≥ 99%, ΔAS ≥ 50), graph-unique 31-mers, and independent BLASTN checks. Reliable support required ≥3 qualifying reads under the main filter and ≥2 under stricter filtering (Table S2).

### 2.6. Mitochondrial Plastid DNA Transfer

The published *R. spinuliferum* plastome PZ603433 [5] was aligned to the mitochondrial sequences with BLASTN v2.12.0+ [21] using a minimum length of 100 bp, a minimum identity of 80%, and an E-value ≤ 1 × 10^−10^. Overlapping hits were merged into mitochondrial plastid DNA transfer (MTPT) blocks and assigned to plastome regions and annotations. Enrichment of IR-derived sequence was tested with 1,000,000 interval-preserving permutations under three null models: full-plastome, single-IR-representation, and independent-event models. The full-plastome model randomized the lengths of 18 plastome source intervals on the 209,482-bp circular plastome with both IR copies retained; overlapping placements were merged, and the statistic was the fraction of their union overlapping IR sequence. The single-IR model removed IRb and reverse-mapped homologous intervals to IRa, yielding 14 intervals on a 160,216-bp circular reference; the same union-based statistic was used. The independent-event model randomized eight unitig-level MTPT blocks with unambiguous IR, LSC, or SSC assignments on the single-IR reference; overlaps were counted additively, and the statistic was total IR overlap divided by total event length. For each model, interval or event lengths were preserved and start positions randomized independently; the one-sided empirical *p* value was (E + 1)/(1,000,001), where E was the number of randomized statistics at least as large as the observed statistic. Sensitivity analyses with BLASTN v2.17.0+ used five length/identity thresholds: 100/80, 200/80, 500/80, 100/90, and 200/90 (bp/%).

### 2.7. Comparative Genomics and Sequence Synteny

Comparative analyses included *R. delavayi* (OQ868516), *R. decorum* (OQ450179), *R. vialii* (PQ537097), *R. nivale* subsp. *boreale* (PQ537102–PQ537104), *R. × pulchrum* (OM283814), and *R. simsii* (MW030508). GenBank gene annotations were reassessed using uniform BLASTN and TBLASTN searches against curated and supplemental gene queries. The results distinguished annotated loci, unannotated near-full or partial homologs, unresolved tRNA identities, and loci without a reliable match. Because annotation completeness differs among deposited genomes, apparent absences were treated as annotation-level differences rather than confirmed gene losses. MUMmer v3.23 (NUCmer v3.1) [15] alignments used --mumreference, a 100-bp minimum exact match, and a 500-bp minimum cluster. Blocks ≥ 500 bp with ≥80% identity were retained to calculate mean reciprocal coverage and to classify the *R. spinuliferum* sequence as aligned to all six taxa, one to five taxa, or none under these thresholds. Coverage was calculated from merged intervals, and synteny plots displayed blocks ≥ 5 kb according to alignment orientation. For multipartite assemblies, aligned intervals were merged separately within each sequence component; focal interpath homology was masked, and graph-coordinate de-duplication was examined in sensitivity analyses. Synteny blocks were plotted within their actual components without joining across artificial concatenation boundaries. To assess sequence conservation across the seven taxa, retained alignments were evaluated separately for each taxon: positions aligned to all six other taxa were classified as core, those aligned to one to five taxa as shared, and those without a retained match as unmatched. Overlapping aligned intervals were merged for each comparison, and the resulting partitions were expressed as percentages of the analyzed sequence length. The focal taxon used the 784,118-bp comparison representation after masking 2475 bp of interpath homology; these fractions are not fully graph-deduplicated sequence proportions.

### 2.8. Predicted RNA Editing and RNA-seq Support

C-to-U editing sites were predicted in mitochondrial PCGs with Deepred-Mt v0.1.0 [24] using the angiosperm mitochondrial model and a score threshold of ≥0.50; predicted sites were classified by the codon effect. Two public RNA-seq libraries (SRR13338383 and SRR13338437) were trimmed with fastp v1.3.6 [25] and mapped to curated PCG transcript references with minimap2 v2.31-r1302 in short-read mode [16]. SAMtools v1.21 mpileup [18] counted bases with mapping and base qualities ≥ 30. Support for a predicted editing site required a C + T depth ≥ 20, at least three T reads, and T/(C + T) ≥ 0.05 in at least one library. The 12 mature exon junctions of *nad1*, *nad2*, and *nad5* were examined using primary alignments with MAPQ ≥ 30 and at least 20 aligned bases on each side of the junction. Because the RNA-seq libraries were not paired with DNA from the HiFi assembly individual, they were treated as species-level transcript evidence rather than individual-matched validation.

### 2.9. Comparative Mapping of RNA-Editing Candidates

RNA-supported sites were mapped to the six congeners listed in Section 2.7. Identical CDS copies were collapsed. Pseudogenes, unresolved locations, ambiguous bases, incomplete codons, CDS lengths outside 80–120% of the focal sequence, and genes with multiple distinct CDS copies were excluded. CDSs were translated with genetic code 1, with stop symbols replaced by X. Protein and nucleotide sequences were aligned independently with MAFFT v7.490 (--localpair --maxiterate 1000) [26]. Sites were retained only when protein-guided codon mapping and nucleotide alignment agreed and were then classified as retained C, genomic T, another base, or unresolved. C-retention proportions excluded unresolved comparisons, and intersections required resolution in all six taxa. These analyses compared genomic bases and did not assess RNA-editing activity in the congeners.

### 2.10. Mitochondrial PCG Phylogeny

Eighteen shared mitochondrial PCGs from 14 Ericaceae taxa and *Camellia sinensis* were translated, aligned with MAFFT v7.490 (--auto) [26], and concatenated. Maximum-likelihood inference was performed with IQ-TREE v2.0.7 using ModelFinder (MFP + MERGE), 1000 ultrafast bootstrap replicates, and 1000 SH-aLRT replicates [27,28,29,30]. *C. sinensis* served as the outgroup.

### 2.11. Mitochondrial-Chloroplast Topology Comparison

For the 11 taxa represented in both organellar datasets, 47 chloroplast PCGs were aligned as nucleotides with MAFFT v7.526 (--nuc --localpair --maxiterate 1000) [26] and concatenated. IQ-TREE v3.1.2 [31] was run using the model-selection and support settings described in Section 2.10. The mitochondrial tree was pruned to the same taxon set, and topological similarity was quantified using the unrooted Robinson–Foulds distance and the number of shared bipartitions.

## 3. Results

### 3.1. Assembly Structure and Representative Mitochondrial Paths

**Figure 1:**
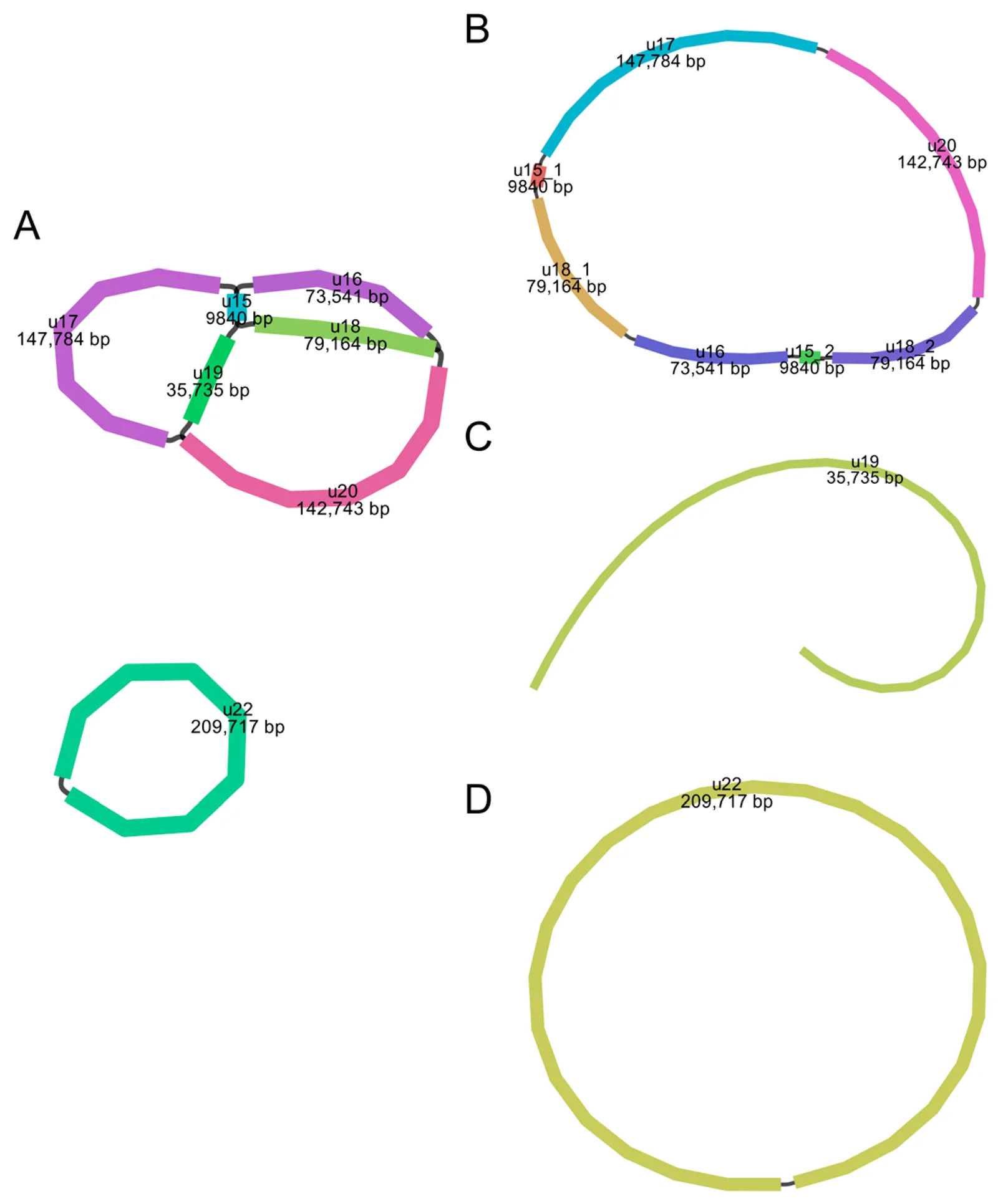
Long-read assembly graph and representative mitochondrial paths of *Rhododendron spinuliferum*. (**A**) Oatk mitochondrial graph showing the branched component and the separate u22 component. (**B**–**D**) Expanded representations of ctg1, ctg2, and ctg3. Segment labels indicate raw graph-segment lengths.

The assembly comprised a branched component and a separate self-connected component (Figure 1). The GFA contained seven segments and nine links, with branching connections involving u15, u18, and u20 and a separate u22 self-loop. Three representative paths totaled 786,593 bp: circular ctg1 (541,767 bp), linear ctg2 (35,735 bp), and circular ctg3 (209,091 bp). Collapsing 2475 bp of cross-path homology yielded a sequence-equivalent span of 784,118 bp. Primary HiFi alignments covered all bases, and independently generated Illumina reads from the second individual aligned to 94.2–99.2% of each path (Figure S1), providing cross-individual sequence support. Median per-base depths for ctg1, ctg2, and ctg3 were 724×, 724×, and 804× for HiFi reads and 58×, 66×, and 69× for Illumina reads, respectively (Figure S1). A cross-individual screen identified 148 SNPs and 23 small-indel candidates in 567,929 bp with adequate depth in both datasets and without ambiguous sequence contexts. The reported 94.2–99.2% Illumina coverage is the proportion of each path with ≥1 mapped read under the original permissive mapping settings, not sequence identity or stringent callable breadth. Most additional loss of coverage after MAPQ ≥ 30 filtering occurred in repeated or homologous regions; coverage gaps alone were not considered evidence of structural differences.

ctg1 traversed u15 and u18 twice, whereas ctg2 and ctg3 corresponded to u19 and u22, respectively.

### 3.2. Gene Content and Distribution Across Paths

**Figure 2:**
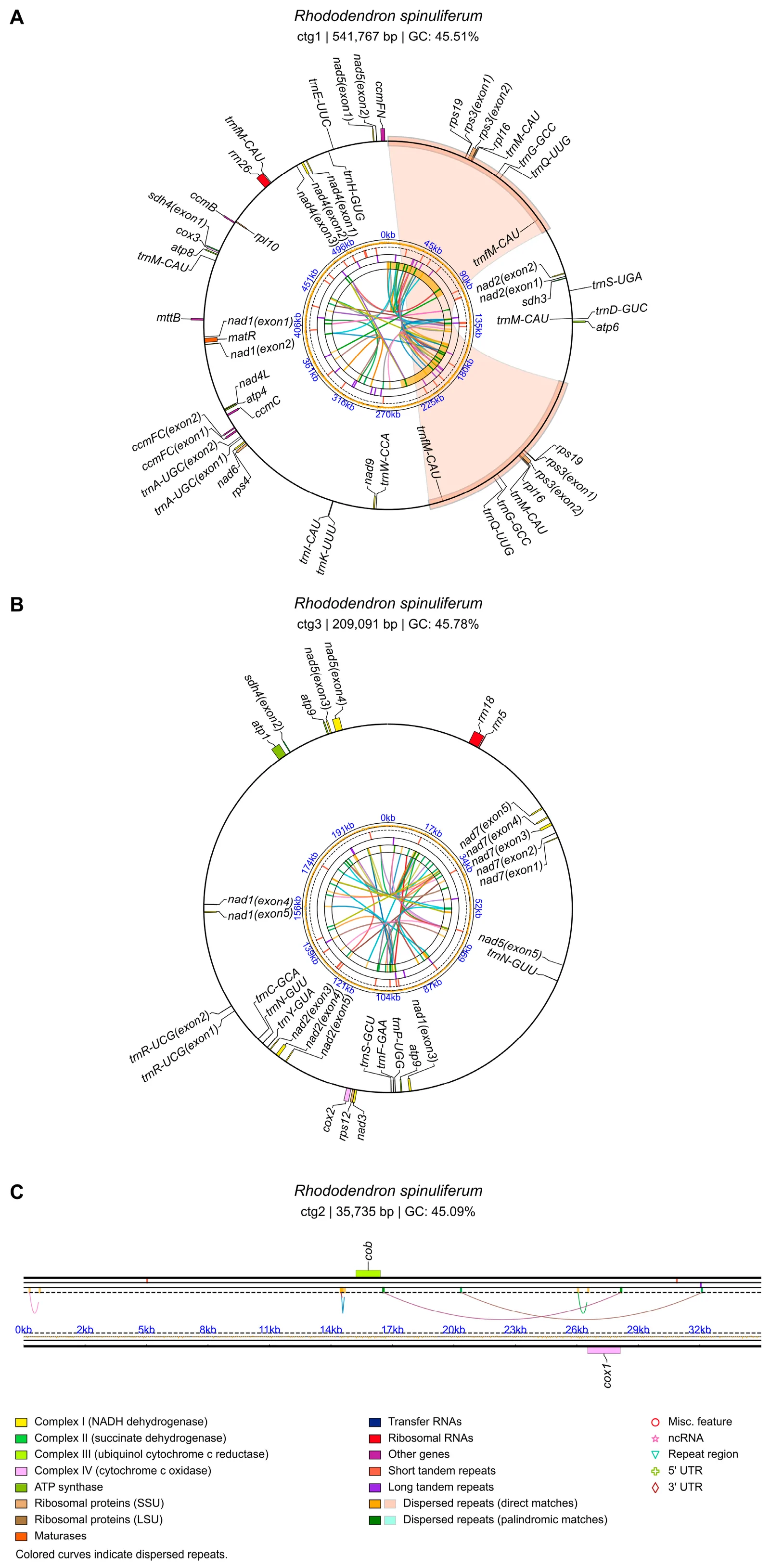
Gene maps of the three representative *Rhododendron spinuliferum* mitochondrial paths. (**A**) Circular ctg1 (541,767 bp). (**B**) Circular ctg3 (209,091 bp). (**C**) Linear ctg2 (35,735 bp). The pale-orange shaded regions in (A) highlight the two occurrences of the 79,164-bp u18 graph segment in the ctg1 assembly path.

Annotation identified 32 distinct protein-coding loci, three rRNA genes, and 19 tRNA types represented by 27 annotated tRNA loci (Figure 2; Table 1). GC contents were 45.51%, 45.09%, and 45.78% for ctg1, ctg2, and ctg3, respectively. ctg2 contained *cob* and *cox1*, whereas exons of *nad1*, *nad2*, and *nad5* were distributed between ctg1 and ctg3. Detailed gene-copy annotations, genomic coordinates, exon structures, and coding features are provided in Supplementary Table S3.

### 3.3. Long-Read Support for Alternative Repeat-Associated Connections

**Figure 3:**
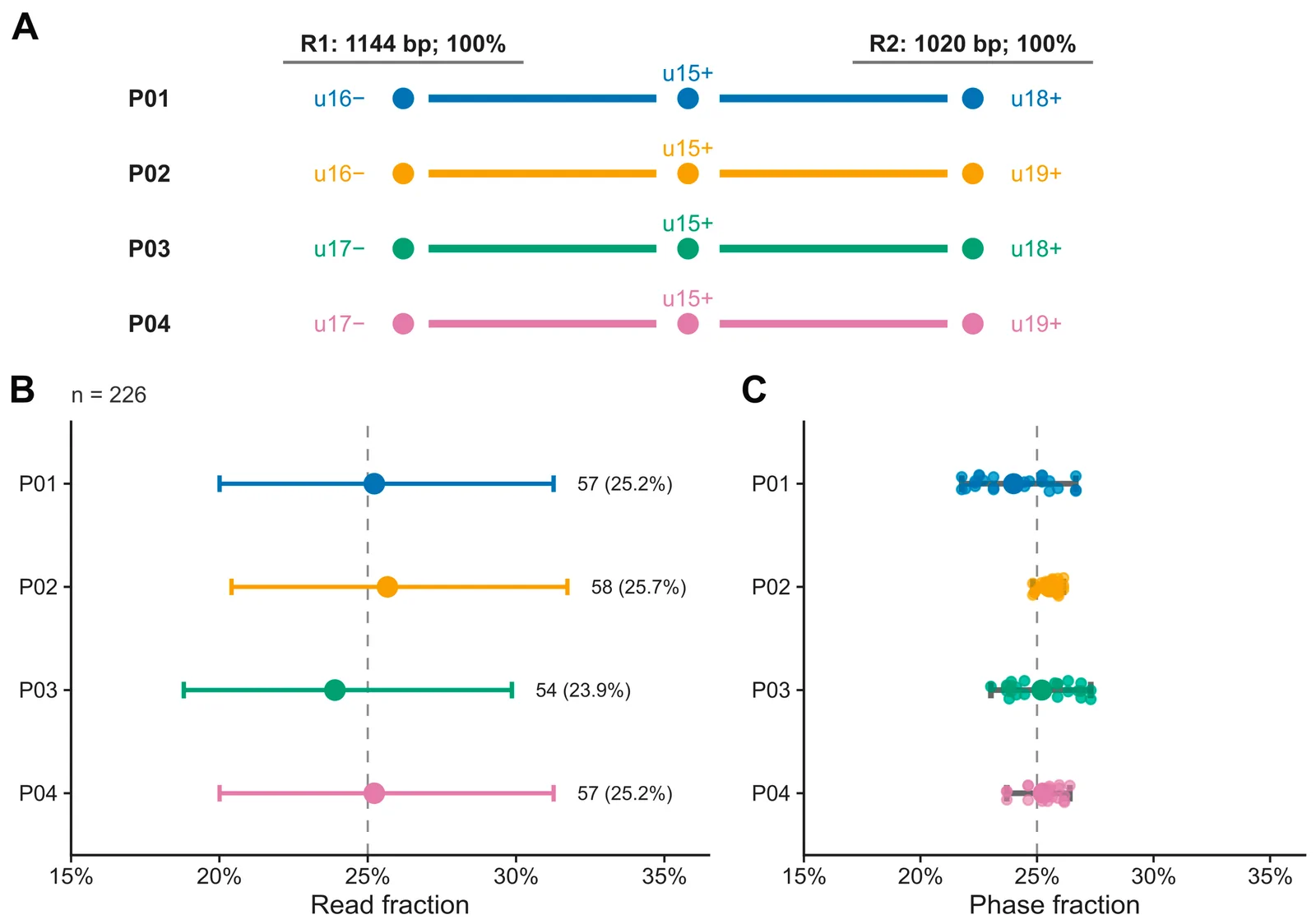
Repeat-associated junction configurations and strict two-junction phasing. (**A**) Candidate local phases P01–P04 around u15; R1 and R2 denote inter-unitig homologies between u16/u17 and u18/u19. (**B**) Fractions of retained reads after competitive mapping (MAPQ ≥ 30, identity ≥ 99.5%, span ≥ 11 kb); whiskers indicate Wilson 95% confidence intervals. (**C**) Sensitivity of phase fractions across 24 filtering combinations. Dashed lines indicate 25%. Wilson intervals assume independent retained reads and do not account for possible dependence among reads from the same mitochondrial molecule or repeat-associated regions. They are descriptive intervals for retained-read fractions, not estimates of mitochondrial-molecule abundance.

Two identical repeat pairs, 1144 bp (u16/u17) and 1020 bp (u18/u19), were associated with four local configurations around u15 (Figure 3A). Under the strict two-junction criteria, 226 HiFi reads were retained: 57, 58, 54, and 57 supported P01–P04, respectively (Figure 3B).

All four configurations were retained across the 24 filtering combinations, with 135–271 qualifying reads among parameter sets (Figure 3C). An audit of the 226 retained reads found no duplicate read IDs; median read lengths were 16.25–17.73 kb, with a median MAPQ of 40 and median sequence identities of 99.93–99.95% across configurations. The 24 parameter combinations yielded 12 distinct read sets. Independent remapping and unique-flank checks confirmed all 226 assignments (minimum ΔAS, 244; independent BLASTN, 226/226). In empirical-profile simulations, strict retention rates were 74.50–75.96% across configurations (χ^2^ test, *p* = 0.707), with no observed misassignments among retained reads.

### 3.4. Interspersed Repeats and Simple Sequence Repeats

**Figure 4:**
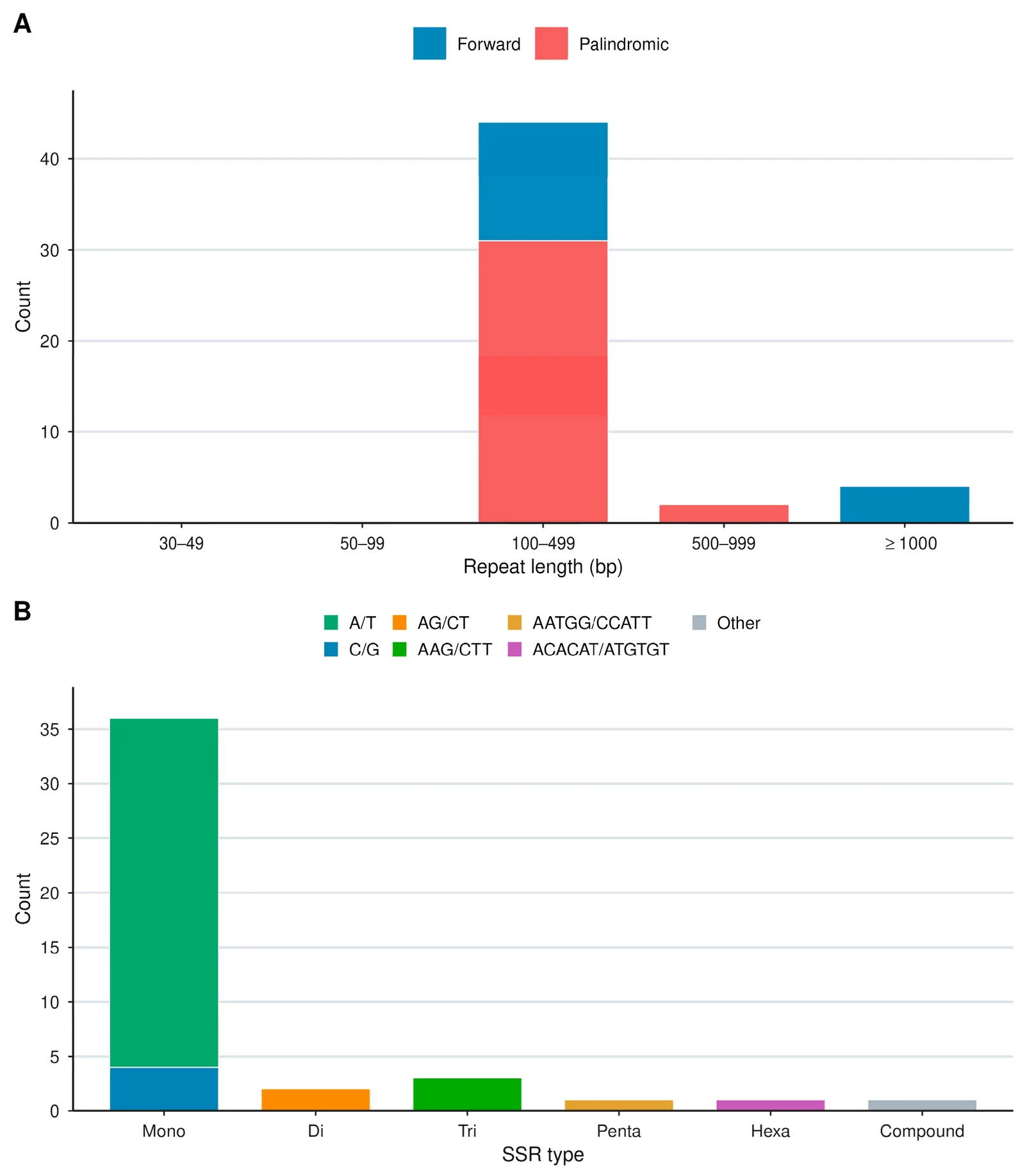
Interspersed-repeat and SSR composition in the *Rhododendron spinuliferum* mitochondrial assembly. (**A**) Counts of forward and palindromic repeat pairs by length class. (**B**) SSR counts by repeat-unit class and motif group.

The 50 reported Vmatch repeat-pair records comprised 17 forward and 33 palindromic pairs, including 44 pairs of 100–499 bp (Figure 4A). Two palindromic pairs were 500–999 bp, and four forward pairs were ≥1000 bp. The 44 SSR loci comprised 36 mononucleotides, two dinucleotides, three trinucleotides, one pentanucleotide, one hexanucleotide, and one compound locus (Figure 4B). Among these records, 13 showed reliable support for alternative local connections (eight nonredundant graph-coordinate groups), 35 had insufficient evidence, and two could not be evaluated (Table S2). The u15 region was selected for two-junction phasing because its approximately 9.8-kb segment allowed individual HiFi reads to establish how both flanking connections were paired. The 1144-bp u16/u17 repeat could not be distinguished by isolated repeat-flanking tests, but the combined two-junction analysis resolved the four local configurations.

### 3.5. Plastid-Derived Sequences in the Mitochondrial Assembly

**Figure 5:**
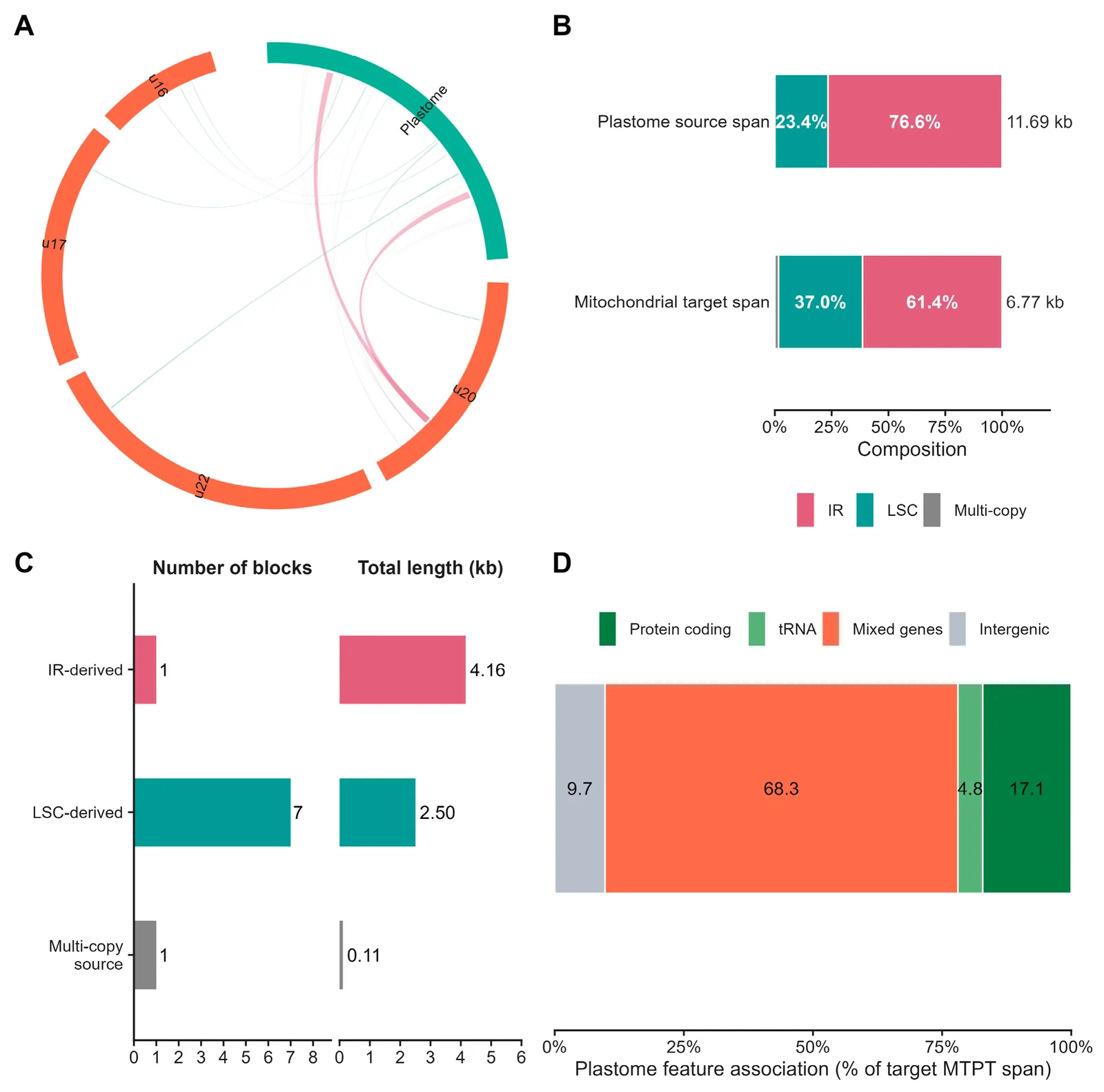
Plastid-derived sequence matches in the *Rhododendron spinuliferum* mitochondrial assembly. (**A**) Links between mitochondrial unitigs and homologous plastome intervals. (**B**) Composition of plastome source-coordinate span and mitochondrial target span by plastome region. (**C**) Number and total mitochondrial length of merged MTPT blocks assigned to IR, LSC, or multi-copy sources. (**D**) Plastome annotation categories represented within the mitochondrial target span. IR, inverted repeat; LSC, large single-copy region.

Nine MTPT blocks covered 6.77 kb: seven LSC-derived blocks totaling 2.50 kb, one IR-derived block of 4.16 kb, and one multi-copy-source block of 0.11 kb (Figure 5). The IR-derived block accounted for 61.4% of the mitochondrial target span. Of the 11.69-kb plastome source-coordinate span, 76.6% was IR-derived and 23.4% was LSC-derived. Mixed-gene plastome source regions accounted for 68.3% of the mitochondrial target span. IR-source enrichment was not significant under any tested null model or alignment threshold (*p* = 0.182–0.292).

### 3.6. Gene and Sequence Conservation Among Sampled Rhododendron Mitogenomes

**Figure 6:**
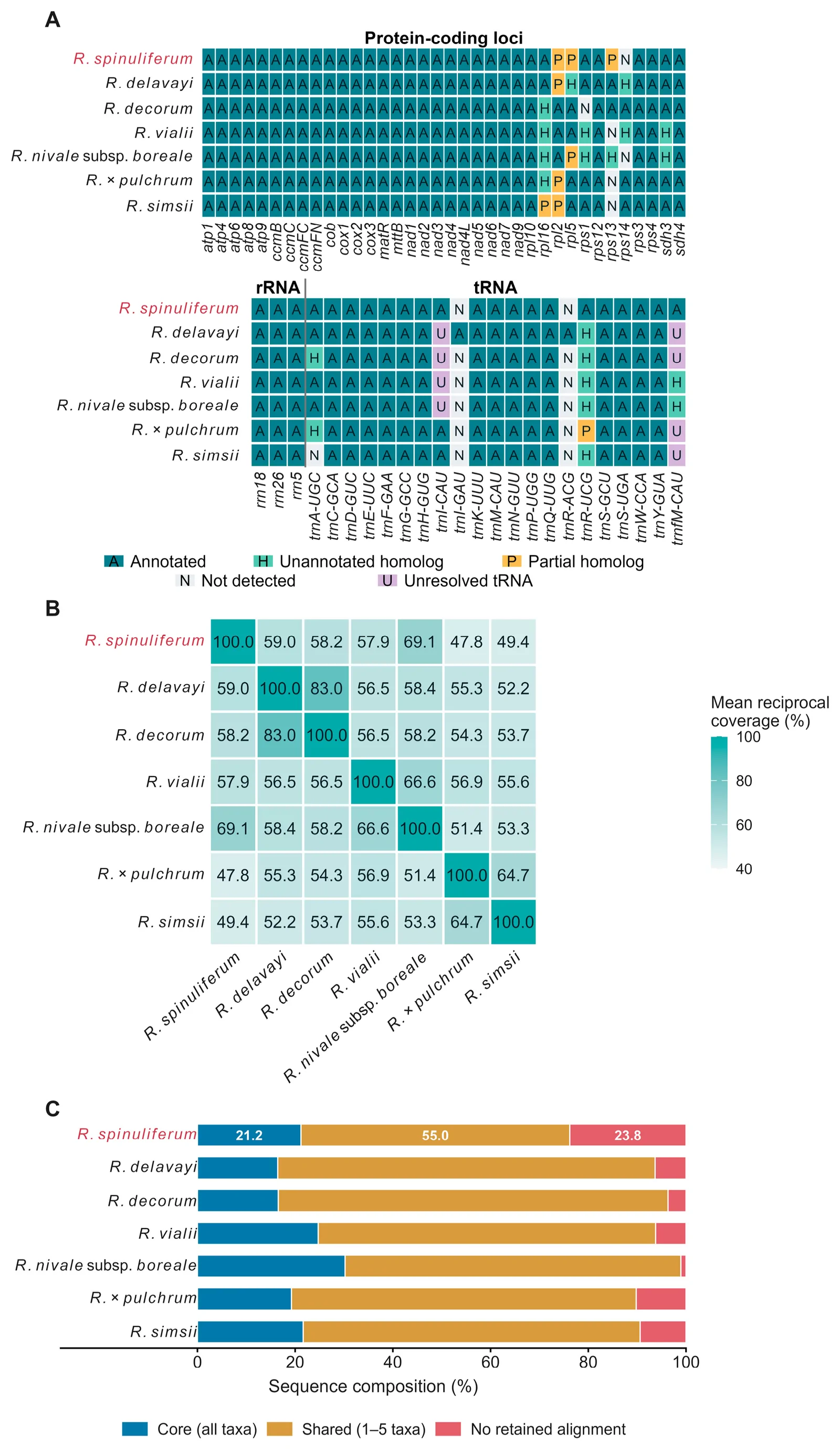
Gene annotation, homologous-sequence representation, and sequence conservation among seven sampled *Rhododendron* mitogenomes. (**A**) Annotation and homology-based matrix for protein-coding, rRNA, and tRNA loci (A, annotated; H, unannotated near-full homolog; P, partial homolog; U, unresolved tRNA identity; N, no reliable homolog detected). (**B**) Pairwise mean reciprocal alignment coverage among the seven mitogenomes. (**C**) Proportions of each mitogenome aligned to all six other taxa, to one to five taxa, or to none under the applied thresholds. *Rhododendron spinuliferum* is highlighted in red.

Uniform homology screening identified unannotated near-full homologs and partial sequences at several loci absent from deposited gene annotations (Figure 6A). For comparisons involving the focal mitogenome, mean reciprocal sequence coverage ranged from 47.8% to 69.1%, with the highest value for *R. nivale* subsp. *boreale* (Figure 6B). Of the 784,118-bp comparison sequence, 21.2% aligned to all six taxa, 55.0% aligned to one to five taxa, and 23.8% had no retained alignment under the applied thresholds (Figure 6C). Recalculation with separate assembly components yielded almost identical reciprocal coverage estimates (maximum difference, 0.074 percentage points).

### 3.7. Whole-Genome Synteny

**Figure 7:**
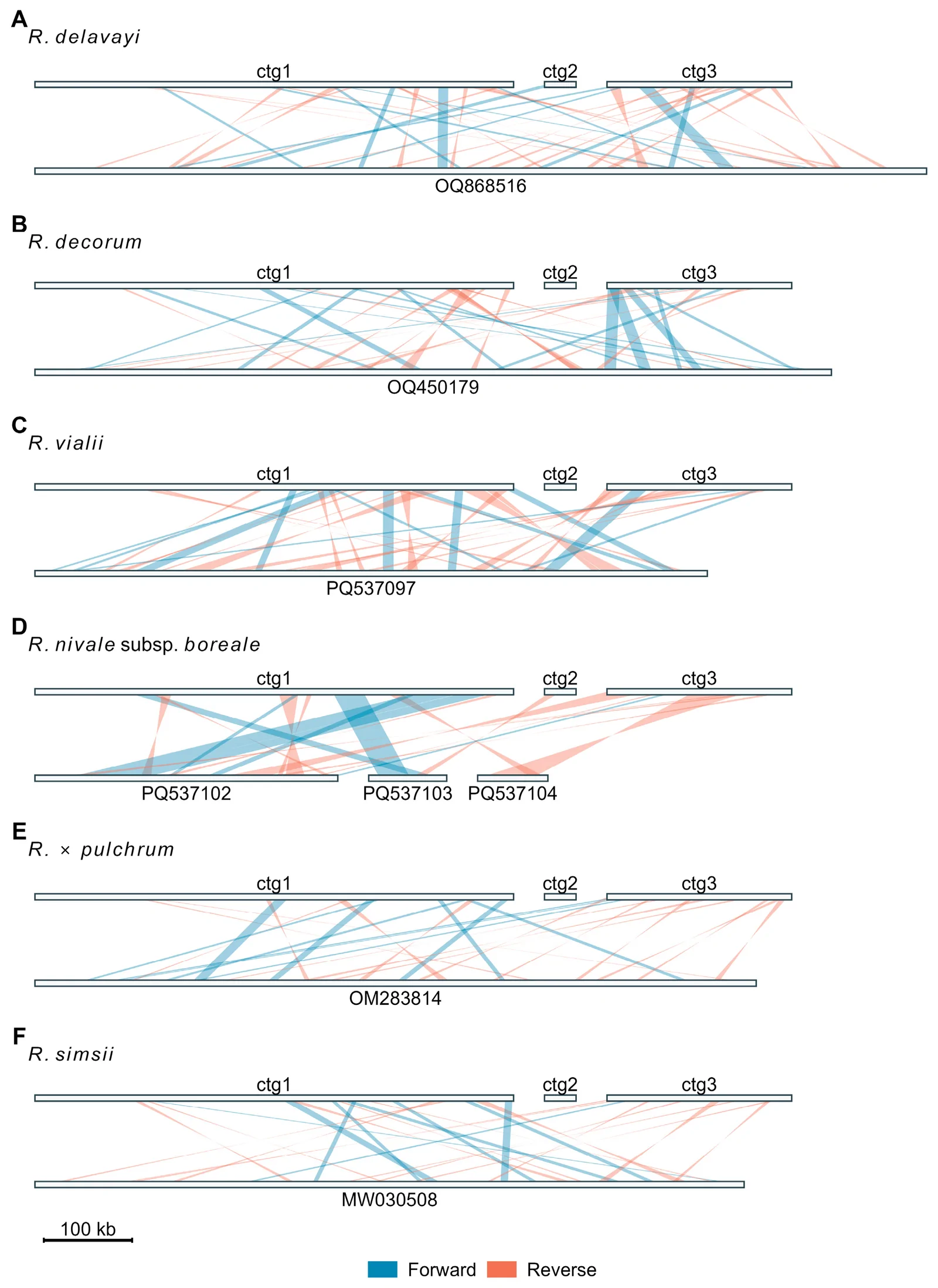
Local homologous-sequence blocks between *Rhododendron spinuliferum* and six sampled congeners. Panels compare *R. spinuliferum* with (**A**) *R. delavayi*, (**B**) *R. decorum*, (**C**) *R. vialii*, (**D**) *R. nivale* subsp. *boreale*, (**E**) *R. × pulchrum*, and (**F**) *R. simsii*. Blue and red ribbons indicate forward- and reverse-orientation alignments, respectively. Separate bars represent individual assembly components; only blocks ≥ 5 kb are displayed, and no connections across component boundaries are implied.

Pairwise comparisons recovered 20–38 local homologous blocks meeting the display threshold (≥5 kb), with blocks in both orientations (Figure 7). *R. delavayi* had the most blocks (38), whereas *R. nivale* subsp. *boreale* had the fewest (20). Opposite-orientation blocks predominated in all six comparisons; *R. decorum* had 16 same-orientation blocks and 18 opposite-orientation blocks. The displayed blocks are not one-to-one collinear chains.

### 3.8. Predicted Mitochondrial RNA Editing

**Figure 8:**
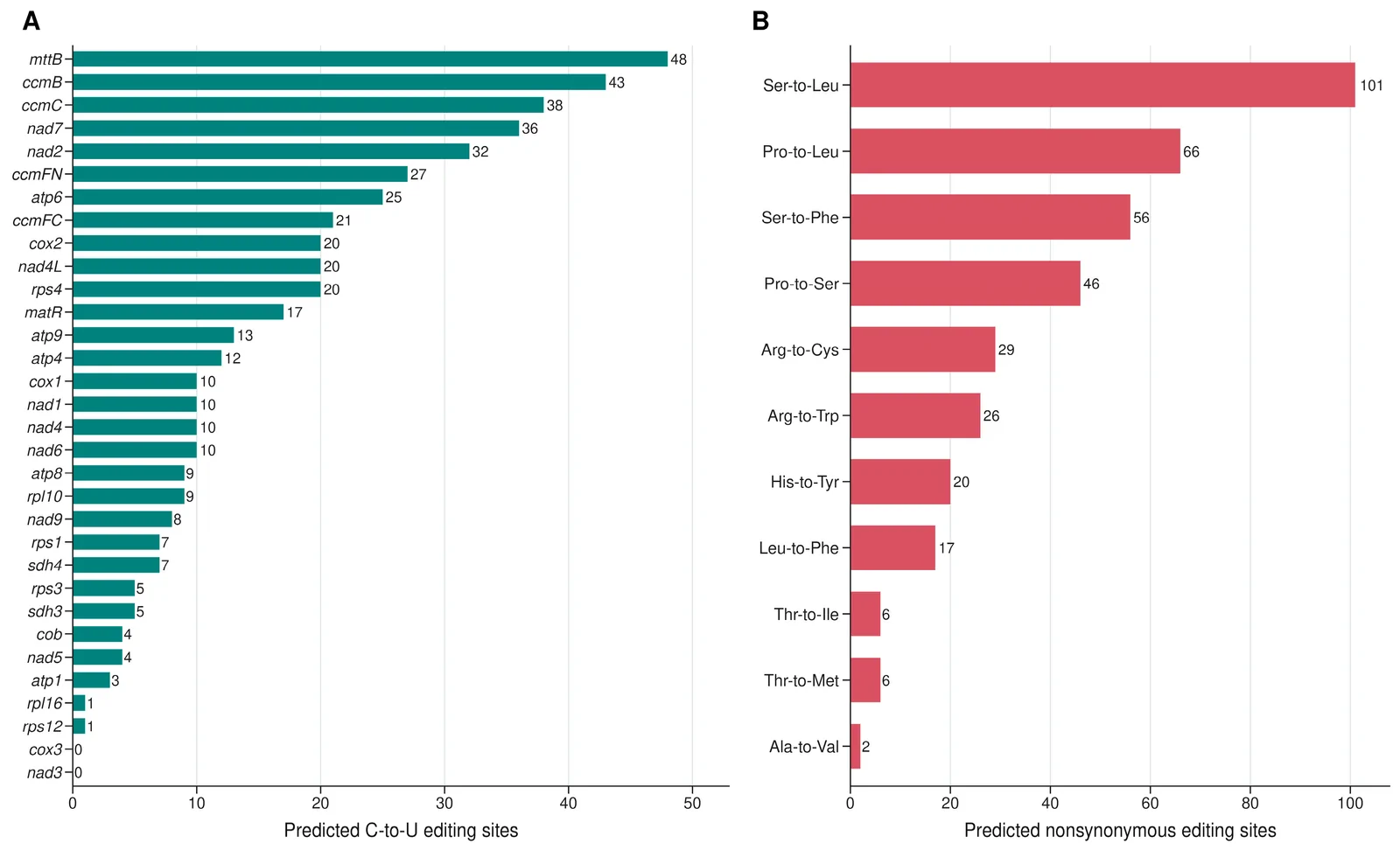
Predicted mitochondrial C-to-U RNA-editing sites in *Rhododendron spinuliferum*. (**A**) Candidate counts by unique protein-coding gene. (**B**) Frequencies of predicted nonsynonymous amino-acid changes. Predictions with scores ≥ 0.50 were retained.

Deepred-Mt predicted 475 C-to-U editing sites: 375 nonsynonymous (78.9%), 96 synonymous (20.2%), and four stop-gain candidates (0.8%; Figure 8). *mttB* contained the most candidates (48), followed by *ccmB* (43) and *ccmC* (38). The most frequent predicted nonsynonymous changes were Ser → Leu (101), Pro → Leu (66), Ser → Phe (56), and Pro → Ser (46).

### 3.9. RNA-seq Support for Editing Candidates and Mature Exon Junctions

**Figure 9:**
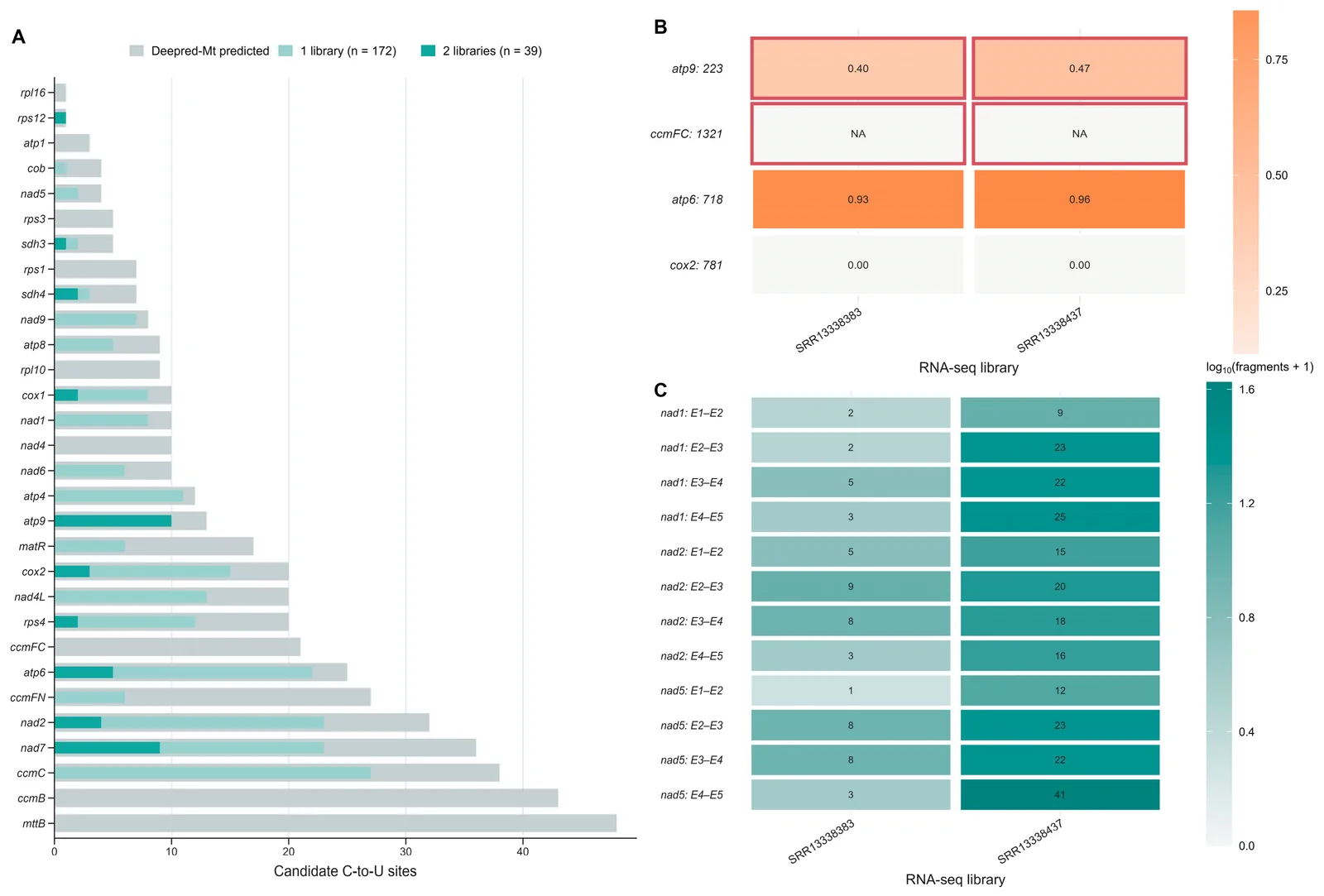
RNA-seq support for editing candidates and mature exon junctions. (**A**) Predicted editing sites by gene, distinguishing sites supported in exactly one library (172) from those supported in both libraries (39). (**B**) T/(C + T) fractions at four predicted stop-gain positions. NA indicates sites that could not be evaluated under the applied read-depth and quality criteria. (**C**) Numbers of junction-spanning fragments supporting the 12 examined exon pairs of *nad1*, *nad2*, and *nad5*.

RNA-seq data supported 211 of the 475 predicted editing sites (44.4%): 172 were supported in one library and 39 in both libraries (Figure 9A). Under the C + T depth ≥ 20 criterion, 70 sites were evaluable in SRR13338383 (49 supported; 70.0%) and 256 in SRR13338437 (201 supported; 78.5%). In total, 266 sites were evaluable in at least one library, of which 211 were supported (79.3%; Table S1). In SRR13338383 and SRR13338437, respectively, T/(C + T) fractions were 0.40 and 0.47 at *atp9* position 223 and 0.93 and 0.96 at *atp6* position 718. *cox2* position 781 had no T reads in either library, whereas *ccmFC* position 1321 could not be evaluated (Figure 9B). Junction-spanning fragments were detected for all 12 examined mature exon junctions of *nad1*, *nad2*, and *nad5* in both libraries. Support ranged from 1–9 fragments per junction in SRR13338383 and 9–41 in SRR13338437 (Figure 9C).

### 3.10. Genomic Conservation of RNA-Supported Editing Sites

**Figure 10:**
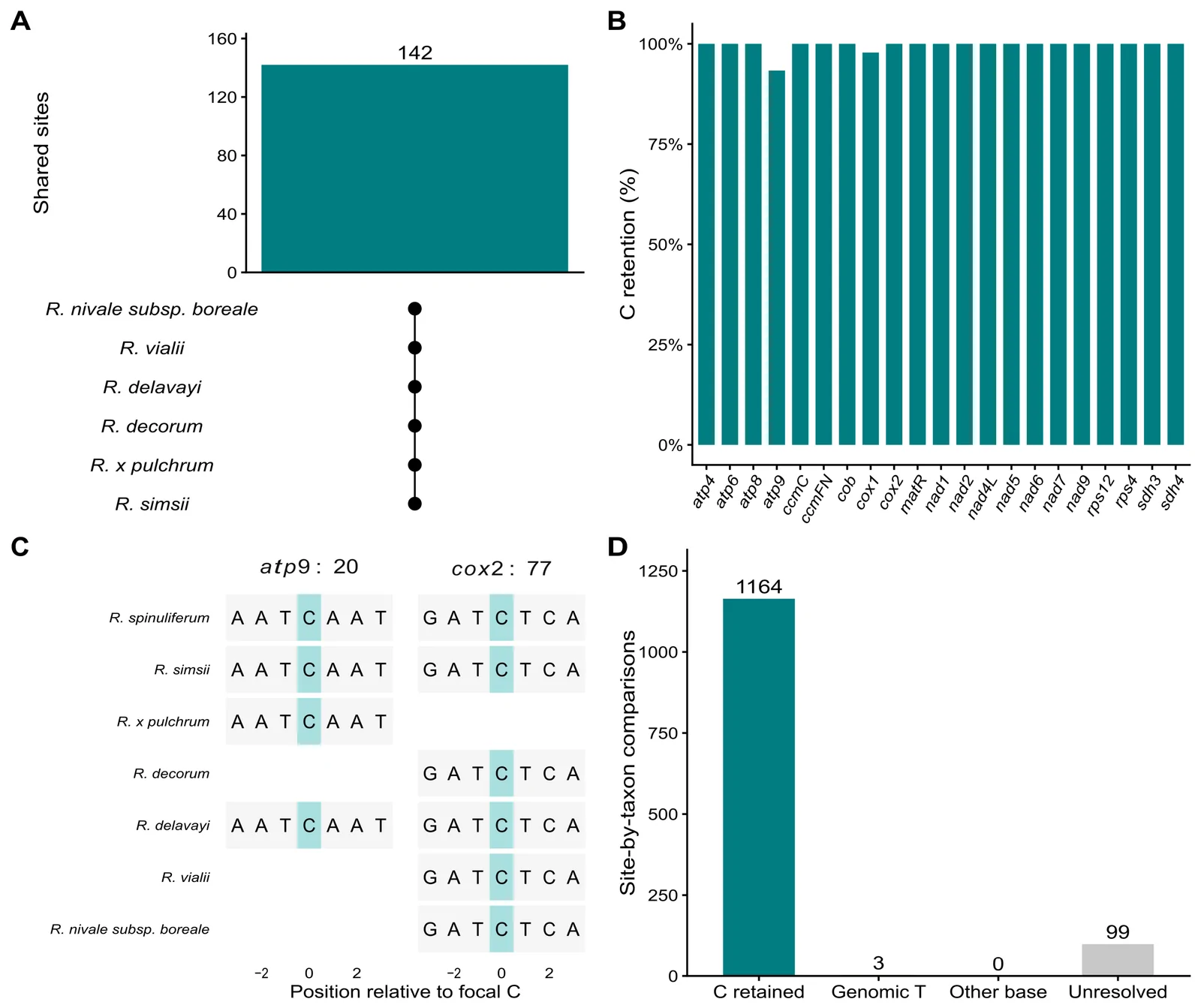
Genomic conservation of RNA-supported editing-candidate positions in *Rhododendron*. (**A**) Intersections of retained-C sites among the six comparator taxa, restricted to positions resolved in every taxon. (**B**) C-retention proportion by gene among resolved site-by-taxon comparisons. (**C**) Aligned nucleotide contexts around *atp9* position 20 and *cox2* position 77; shading marks the focal position. Coordinates refer to the *R. spinuliferum* CDS. (**D**) Genomic-base categories across all 1266 comparisons. Unresolved comparisons include unavailable or excluded CDSs and discordant or gapped mappings. RNA-seq support was established only for *R. spinuliferum*.

The 211 RNA-supported sites yielded 1266 site-by-taxon comparisons across the six congeners (Figure 10). Of 1167 resolved comparisons, 1164 retained genomic C (99.7%) and three contained genomic T; ninety-nine were unresolved. Genomic T occurred at positions homologous to *atp9* site 50 in *R. simsii* and *R. × pulchrum* and to *cox1* site 11 in *R. × pulchrum*.

### 3.11. Mitochondrial Phylogeny and Comparison with the Plastid Tree

**Figure 11:**
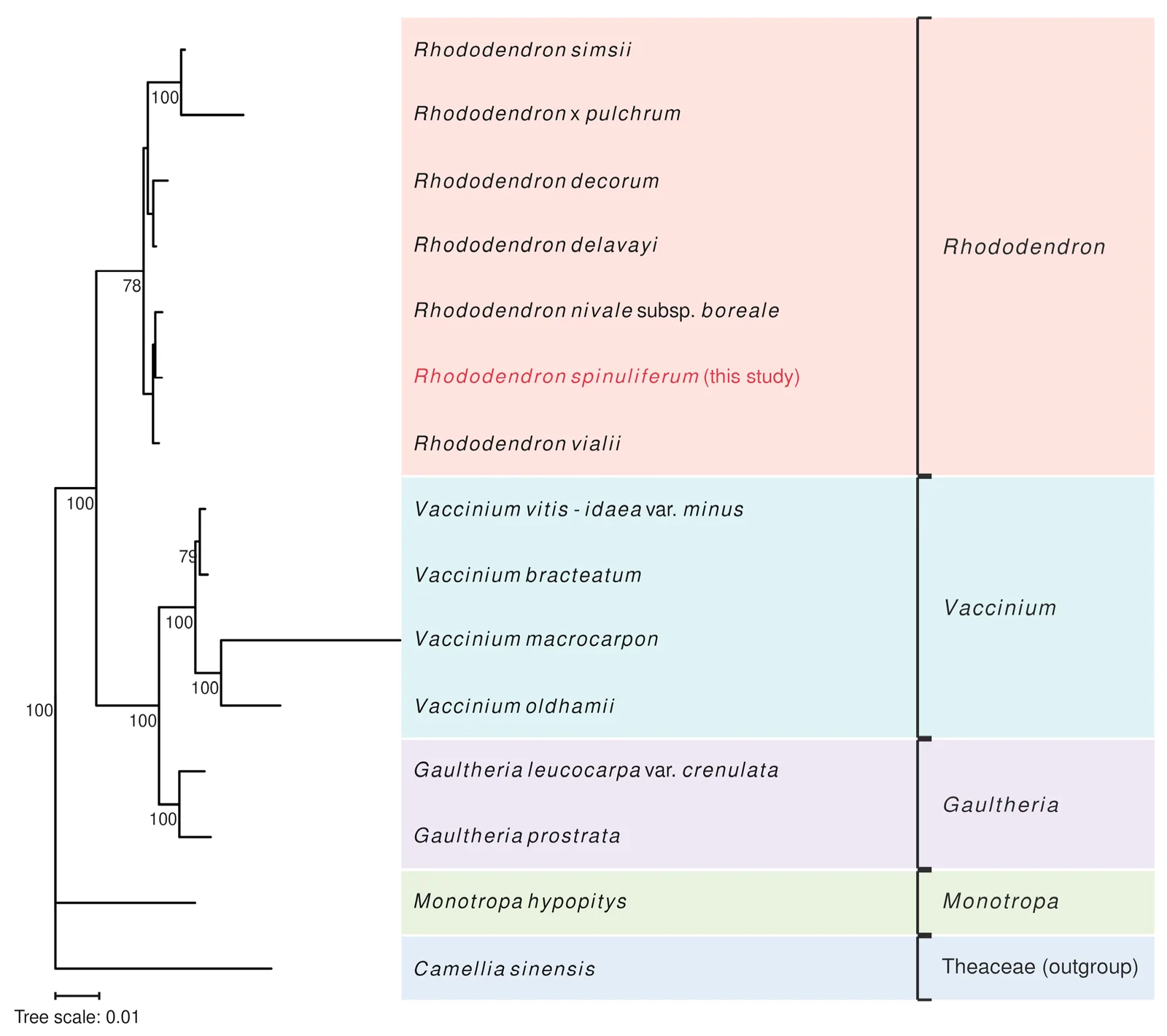
Maximum-likelihood mitochondrial phylogeny of sampled Ericaceae taxa based on 18 shared protein-coding genes (6635 amino-acid positions). *Camellia sinensis* was used as the outgroup. Node values indicate bootstrap support. *Rhododendron spinuliferum* is highlighted in red.

**Figure 12:**
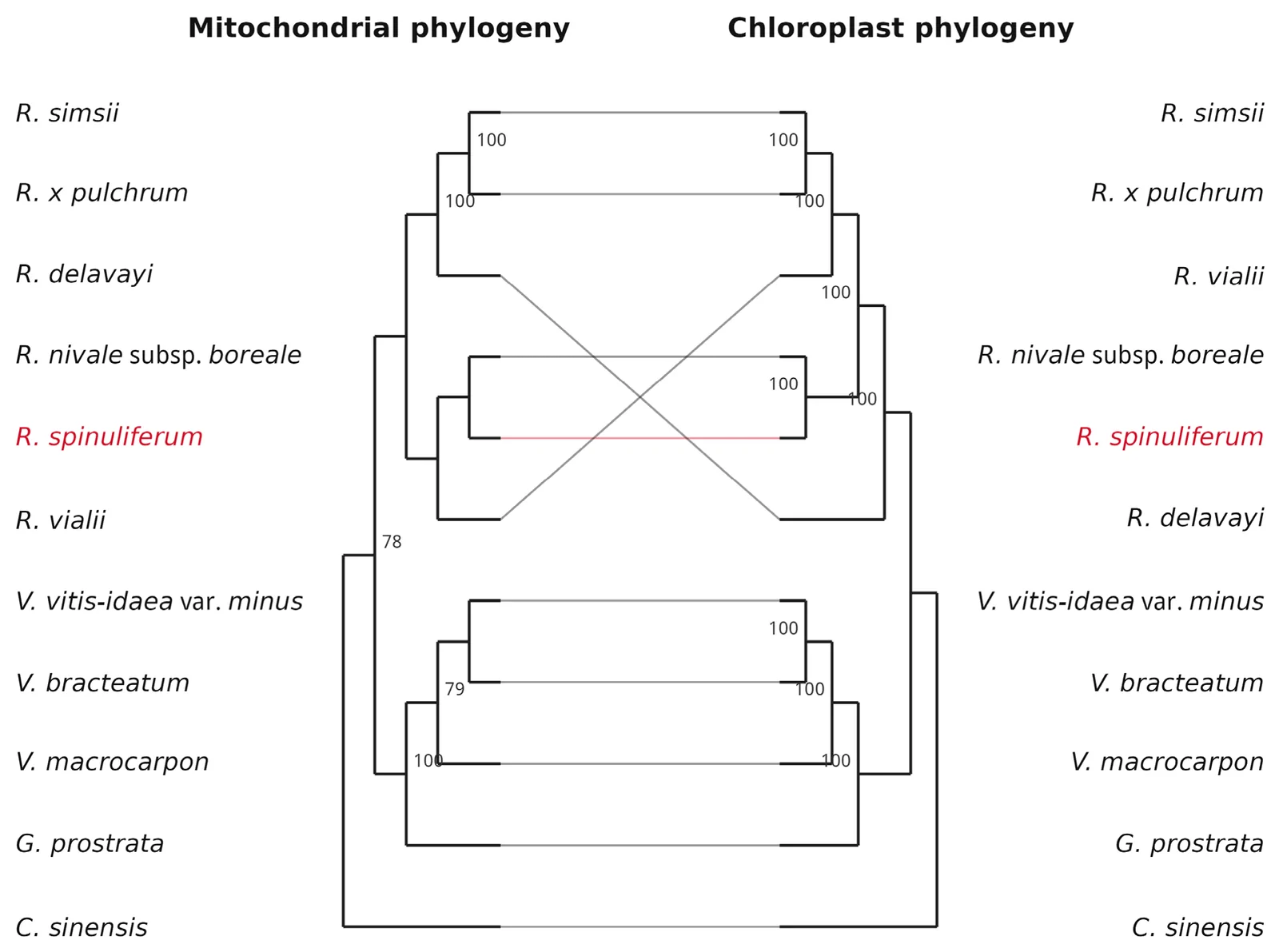
Comparison of mitochondrial and chloroplast PCG phylogenies for 11 matched taxa. Gray lines connect corresponding taxa between the two trees; the red connecting line and red taxon labels highlight *Rhododendron spinuliferum*. Node values indicate ultrafast bootstrap support.

The 18-gene mitochondrial alignment contained 6635 amino-acid positions. The sampled *Rhododendron* taxa formed a clade with 78% bootstrap support, and *R. simsii* and *R. × pulchrum* formed a strongly supported pair (100%; Figure 11). Across the 11 taxa shared between the organellar datasets, the mitochondrial and plastid trees shared five bipartitions and had an unrooted Robinson–Foulds distance of four (normalized distance = 0.250). The placements of *R. delavayi* and *R. vialii* differed between the two trees (Figure 12).

## 4. Discussion

### 4.1. Interpreting the Mitochondrial Assembly in a Structurally Dynamic Genome

The mitochondrial assembly of *R. spinuliferum* is best interpreted as a set of graph-supported sequence paths rather than as a fixed collection of mitochondrial chromosomes. Repeated sequences can generate alternative mitochondrial configurations, making it important to distinguish assembly paths from physical molecules [6,32,33,34]. Previous *Rhododendron* studies have reported diverse assembly structures and, in some cases, long-read support for alternative configurations [12,13,14]. In the present study, the three representative paths provide stable coordinates for annotation and comparative analysis, but their circular or linear layout does not by itself define the physical organization of mitochondrial DNA in vivo. Direct structural evidence comes from reads spanning specific junctions. Recent long-read studies also distinguish assembled paths from directly supported connections [8,9,10,11,35].

### 4.2. Repeat-Associated Local Configurations Supported by Long Reads

The clearest evidence for structural heterogeneity in *R. spinuliferum* is the coexistence of four local configurations around u15. These configurations were associated with two identical repeat pairs and were each supported by HiFi reads spanning both diagnostic junctions. These reads establish local connectivity but do not demonstrate distinct full-length mitochondrial genome isoforms. Phasing both junctions within a single read reduces the ambiguity of evaluating each junction independently. All four configurations persisted across 24 combinations of alignment span, mapping quality, and sequence identity, suggesting that their detection did not depend on a single filtering threshold. Repeat-mediated alternative configurations have been reported in other plant mitogenomes using long-read or experimental validation [9,10,11,35], and the present data extend this pattern to *R. spinuliferum*. The approximately balanced retained-read counts are nevertheless a descriptive feature of this dataset, not a measurement of complete-molecule stoichiometry or recombination rate. Read length, DNA extraction, mapping, and unobserved distal connections may affect their relative representation. Additional mapping and simulation checks support robust discrimination of these local configurations under the tested conditions, but cannot rule out all sampling or mapping biases.

### 4.3. Gene Content, Split Genes, and Transcript Support

Core gene content was relatively conserved despite this structural complexity. The curated *R. spinuliferum* assembly contained 32 distinct protein-coding loci, similar to the 32 protein-coding genes reported for *R. delavayi* [13], whereas 33 were reported for *R. simsii* [12]. Small differences of this kind should be interpreted cautiously because duplicated copies, fragments, pseudogenes, and annotation practices can change reported gene counts without implying biological gene loss. The gene-representation matrix therefore combines deposited annotations with homology searches rather than treating missing annotations as confirmed gene losses. In *R. spinuliferum*, the exons of *nad1*, *nad2*, and *nad5* were distributed across assembly paths, a pattern compatible with the well-established trans-splicing of group II introns in plant mitochondria [36]. RNA-seq reads spanning all 12 examined mature exon junctions provide an independent link between the genomic annotation and processed transcripts. Junction support varied between libraries; these data support the presence of mature transcripts but do not quantify splicing efficiency.

### 4.4. Plastid-Derived Sequences in the Context of an Expanded Plastome IR

Plastid-derived DNA contributes to the mosaic composition of plant mitochondrial genomes [6,11,37]. Nine MTPT blocks were detected in *R. spinuliferum*, and a single 4.16-kb block derived from the plastome inverted repeat accounted for most of the mitochondrial target span. The *R. spinuliferum* plastome has an unusually expanded IR [5]. A large IR-derived mitochondrial match is therefore biologically plausible, but its size alone does not demonstrate preferential transfer from the IR. IR-source enrichment was not significant across the tested permutation models or alignment thresholds. Separating plastome source-coordinate span from mitochondrial target span avoids double counting the homologous sequence shared by the two IR copies. The data document plastid-homologous sequences in the mitochondrial assembly but do not establish preferential IR transfer or a causal link between MTPTs and the u15-associated configurations.

### 4.5. RNA Editing: Prediction, Transcript Evidence, and Conservation

C-to-U RNA editing is a major component of plant mitochondrial RNA maturation [38]. Deepred-Mt predicted 475 candidate sites in *R. spinuliferum*, of which 211 were supported by the two public RNA-seq libraries under the applied coverage and allele-fraction thresholds. RNA-seq provides empirical support for a subset of predictions, but the 44.4% support rate does not estimate the true proportion of edited sites. A site may lack support because of limited transcript coverage, tissue-specific expression, or absence of editing. Sites with a strong C-to-U signal in both libraries provide stronger transcript-level evidence. Because the RNA-seq libraries were not matched to the HiFi assembly individual, genomic polymorphisms cannot be excluded at every site. Of the four predicted stop-gain sites, *atp9*:223 and *ccmFC*:1321 occur at annotated terminal codons and may generate normal termination signals. In contrast, *atp6*:718 could truncate the predicted protein by 52 residues and met the RNA-seq support criteria in one library, whereas *cox2*:781 could remove five C-terminal residues but showed no T reads. These coding consequences remain hypothetical without individual-matched DNA/RNA or protein-level validation.

Cross-species comparisons assessed conservation of genomic bases at these sites. Among resolved homologous positions in six congeners, 99.7% retained genomic C, whereas only three comparisons contained genomic T. Thus, the DNA substrate corresponding to most RNA-supported candidate sites is highly conserved across the sampled *Rhododendron* mitogenomes. This pattern is consistent with conservation of editable genomic positions, but it does not demonstrate that the sites are edited in the other species. Testing conservation of editing activity requires comparable transcript data from those taxa. In these few cases, the nucleotide corresponding to the edited state in *R. spinuliferum* is already encoded by a congener.

### 4.6. Phylogenetic Context and Mitochondrial–Plastid Discordance

The mitochondrial phylogeny placed the sampled *Rhododendron* species within a single clade, but support for that clade was moderate (78%), and the mitochondrial and plastid trees differed in the positions of *R. delavayi* and *R. vialii*. Comparable organellar discordance has been reported in previous *Rhododendron* analyses [14]. The Robinson–Foulds comparison confirms topological discordance without identifying its cause. Mitochondrial and plastid datasets differ in gene number, evolutionary rate, and the amount and distribution of phylogenetic signal; biological processes such as introgression or organelle capture are additional possibilities, but they cannot be distinguished from analytical or sampling effects with organellar data alone. These phylogenies provide comparative context but do not resolve the basis of the discordance. Testing the biological basis of the discordance will require denser taxon sampling and independent nuclear-genome evidence.

### 4.7. Scope and Limitations

This study combines a long-read assembly graph, strict single-read phasing of repeat-associated junctions, comparative mitogenomics, and RNA-seq support for editing candidates and mature exon junctions. The primary HiFi assembly and the independently generated Illumina reads came from different individuals, so the short-read data provide cross-individual sequence support rather than validation of the exact assembled molecule set. The public RNA-seq libraries were likewise not individual-matched to the HiFi dataset. Intraspecific SNPs, indels, or structural polymorphisms may affect cross-individual mapping and the representation of local configurations. Genomic C/T polymorphisms in RNA-seq source individuals could also contribute to apparent editing signals, which cannot be excluded without matched DNA and RNA data. The phasing analysis resolves local connections rather than complete mitochondrial molecules, and gene-presence comparisons depend partly on public annotation quality. Within these limits, the results document local structural heterogeneity and provide transcript support for the mitochondrial annotation. Future work could use population-level datasets and individual-matched DNA/RNA evidence to investigate mitochondrial structural variation and RNA processing in *R. spinuliferum*.

## 5. Conclusions

This study provides three representative mitochondrial assembly paths for *R. spinuliferum*, totaling 786,593 bp and containing 32 distinct protein-coding loci, three rRNA genes, and 19 tRNA types. Strict single-read phasing supported four repeat-associated local configurations that remained detectable across multiple filtering thresholds. Public RNA-seq data supported 211 predicted editing sites and provided junction-spanning evidence for all 12 examined mature exon junctions of *nad1*, *nad2*, and *nad5*. Comparisons with congeners showed broadly conserved respiratory-gene representation but substantial variation in sequence organization. Together, these results expand mitochondrial genomic resources for *Rhododendron* while distinguishing representative assembly paths from read-supported local configurations.

## Figures and Tables

**Table 1:** Functional classification of curated mitochondrial gene types in *Rhododendron spinuliferum*.

Gene Class	Functional Category	Genes
Protein-coding genes	Complex I (NADH dehydrogenase)	*nad1*, *nad2*, *nad3*, *nad4*, *nad4L*, *nad5*, *nad6*, *nad7*, *nad9*
	Complex II (succinate dehydrogenase)	*sdh3*, *sdh4*
	Complex III (cytochrome bc_1_ complex)	*cob*
	Complex IV (cytochrome c oxidase)	*cox1*, *cox2*, *cox3*
	Complex V (ATP synthase)	*atp1*, *atp4*, *atp6*, *atp8*, *atp9*
	Cytochrome c biogenesis	*ccmB*, *ccmC*, *ccmFC*, *ccmFN*
	Maturase	*matR*
	Large-subunit ribosomal proteins	*rpl10*, *rpl16*
	Small-subunit ribosomal proteins	*rps3*, *rps4*, *rps12*, *rps1*
	Transport membrane protein	*mttB*
Ribosomal RNA genes	Ribosomal RNAs	*rrn5*, *rrn18*, *rrn26*
Transfer RNA genes	Transfer RNAs (19 distinct types; 27 annotated loci)	*trnA-UGC*, *trnC-GCA*, *trnD-GUC*, *trnE-UUC*, *trnF-GAA*, *trnfM-CAU*, *trnG-GCC*, *trnH-GUG*, *trnI-CAU*, *trnK-UUU*, *trnM-CAU*, *trnN-GUU*, *trnP-UGG*, *trnQ-UUG*, *trnR-UCG*, *trnS-GCU*, *trnS-UGA*, *trnW-CCA*, *trnY-GUA*

Gene types are counted once. The 32 annotated protein-coding loci exclude five additional fragment annotations; *rpl16* and *rps3* occur in duplicated copies, and exons of *nad1*, *nad2*, and *nad5* are distributed across paths.

## Data Availability

The mitochondrial assembly paths generated in this study are available in GenBank (NCBI; https://www.ncbi.nlm.nih.gov, accessed on 13 October 2026) under accession numbers PZ987783–PZ987785. The PacBio HiFi reads used for assembly are available from the Genome Sequence Archive under accession CRR1094297, and the Illumina reads are available from the NCBI Sequence Read Archive under accession SRR38226386. The plastome used for MTPT analysis is available under accession PZ603433, and the RNA-seq datasets are available under accessions SRR13338383 and SRR13338437.

## Supplementary Materials

The following supporting information can be downloaded at https://www.mdpi.com/article/10.3390/genes17101288/s1, Figure S1: Sequencing-depth profiles of the three representative mitochondrial paths: (A) ctg1, (B) ctg2, and (C) ctg3. Solid lines indicate mean depth in 5-kb windows; dashed lines indicate median per-base depth. PE400 refers to Illumina reads from a second individual. Table S1: Predicted mitochondrial C-to-U RNA-editing sites and RNA-seq support in *Rhododendron spinuliferum* (n = 475). Table S2: Interspersed repeat pairs and HiFi support for alternative local connections in *Rhododendron spinuliferum* (n = 50 Vmatch records). Table S3: Mitochondrial gene-copy annotation and exon coordinates in *Rhododendron spinuliferum*.
