# Supplementary material for: Long-Read Evidence for Repeat-Associated Structural Heterogeneity in the Mitochondrial Genome of Rhododendron spinuliferum
Source: Genes (Basel). 2026 Oct 15;17(10):1288. doi: 10.3390/genes17101288 (PMC13703991; doi:10.3390/genes17101288)
Supplement: Supplementary file 1 [file genes-17-01288-s001.zip › genes-4614732-supplementary.pdf]

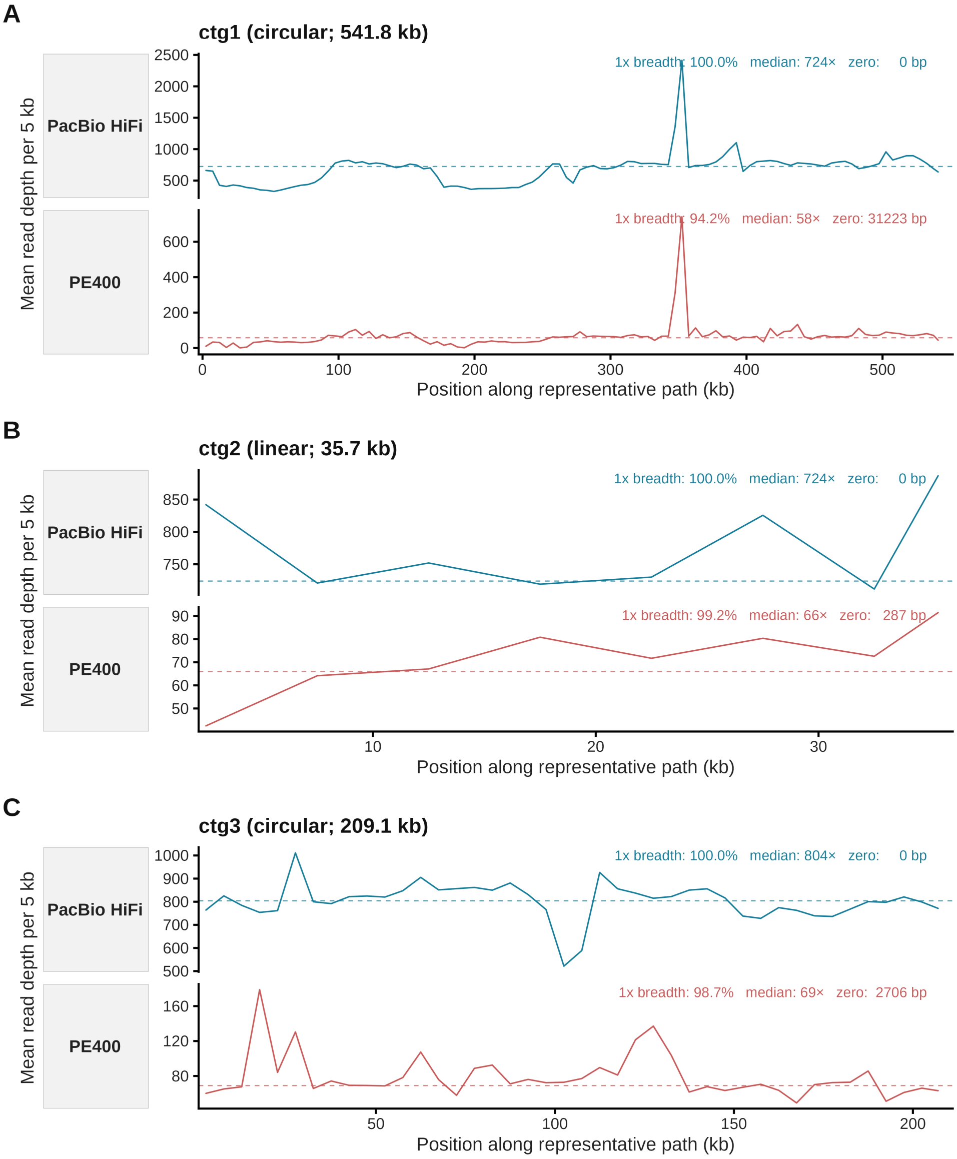

Figure S1. Sequencing-depth profiles of the three representative mitochondrial paths: (A) ctg1, (B) ctg2, and (C) ctg3. Solid lines indicate mean depth in 5-kb windows; dashed lines indicate median per-base depth. PE400 refers to Illumina reads from a second individual.

Table S1. Predicted mitochondrial C-to-U RNA-editing sites and RNA-seq support in *Rhododendron spinuliferum* (n = 475).

Site ID	Score	Codon effect	Codon change	AA change	L1 C/T	L1 T fraction	L1 support	L2 C/T	L2 T fraction	L2 support
atp1:69	0.627	synonymous	ACC>ACT	Thr-to-Thr	40/0	0.000	No	177/0	0.000	No
atp1:1041	0.523	synonymous	TCC>TCT	Ser-to-Ser	5/0	0.000	NE	49/0	0.000	No
atp1:1046	0.605	nonsynonymous	ACG>ATG	Thr-to-Met	5/0	0.000	NE	49/0	0.000	No
atp4:40	0.514	synonymous	CTA>TTA	Leu-to-Leu	2/0	0.000	NE	24/0	0.000	No
atp4:59	0.998	nonsynonymous	TCT>TTT	Ser-to-Phe	0/2	1.000	NE	0/30	1.000	Yes
atp4:71	0.997	nonsynonymous	TCA>TTA	Ser-to-Leu	0/2	1.000	NE	3/27	0.900	Yes
atp4:89	1.000	nonsynonymous	TCA>TTA	Ser-to-Leu	0/4	1.000	NE	0/33	1.000	Yes
atp4:118	0.974	nonsynonymous	CGT>TGT	Arg-to-Cys	0/4	1.000	NE	0/32	1.000	Yes
atp4:138	0.790	synonymous	ATC>ATT	Ile-to-Ile	1/3	0.750	NE	5/26	0.839	Yes
atp4:215	0.999	nonsynonymous	TCG>TTG	Ser-to-Leu	1/8	0.889	NE	1/39	0.975	Yes
atp4:248	1.000	nonsynonymous	CCT>CTT	Pro-to-Leu	1/7	0.875	NE	1/38	0.974	Yes
atp4:250	0.903	nonsynonymous	CCG>TCG	Pro-to-Ser	5/3	0.375	NE	40/3	0.070	Yes
atp4:251	1.000	nonsynonymous	CCG>CTG	Pro-to-Leu	1/7	0.875	NE	0/43	1.000	Yes
atp4:395	0.999	nonsynonymous	TCA>TTA	Ser-to-Leu	0/16	1.000	NE	1/72	0.986	Yes
atp4:407	0.969	nonsynonymous	CCA>CTA	Pro-to-Leu	0/19	1.000	NE	2/73	0.973	Yes
atp6:37	0.999	nonsynonymous	CCA>TCA	Pro-to-Ser	3/9	0.750	NE	4/45	0.918	Yes
atp6:116	0.999	nonsynonymous	TCA>TTA	Ser-to-Leu	2/8	0.800	NE	0/40	1.000	Yes
atp6:167	0.999	nonsynonymous	CCG>CTG	Pro-to-Leu	2/12	0.857	NE	1/43	0.977	Yes
atp6:173	0.998	nonsynonymous	CCG>CTG	Pro-to-Leu	2/10	0.833	NE	2/38	0.950	Yes
atp6:224	0.993	nonsynonymous	TCC>TTC	Ser-to-Phe	0/13	1.000	NE	1/43	0.977	Yes
atp6:229	0.997	nonsynonymous	CGC>TGC	Arg-to-Cys	0/13	1.000	NE	1/41	0.976	Yes
atp6:236	0.983	nonsynonymous	TCG>TTG	Ser-to-Leu	0/13	1.000	NE	1/46	0.979	Yes
atp6:254	0.996	nonsynonymous	TCG>TTG	Ser-to-Leu	0/15	1.000	NE	1/48	0.980	Yes
atp6:262	0.999	nonsynonymous	CGT>TGT	Arg-to-Cys	2/13	0.867	NE	1/49	0.980	Yes
atp6:270	0.971	synonymous	CTC>CTT	Leu-to-Leu	2/12	0.857	NE	27/24	0.471	Yes
atp6:401	0.999	nonsynonymous	TCA>TTA	Ser-to-Leu	0/13	1.000	NE	2/41	0.953	Yes
atp6:460	0.999	nonsynonymous	CCT>TCT	Pro-to-Ser	0/18	1.000	NE	0/32	1.000	Yes

Site ID	Score	Codon effect	Codon change	AA change	L1 C/T	L1 T fraction	L1 support	L2 C/T	L2 T fraction	L2 support
atp6:463	1.000	nonsynonymous	CAT>TAT	His-to-Tyr	0/18	1.000	NE	0/32	1.000	Yes
atp6:485	0.999	nonsynonymous	TCA>TTA	Ser-to-Leu	0/18	1.000	NE	0/31	1.000	Yes
atp6:527	1.000	nonsynonymous	TCA>TTA	Ser-to-Leu	0/15	1.000	NE	0/36	1.000	Yes
atp6:548	1.000	nonsynonymous	TCC>TTC	Ser-to-Phe	0/21	1.000	Yes	0/33	1.000	Yes
atp6:635	0.999	nonsynonymous	CCG>CTG	Pro-to-Leu	3/22	0.880	Yes	2/29	0.935	Yes
atp6:656	0.998	nonsynonymous	TCA>TTA	Ser-to-Leu	3/21	0.875	Yes	2/29	0.935	Yes
atp6:664	0.992	nonsynonymous	CAT>TAT	His-to-Tyr	3/20	0.870	Yes	2/27	0.931	Yes
atp6:671	0.996	nonsynonymous	TCT>TTT	Ser-to-Phe	0/20	1.000	Yes	0/28	1.000	Yes
atp6:707	1.000	nonsynonymous	ACA>ATA	Thr-to-Ile	2/17	0.895	NE	15/13	0.464	Yes
atp6:718	0.925	stop_gain	CAA>TAA	Gln-to-Stop	1/13	0.929	NE	1/22	0.957	Yes
atp6:837	0.561	synonymous	CTC>CTT	Leu-to-Leu	0/0	NA	NE	0/0	NA	NE
atp6:846	0.857	synonymous	TTC>TTT	Phe-to-Phe	0/0	NA	NE	0/0	NA	NE
atp6:866	0.558	nonsynonymous	CCC>CTC	Pro-to-Leu	0/0	NA	NE	0/0	NA	NE
atp8:21	0.521	synonymous	TTC>TTT	Phe-to-Phe	18/0	0.000	NE	23/0	0.000	No
atp8:30	0.937	synonymous	TTC>TTT	Phe-to-Phe	19/0	0.000	NE	22/1	0.043	No
atp8:47	0.996	nonsynonymous	TCA>TTA	Ser-to-Leu	0/19	1.000	NE	2/22	0.917	Yes
atp8:58	0.998	nonsynonymous	CTC>TTC	Leu-to-Phe	0/19	1.000	NE	2/24	0.923	Yes
atp8:76	0.997	nonsynonymous	CCC>TCC	Pro-to-Ser	0/18	1.000	NE	1/23	0.958	Yes
atp8:77	0.794	nonsynonymous	CCC>CTC	Pro-to-Leu	0/18	1.000	NE	2/22	0.917	Yes
atp8:240	0.982	synonymous	TCC>TCT	Ser-to-Ser	20/0	0.000	No	32/0	0.000	No
atp8:432	0.583	synonymous	GAC>GAT	Asp-to-Asp	8/0	0.000	NE	37/0	0.000	No
atp8:452	1.000	nonsynonymous	CCA>CTA	Pro-to-Leu	0/7	1.000	NE	1/30	0.968	Yes
atp9:20	1.000	nonsynonymous	TCA>TTA	Ser-to-Leu	1/73	0.986	Yes	2/129	0.985	Yes
atp9:50	0.996	nonsynonymous	TCA>TTA	Ser-to-Leu	0/113	1.000	Yes	2/167	0.988	Yes
atp9:81	0.996	synonymous	GTC>GTT	Val-to-Val	9/123	0.932	Yes	2/189	0.990	Yes
atp9:90	0.989	synonymous	TCC>TCT	Ser-to-Ser	134/0	0.000	No	205/0	0.000	No
atp9:92	0.999	nonsynonymous	TCG>TTG	Ser-to-Leu	4/130	0.970	Yes	2/207	0.990	Yes
atp9:134	1.000	nonsynonymous	TCA>TTA	Ser-to-Leu	1/119	0.992	Yes	7/193	0.965	Yes
atp9:182	1.000	nonsynonymous	TCG>TTG	Ser-to-Leu	2/106	0.981	Yes	8/171	0.955	Yes

Site ID	Score	Codon effect	Codon change	AA change	L1 C/T	L1 T fraction	L1 support	L2 C/T	L2 T fraction	L2 support
atp9:190	0.648	nonsynonymous	CCA>TCA	Pro-to-Ser	95/0	0.000	No	150/0	0.000	No
atp9:191	1.000	nonsynonymous	CCA>CTA	Pro-to-Leu	4/85	0.955	Yes	10/146	0.936	Yes
atp9:205	0.987	synonymous	CTG>TTG	Leu-to-Leu	1/72	0.986	Yes	9/136	0.938	Yes
atp9:212	0.998	nonsynonymous	TCA>TTA	Ser-to-Leu	3/62	0.954	Yes	8/127	0.941	Yes
atp9:215	0.996	nonsynonymous	TCC>TTC	Ser-to-Phe	1/64	0.985	Yes	8/128	0.941	Yes
atp9:223	0.969	stop_gain	CGA>TGA	Arg-to-Stop	3/2	0.400	NE	9/8	0.471	NE
ccmB:16	0.885	nonsynonymous	CTT>TTT	Leu-to-Phe	4/0	0.000	NE	1/0	0.000	NE
ccmB:28	0.988	nonsynonymous	CAT>TAT	His-to-Tyr	2/2	0.500	NE	0/1	1.000	NE
ccmB:43	0.995	nonsynonymous	CCC>TCC	Pro-to-Ser	2/2	0.500	NE	0/1	1.000	NE
ccmB:70	0.858	nonsynonymous	CCA>TCA	Pro-to-Ser	6/0	0.000	NE	2/0	0.000	NE
ccmB:71	0.992	nonsynonymous	CCA>CTA	Pro-to-Leu	2/3	0.600	NE	1/1	0.500	NE
ccmB:80	0.969	nonsynonymous	TCG>TTG	Ser-to-Leu	2/3	0.600	NE	1/1	0.500	NE
ccmB:87	0.963	synonymous	ATC>ATT	Ile-to-Ile	2/3	0.600	NE	1/1	0.500	NE
ccmB:128	0.998	nonsynonymous	TCA>TTA	Ser-to-Leu	1/6	0.857	NE	0/2	1.000	NE
ccmB:137	0.988	nonsynonymous	TCC>TTC	Ser-to-Phe	2/4	0.667	NE	0/1	1.000	NE
ccmB:148	0.989	nonsynonymous	CCG>TCG	Pro-to-Ser	1/3	0.750	NE	1/1	0.500	NE
ccmB:149	0.996	nonsynonymous	CCG>CTG	Pro-to-Leu	1/3	0.750	NE	1/1	0.500	NE
ccmB:154	0.978	nonsynonymous	CGG>TGG	Arg-to-Trp	1/3	0.750	NE	1/1	0.500	NE
ccmB:160	0.992	nonsynonymous	CCT>TCT	Pro-to-Ser	1/3	0.750	NE	0/1	1.000	NE
ccmB:164	0.990	nonsynonymous	CCG>CTG	Pro-to-Leu	1/3	0.750	NE	1/1	0.500	NE
ccmB:172	0.990	nonsynonymous	CCT>TCT	Pro-to-Ser	1/5	0.833	NE	0/1	1.000	NE
ccmB:179	0.970	nonsynonymous	CCT>CTT	Pro-to-Leu	1/5	0.833	NE	0/1	1.000	NE
ccmB:183	0.564	synonymous	TCC>TCT	Ser-to-Ser	1/4	0.800	NE	0/0	NA	NE
ccmB:193	0.984	nonsynonymous	CCT>TCT	Pro-to-Ser	0/4	1.000	NE	1/0	0.000	NE
ccmB:194	0.999	nonsynonymous	CCT>CTT	Pro-to-Leu	0/4	1.000	NE	1/0	0.000	NE
ccmB:286	0.999	nonsynonymous	CGG>TGG	Arg-to-Trp	0/1	1.000	NE	2/0	0.000	NE
ccmB:304	0.996	nonsynonymous	CGT>TGT	Arg-to-Cys	0/1	1.000	NE	2/0	0.000	NE
ccmB:313	0.999	nonsynonymous	CGT>TGT	Arg-to-Cys	0/2	1.000	NE	2/0	0.000	NE
ccmB:337	0.699	nonsynonymous	CCG>TCG	Pro-to-Ser	2/0	0.000	NE	1/0	0.000	NE

Site ID	Score	Codon effect	Codon change	AA change	L1 C/T	L1 T fraction	L1 support	L2 C/T	L2 T fraction	L2 support
ccmB:338	0.997	nonsynonymous	CCG>CTG	Pro-to-Leu	0/2	1.000	NE	0/1	1.000	NE
ccmB:367	0.653	nonsynonymous	CGG>TGG	Arg-to-Trp	0/5	1.000	NE	2/0	0.000	NE
ccmB:379	0.697	synonymous	CTA>TTA	Leu-to-Leu	0/6	1.000	NE	2/0	0.000	NE
ccmB:424	0.994	nonsynonymous	CGT>TGT	Arg-to-Cys	3/10	0.769	NE	3/1	0.250	NE
ccmB:428	0.996	nonsynonymous	TCG>TTG	Ser-to-Leu	3/10	0.769	NE	3/1	0.250	NE
ccmB:467	0.975	nonsynonymous	TCG>TTG	Ser-to-Leu	3/10	0.769	NE	1/2	0.667	NE
ccmB:475	0.997	nonsynonymous	CCA>TCA	Pro-to-Ser	3/10	0.769	NE	3/0	0.000	NE
ccmB:476	0.996	nonsynonymous	CCA>CTA	Pro-to-Leu	4/9	0.692	NE	3/1	0.250	NE
ccmB:485	0.985	nonsynonymous	TCA>TTA	Ser-to-Leu	6/8	0.571	NE	3/1	0.250	NE
ccmB:494	0.987	nonsynonymous	TCA>TTA	Ser-to-Leu	8/7	0.467	NE	4/0	0.000	NE
ccmB:502	0.972	nonsynonymous	CCA>TCA	Pro-to-Ser	14/1	0.067	NE	4/0	0.000	NE
ccmB:503	0.991	nonsynonymous	CCA>CTA	Pro-to-Leu	7/9	0.562	NE	3/1	0.250	NE
ccmB:512	0.981	nonsynonymous	TCT>TTT	Ser-to-Phe	5/6	0.545	NE	3/1	0.250	NE
ccmB:514	0.995	nonsynonymous	CGT>TGT	Arg-to-Cys	6/5	0.455	NE	0/1	1.000	NE
ccmB:551	0.994	nonsynonymous	TCA>TTA	Ser-to-Leu	3/6	0.667	NE	1/1	0.500	NE
ccmB:554	0.969	nonsynonymous	TCG>TTG	Ser-to-Leu	4/5	0.556	NE	0/2	1.000	NE
ccmB:566	0.999	nonsynonymous	TCC>TTC	Ser-to-Phe	5/6	0.545	NE	0/2	1.000	NE
ccmB:569	0.973	nonsynonymous	TCT>TTT	Ser-to-Phe	4/4	0.500	NE	1/1	0.500	NE
ccmB:596	0.995	nonsynonymous	TCG>TTG	Ser-to-Leu	5/4	0.444	NE	0/2	1.000	NE
ccmB:611	0.835	nonsynonymous	TCA>TTA	Ser-to-Leu	0/4	1.000	NE	0/1	1.000	NE
ccmC:76	1.000	nonsynonymous	CGG>TGG	Arg-to-Trp	0/4	1.000	NE	0/6	1.000	NE
ccmC:84	0.532	synonymous	TTC>TTT	Phe-to-Phe	5/0	0.000	NE	6/1	0.143	NE
ccmC:103	0.999	nonsynonymous	CAT>TAT	His-to-Tyr	7/5	0.417	NE	0/9	1.000	NE
ccmC:115	0.991	nonsynonymous	CGG>TGG	Arg-to-Trp	11/4	0.267	NE	0/9	1.000	NE
ccmC:133	0.987	nonsynonymous	CTT>TTT	Leu-to-Phe	8/6	0.429	NE	0/7	1.000	NE
ccmC:161	1.000	nonsynonymous	CCG>CTG	Pro-to-Leu	7/4	0.364	NE	0/8	1.000	NE
ccmC:179	0.999	nonsynonymous	GCG>GTG	Ala-to-Val	4/6	0.600	NE	0/8	1.000	NE
ccmC:184	0.994	nonsynonymous	CGG>TGG	Arg-to-Trp	5/4	0.444	NE	0/7	1.000	NE
ccmC:281	0.976	nonsynonymous	ACA>ATA	Thr-to-Ile	0/8	1.000	NE	0/10	1.000	NE

Site ID	Score	Codon effect	Codon change	AA change	L1 C/T	L1 T fraction	L1 support	L2 C/T	L2 T fraction	L2 support
ccmC:299	0.993	nonsynonymous	TCT>TTT	Ser-to-Phe	0/8	1.000	NE	0/8	1.000	NE
ccmC:331	0.999	nonsynonymous	CGG>TGG	Arg-to-Trp	1/6	0.857	NE	11/9	0.450	Yes
ccmC:395	0.996	nonsynonymous	TCG>TTG	Ser-to-Leu	2/7	0.778	NE	6/25	0.806	Yes
ccmC:399	0.994	synonymous	TTC>TTT	Phe-to-Phe	1/8	0.889	NE	5/23	0.821	Yes
ccmC:400	0.999	nonsynonymous	CTT>TTT	Leu-to-Phe	1/8	0.889	NE	4/24	0.857	Yes
ccmC:418	0.809	synonymous	CTG>TTG	Leu-to-Leu	9/3	0.250	NE	28/4	0.125	Yes
ccmC:421	0.999	nonsynonymous	CGT>TGT	Arg-to-Cys	0/12	1.000	NE	6/28	0.824	Yes
ccmC:436	0.998	nonsynonymous	CCT>TCT	Pro-to-Ser	3/10	0.769	NE	6/28	0.824	Yes
ccmC:446	0.999	nonsynonymous	CCG>CTG	Pro-to-Leu	1/13	0.929	NE	2/34	0.944	Yes
ccmC:451	0.999	nonsynonymous	CCT>TCT	Pro-to-Ser	1/12	0.923	NE	5/28	0.848	Yes
ccmC:458	0.979	nonsynonymous	TCA>TTA	Ser-to-Leu	1/14	0.933	NE	3/32	0.914	Yes
ccmC:463	0.999	nonsynonymous	CGT>TGT	Arg-to-Cys	3/12	0.800	NE	6/29	0.829	Yes
ccmC:467	0.992	nonsynonymous	GCT>GTT	Ala-to-Val	4/10	0.714	NE	6/31	0.838	Yes
ccmC:473	0.998	nonsynonymous	CCG>CTG	Pro-to-Leu	2/12	0.857	NE	5/30	0.857	Yes
ccmC:497	0.999	nonsynonymous	TCT>TTT	Ser-to-Phe	3/9	0.750	NE	5/30	0.857	Yes
ccmC:521	0.999	nonsynonymous	TCG>TTG	Ser-to-Leu	2/11	0.846	NE	2/31	0.939	Yes
ccmC:548	0.943	nonsynonymous	TCT>TTT	Ser-to-Phe	4/9	0.692	NE	6/14	0.700	Yes
ccmC:568	0.996	nonsynonymous	CCT>TCT	Pro-to-Ser	3/9	0.750	NE	0/23	1.000	Yes
ccmC:575	0.999	nonsynonymous	CCC>CTC	Pro-to-Leu	2/8	0.800	NE	0/22	1.000	Yes
ccmC:605	1.000	nonsynonymous	TCC>TTC	Ser-to-Phe	1/10	0.909	NE	0/22	1.000	Yes
ccmC:606	0.731	synonymous	TCC>TCT	Ser-to-Ser	3/7	0.700	NE	15/11	0.423	Yes
ccmC:608	1.000	nonsynonymous	CCC>CTC	Pro-to-Leu	0/11	1.000	NE	0/23	1.000	Yes
ccmC:614	0.985	nonsynonymous	TCA>TTA	Ser-to-Leu	2/10	0.833	NE	2/20	0.909	Yes
ccmC:619	0.961	nonsynonymous	CGT>TGT	Arg-to-Cys	1/9	0.900	NE	2/20	0.909	Yes
ccmC:624	0.993	synonymous	ATC>ATT	Ile-to-Ile	11/1	0.083	NE	20/3	0.130	Yes
ccmC:650	0.997	nonsynonymous	CCT>CTT	Pro-to-Leu	0/11	1.000	NE	4/17	0.810	Yes
ccmC:656	0.990	nonsynonymous	CCA>CTA	Pro-to-Leu	2/10	0.833	NE	4/19	0.826	Yes
ccmC:673	0.938	nonsynonymous	CCT>TCT	Pro-to-Ser	0/6	1.000	NE	4/17	0.810	Yes
ccmC:744	0.555	synonymous	ATC>ATT	Ile-to-Ile	0/0	NA	NE	4/0	0.000	NE

Site ID	Score	Codon effect	Codon change	AA change	L1 C/T	L1 T fraction	L1 support	L2 C/T	L2 T fraction	L2 support
ccmFC:38	0.998	nonsynonymous	TCC>TTC	Ser-to-Phe	2/0	0.000	NE	0/7	1.000	NE
ccmFC:39	0.809	synonymous	TCC>TCT	Ser-to-Ser	2/0	0.000	NE	7/0	0.000	NE
ccmFC:50	1.000	nonsynonymous	CCT>CTT	Pro-to-Leu	2/0	0.000	NE	0/8	1.000	NE
ccmFC:52	0.984	nonsynonymous	CGT>TGT	Arg-to-Cys	2/0	0.000	NE	0/8	1.000	NE
ccmFC:103	0.999	nonsynonymous	CCC>TCC	Pro-to-Ser	2/0	0.000	NE	0/7	1.000	NE
ccmFC:122	0.999	nonsynonymous	TCC>TTC	Ser-to-Phe	2/0	0.000	NE	0/7	1.000	NE
ccmFC:123	0.702	synonymous	TCC>TCT	Ser-to-Ser	1/0	0.000	NE	6/1	0.143	NE
ccmFC:146	0.997	nonsynonymous	CCT>CTT	Pro-to-Leu	0/0	NA	NE	0/7	1.000	NE
ccmFC:151	0.988	nonsynonymous	CCG>TCG	Pro-to-Ser	0/0	NA	NE	0/6	1.000	NE
ccmFC:160	0.638	nonsynonymous	CCT>TCT	Pro-to-Ser	0/0	NA	NE	3/0	0.000	NE
ccmFC:305	0.987	nonsynonymous	TCA>TTA	Ser-to-Leu	3/1	0.250	NE	2/1	0.333	NE
ccmFC:333	0.940	synonymous	TTC>TTT	Phe-to-Phe	4/0	0.000	NE	6/0	0.000	NE
ccmFC:334	0.941	nonsynonymous	CTT>TTT	Leu-to-Phe	3/1	0.250	NE	5/1	0.167	NE
ccmFC:360	0.522	synonymous	TTC>TTT	Phe-to-Phe	3/0	0.000	NE	6/0	0.000	NE
ccmFC:391	0.999	nonsynonymous	CGT>TGT	Arg-to-Cys	2/2	0.500	NE	2/6	0.750	NE
ccmFC:406	0.996	nonsynonymous	CGT>TGT	Arg-to-Cys	3/2	0.400	NE	2/11	0.846	NE
ccmFC:731	0.894	nonsynonymous	TCA>TTA	Ser-to-Leu	6/0	0.000	NE	10/0	0.000	NE
ccmFC:1145	0.998	nonsynonymous	CCA>CTA	Pro-to-Leu	0/2	1.000	NE	1/3	0.750	NE
ccmFC:1166	1.000	nonsynonymous	TCG>TTG	Ser-to-Leu	1/2	0.667	NE	1/3	0.750	NE
ccmFC:1274	0.993	nonsynonymous	TCG>TTG	Ser-to-Leu	1/0	0.000	NE	4/3	0.429	NE
ccmFC:1321	0.991	stop_gain	CGA>TGA	Arg-to-Stop	0/0	NA	NE	0/0	NA	NE
ccmFN:143	0.996	nonsynonymous	TCG>TTG	Ser-to-Leu	1/5	0.833	NE	0/18	1.000	NE
ccmFN:157	0.994	nonsynonymous	CCT>TCT	Pro-to-Ser	1/5	0.833	NE	0/21	1.000	Yes
ccmFN:254	0.999	nonsynonymous	TCA>TTA	Ser-to-Leu	0/2	1.000	NE	1/14	0.933	NE
ccmFN:262	0.999	nonsynonymous	CGG>TGG	Arg-to-Trp	0/1	1.000	NE	1/13	0.929	NE
ccmFN:289	0.978	nonsynonymous	CTT>TTT	Leu-to-Phe	0/1	1.000	NE	1/11	0.917	NE
ccmFN:326	0.599	nonsynonymous	TCA>TTA	Ser-to-Leu	5/0	0.000	NE	9/0	0.000	NE
ccmFN:378	0.979	synonymous	TTC>TTT	Phe-to-Phe	4/2	0.333	NE	5/6	0.545	NE
ccmFN:613	0.713	nonsynonymous	CAT>TAT	His-to-Tyr	1/0	0.000	NE	13/0	0.000	NE

Site ID	Score	Codon effect	Codon change	AA change	L1 C/T	L1 T fraction	L1 support	L2 C/T	L2 T fraction	L2 support
ccmFN:732	0.555	synonymous	TCC>TCT	Ser-to-Ser	2/0	0.000	NE	13/0	0.000	NE
ccmFN:765	0.569	synonymous	ACC>ACT	Thr-to-Thr	1/1	0.500	NE	6/3	0.333	NE
ccmFN:999	0.936	synonymous	CTC>CTT	Leu-to-Leu	8/0	0.000	NE	12/0	0.000	NE
ccmFN:1193	0.840	nonsynonymous	TCG>TTG	Ser-to-Leu	3/0	0.000	NE	14/0	0.000	NE
ccmFN:1276	0.992	nonsynonymous	CGG>TGG	Arg-to-Trp	0/4	1.000	NE	3/9	0.750	NE
ccmFN:1304	1.000	nonsynonymous	CCA>CTA	Pro-to-Leu	0/4	1.000	NE	0/12	1.000	NE
ccmFN:1321	1.000	nonsynonymous	CAT>TAT	His-to-Tyr	0/2	1.000	NE	1/12	0.923	NE
ccmFN:1336	0.999	nonsynonymous	CGG>TGG	Arg-to-Trp	0/2	1.000	NE	1/13	0.929	NE
ccmFN:1354	0.999	nonsynonymous	CGG>TGG	Arg-to-Trp	0/1	1.000	NE	5/9	0.643	NE
ccmFN:1387	0.999	nonsynonymous	CGG>TGG	Arg-to-Trp	0/5	1.000	NE	1/18	0.947	NE
ccmFN:1405	0.999	nonsynonymous	CGT>TGT	Arg-to-Cys	0/5	1.000	NE	2/18	0.900	Yes
ccmFN:1429	0.649	nonsynonymous	CTT>TTT	Leu-to-Phe	4/0	0.000	NE	15/0	0.000	NE
ccmFN:1448	0.998	nonsynonymous	TCG>TTG	Ser-to-Leu	0/4	1.000	NE	0/17	1.000	NE
ccmFN:1450	0.997	nonsynonymous	CTT>TTT	Leu-to-Phe	0/4	1.000	NE	0/17	1.000	NE
ccmFN:1468	0.995	nonsynonymous	CTT>TTT	Leu-to-Phe	0/4	1.000	NE	1/19	0.950	Yes
ccmFN:1472	0.994	nonsynonymous	CCA>CTA	Pro-to-Leu	0/4	1.000	NE	1/19	0.950	Yes
ccmFN:1484	1.000	nonsynonymous	TCA>TTA	Ser-to-Leu	0/4	1.000	NE	1/19	0.950	Yes
ccmFN:1493	0.998	nonsynonymous	TCT>TTT	Ser-to-Phe	0/4	1.000	NE	1/19	0.950	Yes
ccmFN:1519	0.999	nonsynonymous	CCT>TCT	Pro-to-Ser	0/4	1.000	NE	1/18	0.947	NE
cob:414	0.699	synonymous	TAC>TAT	Tyr-to-Tyr	154/0	0.000	No	41/0	0.000	No
cob:1089	0.628	synonymous	TTC>TTT	Phe-to-Phe	135/0	0.000	No	21/0	0.000	No
cob:1098	0.549	synonymous	TTC>TTT	Phe-to-Phe	137/3	0.021	No	22/0	0.000	No
cob:1160	0.808	nonsynonymous	ACG>ATG	Thr-to-Met	71/33	0.317	Yes	10/0	0.000	NE
cox1:11	1.000	nonsynonymous	CCG>CTG	Pro-to-Leu	1/13	0.929	NE	1/49	0.980	Yes
cox1:15	0.954	synonymous	GTC>GTT	Val-to-Val	15/0	0.000	NE	55/0	0.000	No
cox1:242	0.998	nonsynonymous	TCT>TTT	Ser-to-Phe	42/33	0.440	Yes	5/74	0.937	Yes
cox1:254	1.000	nonsynonymous	TCT>TTT	Ser-to-Phe	41/33	0.446	Yes	9/80	0.899	Yes
cox1:1186	0.998	nonsynonymous	CAC>TAC	His-to-Tyr	1/14	0.933	NE	0/57	1.000	Yes
cox1:1405	1.000	nonsynonymous	CGT>TGT	Arg-to-Cys	0/12	1.000	NE	1/67	0.985	Yes

Site ID	Score	Codon effect	Codon change	AA change	L1 C/T	L1 T fraction	L1 support	L2 C/T	L2 T fraction	L2 support
cox1:1433	0.990	nonsynonymous	TCA>TTA	Ser-to-Leu	2/16	0.889	NE	1/66	0.985	Yes
cox1:1446	0.986	synonymous	AAC>AAT	Asn-to-Asn	19/0	0.000	NE	63/0	0.000	No
cox1:1489	0.996	nonsynonymous	CCA>TCA	Pro-to-Ser	0/18	1.000	NE	1/56	0.982	Yes
cox1:1499	0.998	nonsynonymous	TCG>TTG	Ser-to-Leu	0/15	1.000	NE	1/55	0.982	Yes
cox2:30	0.836	synonymous	TTC>TTT	Phe-to-Phe	54/0	0.000	No	220/1	0.005	No
cox2:31	0.852	nonsynonymous	CTC>TTC	Leu-to-Phe	49/3	0.058	Yes	216/6	0.027	No
cox2:77	1.000	nonsynonymous	TCT>TTT	Ser-to-Phe	34/23	0.404	Yes	5/203	0.976	Yes
cox2:144	0.894	synonymous	CTC>CTT	Leu-to-Leu	25/0	0.000	No	79/0	0.000	No
cox2:167	0.997	nonsynonymous	TCA>TTA	Ser-to-Leu	9/18	0.667	Yes	0/79	1.000	Yes
cox2:169	0.999	nonsynonymous	CGG>TGG	Arg-to-Trp	9/18	0.667	Yes	1/80	0.988	Yes
cox2:259	0.994	nonsynonymous	CGG>TGG	Arg-to-Trp	9/9	0.500	NE	2/86	0.977	Yes
cox2:284	0.993	nonsynonymous	CCG>CTG	Pro-to-Leu	3/7	0.700	NE	2/84	0.977	Yes
cox2:385	0.999	nonsynonymous	CGG>TGG	Arg-to-Trp	0/11	1.000	NE	4/71	0.947	Yes
cox2:449	0.999	nonsynonymous	ACG>ATG	Thr-to-Met	0/9	1.000	NE	4/74	0.949	Yes
cox2:466	0.823	nonsynonymous	CCA>TCA	Pro-to-Ser	8/1	0.111	NE	84/0	0.000	No
cox2:467	1.000	nonsynonymous	CCA>CTA	Pro-to-Leu	0/9	1.000	NE	5/76	0.938	Yes
cox2:482	1.000	nonsynonymous	TCA>TTA	Ser-to-Leu	0/13	1.000	NE	7/76	0.916	Yes
cox2:587	1.000	nonsynonymous	TCA>TTA	Ser-to-Leu	0/5	1.000	NE	1/75	0.987	Yes
cox2:629	0.907	nonsynonymous	ACC>ATC	Thr-to-Ile	3/0	0.000	NE	83/0	0.000	No
cox2:638	0.999	nonsynonymous	TCG>TTG	Ser-to-Leu	0/3	1.000	NE	8/77	0.906	Yes
cox2:704	1.000	nonsynonymous	ACG>ATG	Thr-to-Met	0/1	1.000	NE	1/68	0.986	Yes
cox2:727	0.999	nonsynonymous	CCT>TCT	Pro-to-Ser	0/2	1.000	NE	3/63	0.955	Yes
cox2:748	0.997	nonsynonymous	CGG>TGG	Arg-to-Trp	2/2	0.500	NE	0/61	1.000	Yes
cox2:781	0.831	stop_gain	CAA>TAA	Gln-to-Stop	4/0	0.000	NE	47/0	0.000	No
matR:32	0.886	nonsynonymous	TCC>TTC	Ser-to-Phe	2/5	0.714	NE	1/14	0.933	NE
matR:43	0.689	nonsynonymous	CCC>TCC	Pro-to-Ser	4/3	0.429	NE	2/13	0.867	NE
matR:236	0.713	nonsynonymous	TCC>TTC	Ser-to-Phe	24/3	0.111	Yes	15/0	0.000	NE
matR:254	0.658	nonsynonymous	TCC>TTC	Ser-to-Phe	27/0	0.000	No	14/0	0.000	NE
matR:326	0.995	nonsynonymous	CCA>CTA	Pro-to-Leu	0/24	1.000	Yes	0/11	1.000	NE

Site ID	Score	Codon effect	Codon change	AA change	L1 C/T	L1 T fraction	L1 support	L2 C/T	L2 T fraction	L2 support
matR:413	0.973	nonsynonymous	TCG>TTG	Ser-to-Leu	0/18	1.000	NE	0/12	1.000	NE
matR:923	1.000	nonsynonymous	TCA>TTA	Ser-to-Leu	0/22	1.000	Yes	3/14	0.824	NE
matR:1065	0.597	synonymous	CTC>CTT	Leu-to-Leu	20/0	0.000	No	20/0	0.000	No
matR:1545	0.757	synonymous	CCC>CCT	Pro-to-Pro	0/14	1.000	NE	3/8	0.727	NE
matR:1679	0.924	nonsynonymous	TCC>TTC	Ser-to-Phe	2/24	0.923	Yes	5/12	0.706	NE
matR:1700	0.999	nonsynonymous	CCT>CTT	Pro-to-Leu	2/21	0.913	Yes	5/13	0.722	NE
matR:1720	1.000	nonsynonymous	CGC>TGC	Arg-to-Cys	2/20	0.909	Yes	4/12	0.750	NE
matR:1734	0.996	synonymous	TAC>TAT	Tyr-to-Tyr	12/2	0.143	NE	13/2	0.133	NE
matR:1756	0.995	nonsynonymous	CAC>TAC	His-to-Tyr	2/10	0.833	NE	4/11	0.733	NE
matR:1787	0.997	nonsynonymous	CCG>CTG	Pro-to-Leu	0/12	1.000	NE	1/7	0.875	NE
matR:1826	0.998	nonsynonymous	CCA>CTA	Pro-to-Leu	0/15	1.000	NE	7/10	0.588	NE
matR:1844	0.998	nonsynonymous	TCA>TTA	Ser-to-Leu	0/13	1.000	NE	4/13	0.765	NE
mttB:16	0.958	nonsynonymous	CAT>TAT	His-to-Tyr	0/0	NA	NE	0/2	1.000	NE
mttB:21	0.671	synonymous	TTC>TTT	Phe-to-Phe	0/0	NA	NE	2/0	0.000	NE
mttB:25	0.981	nonsynonymous	CCG>TCG	Pro-to-Ser	0/0	NA	NE	0/0	NA	NE
mttB:26	0.950	nonsynonymous	CCG>CTG	Pro-to-Leu	0/0	NA	NE	0/1	1.000	NE
mttB:37	0.662	synonymous	CTA>TTA	Leu-to-Leu	0/0	NA	NE	1/0	0.000	NE
mttB:59	0.938	nonsynonymous	TCG>TTG	Ser-to-Leu	0/0	NA	NE	1/0	0.000	NE
mttB:64	0.976	nonsynonymous	CGG>TGG	Arg-to-Trp	0/0	NA	NE	0/1	1.000	NE
mttB:100	0.999	nonsynonymous	CGT>TGT	Arg-to-Cys	0/0	NA	NE	0/5	1.000	NE
mttB:111	0.862	synonymous	TTC>TTT	Phe-to-Phe	0/0	NA	NE	5/1	0.167	NE
mttB:112	0.998	nonsynonymous	CCG>TCG	Pro-to-Ser	0/0	NA	NE	3/2	0.400	NE
mttB:128	0.996	nonsynonymous	TCT>TTT	Ser-to-Phe	0/0	NA	NE	1/5	0.833	NE
mttB:130	0.700	nonsynonymous	CCA>TCA	Pro-to-Ser	0/0	NA	NE	6/0	0.000	NE
mttB:131	0.985	nonsynonymous	CCA>CTA	Pro-to-Leu	0/0	NA	NE	3/3	0.500	NE
mttB:178	0.992	nonsynonymous	CGT>TGT	Arg-to-Cys	0/0	NA	NE	1/5	0.833	NE
mttB:188	1.000	nonsynonymous	TCA>TTA	Ser-to-Leu	0/0	NA	NE	0/4	1.000	NE
mttB:200	1.000	nonsynonymous	TCC>TTC	Ser-to-Phe	0/0	NA	NE	0/6	1.000	NE
mttB:202	0.996	nonsynonymous	CCG>TCG	Pro-to-Ser	0/0	NA	NE	0/6	1.000	NE

Site ID	Score	Codon effect	Codon change	AA change	L1 C/T	L1 T fraction	L1 support	L2 C/T	L2 T fraction	L2 support
mttB:236	0.755	nonsynonymous	TCT>TTT	Ser-to-Phe	0/0	NA	NE	4/0	0.000	NE
mttB:252	0.773	synonymous	CCC>CCT	Pro-to-Pro	0/0	NA	NE	1/3	0.750	NE
mttB:262	0.991	nonsynonymous	CAT>TAT	His-to-Tyr	0/0	NA	NE	1/4	0.800	NE
mttB:285	0.503	synonymous	ATC>ATT	Ile-to-Ile	0/0	NA	NE	6/0	0.000	NE
mttB:328	0.984	nonsynonymous	CTC>TTC	Leu-to-Phe	0/0	NA	NE	0/8	1.000	NE
mttB:331	0.983	nonsynonymous	CAT>TAT	His-to-Tyr	0/0	NA	NE	0/8	1.000	NE
mttB:344	0.933	nonsynonymous	TCT>TTT	Ser-to-Phe	0/0	NA	NE	7/0	0.000	NE
mttB:346	0.999	nonsynonymous	CGC>TGC	Arg-to-Cys	0/0	NA	NE	0/8	1.000	NE
mttB:351	0.767	synonymous	TTC>TTT	Phe-to-Phe	0/0	NA	NE	3/5	0.625	NE
mttB:353	0.867	nonsynonymous	TCC>TTC	Ser-to-Phe	0/0	NA	NE	0/8	1.000	NE
mttB:354	0.593	synonymous	TCC>TCT	Ser-to-Ser	0/0	NA	NE	9/0	0.000	NE
mttB:373	0.975	nonsynonymous	CTT>TTT	Leu-to-Phe	0/0	NA	NE	0/8	1.000	NE
mttB:379	0.996	nonsynonymous	CGG>TGG	Arg-to-Trp	0/0	NA	NE	0/7	1.000	NE
mttB:407	0.999	nonsynonymous	CCA>CTA	Pro-to-Leu	0/0	NA	NE	0/8	1.000	NE
mttB:437	0.884	nonsynonymous	TCG>TTG	Ser-to-Leu	0/0	NA	NE	0/7	1.000	NE
mttB:441	0.775	synonymous	CTC>CTT	Leu-to-Leu	0/0	NA	NE	6/0	0.000	NE
mttB:472	0.997	nonsynonymous	CAT>TAT	His-to-Tyr	0/0	NA	NE	0/4	1.000	NE
mttB:497	0.994	nonsynonymous	TCG>TTG	Ser-to-Leu	0/0	NA	NE	0/7	1.000	NE
mttB:505	0.995	nonsynonymous	CCA>TCA	Pro-to-Ser	0/1	1.000	NE	3/4	0.571	NE
mttB:541	0.990	nonsynonymous	CGT>TGT	Arg-to-Cys	0/1	1.000	NE	0/5	1.000	NE
mttB:548	0.996	nonsynonymous	CCA>CTA	Pro-to-Leu	0/1	1.000	NE	0/5	1.000	NE
mttB:554	0.989	nonsynonymous	CCA>CTA	Pro-to-Leu	0/1	1.000	NE	0/5	1.000	NE
mttB:578	0.996	nonsynonymous	TCC>TTC	Ser-to-Phe	0/1	1.000	NE	0/5	1.000	NE
mttB:610	0.997	nonsynonymous	CCG>TCG	Pro-to-Ser	0/1	1.000	NE	0/6	1.000	NE
mttB:616	0.971	nonsynonymous	CTC>TTC	Leu-to-Phe	0/1	1.000	NE	2/4	0.667	NE
mttB:660	0.813	synonymous	ATC>ATT	Ile-to-Ile	0/1	1.000	NE	4/0	0.000	NE
mttB:667	0.782	nonsynonymous	CGT>TGT	Arg-to-Cys	0/0	NA	NE	0/4	1.000	NE
mttB:672	0.978	synonymous	TTC>TTT	Phe-to-Phe	0/0	NA	NE	0/4	1.000	NE
mttB:683	0.977	nonsynonymous	TCG>TTG	Ser-to-Leu	0/0	NA	NE	0/5	1.000	NE

Site ID	Score	Codon effect	Codon change	AA change	L1 C/T	L1 T fraction	L1 support	L2 C/T	L2 T fraction	L2 support
mttB:704	1.000	nonsynonymous	TCT>TTT	Ser-to-Phe	0/0	NA	NE	3/2	0.400	NE
mttB:713	0.998	nonsynonymous	TCG>TTG	Ser-to-Leu	0/0	NA	NE	0/5	1.000	NE
nad1:215	1.000	nonsynonymous	TCC>TTC	Ser-to-Phe	6/8	0.571	NE	1/28	0.966	Yes
nad1:265	0.999	nonsynonymous	CGG>TGG	Arg-to-Trp	6/11	0.647	NE	1/22	0.957	Yes
nad1:307	0.995	nonsynonymous	CCG>TCG	Pro-to-Ser	8/9	0.529	NE	1/23	0.958	Yes
nad1:308	1.000	nonsynonymous	CCG>CTG	Pro-to-Leu	7/10	0.588	NE	1/23	0.958	Yes
nad1:376	0.999	nonsynonymous	CGG>TGG	Arg-to-Trp	2/3	0.600	NE	0/14	1.000	NE
nad1:635	0.996	nonsynonymous	TCA>TTA	Ser-to-Leu	0/4	1.000	NE	3/41	0.932	Yes
nad1:823	0.960	nonsynonymous	CTT>TTT	Leu-to-Phe	14/0	0.000	NE	42/0	0.000	No
nad1:898	0.999	nonsynonymous	CGG>TGG	Arg-to-Trp	1/9	0.900	NE	2/52	0.963	Yes
nad1:909	0.558	synonymous	TTC>TTT	Phe-to-Phe	6/5	0.455	NE	45/6	0.118	Yes
nad1:928	0.997	nonsynonymous	CGG>TGG	Arg-to-Trp	2/8	0.800	NE	3/49	0.942	Yes
nad2:26	0.981	nonsynonymous	TCC>TTC	Ser-to-Phe	1/5	0.833	NE	0/14	1.000	NE
nad2:223	0.997	nonsynonymous	CTT>TTT	Leu-to-Phe	4/8	0.667	NE	0/24	1.000	Yes
nad2:252	0.653	synonymous	TTC>TTT	Phe-to-Phe	26/0	0.000	No	20/0	0.000	No
nad2:308	0.997	nonsynonymous	TCT>TTT	Ser-to-Phe	23/11	0.324	Yes	7/17	0.708	Yes
nad2:311	0.999	nonsynonymous	TCC>TTC	Ser-to-Phe	22/13	0.371	Yes	7/18	0.720	Yes
nad2:341	0.999	nonsynonymous	TCC>TTC	Ser-to-Phe	11/15	0.577	Yes	1/22	0.957	Yes
nad2:356	0.949	nonsynonymous	CCA>CTA	Pro-to-Leu	13/13	0.500	Yes	0/22	1.000	Yes
nad2:361	0.998	nonsynonymous	CCT>TCT	Pro-to-Ser	12/13	0.520	Yes	1/18	0.947	NE
nad2:401	0.998	nonsynonymous	TCA>TTA	Ser-to-Leu	8/11	0.579	NE	0/21	1.000	Yes
nad2:428	0.999	nonsynonymous	CCT>CTT	Pro-to-Leu	7/12	0.632	NE	0/21	1.000	Yes
nad2:497	0.999	nonsynonymous	TCG>TTG	Ser-to-Leu	4/10	0.714	NE	0/19	1.000	NE
nad2:525	0.578	synonymous	TCC>TCT	Ser-to-Ser	15/0	0.000	NE	19/0	0.000	NE
nad2:609	0.851	synonymous	ACC>ACT	Thr-to-Thr	11/0	0.000	NE	34/0	0.000	No
nad2:662	0.999	nonsynonymous	TCT>TTT	Ser-to-Phe	2/6	0.750	NE	1/31	0.969	Yes
nad2:677	0.997	nonsynonymous	TCC>TTC	Ser-to-Phe	2/9	0.818	NE	2/28	0.933	Yes
nad2:788	1.000	nonsynonymous	TCT>TTT	Ser-to-Phe	0/8	1.000	NE	2/20	0.909	Yes
nad2:800	0.986	nonsynonymous	TCA>TTA	Ser-to-Leu	2/9	0.818	NE	0/21	1.000	Yes

Site ID	Score	Codon effect	Codon change	AA change	L1 C/T	L1 T fraction	L1 support	L2 C/T	L2 T fraction	L2 support
nad2:809	0.998	nonsynonymous	TCT>TTT	Ser-to-Phe	2/8	0.800	NE	0/21	1.000	Yes
nad2:855	0.538	synonymous	TTC>TTT	Phe-to-Phe	11/0	0.000	NE	19/0	0.000	NE
nad2:958	0.999	nonsynonymous	CGT>TGT	Arg-to-Cys	0/8	1.000	NE	2/24	0.923	Yes
nad2:962	1.000	nonsynonymous	ACA>ATA	Thr-to-Ile	2/8	0.800	NE	6/22	0.786	Yes
nad2:1028	0.999	nonsynonymous	TCA>TTA	Ser-to-Leu	0/11	1.000	NE	3/28	0.903	Yes
nad2:1058	0.997	nonsynonymous	TCA>TTA	Ser-to-Leu	0/11	1.000	NE	4/34	0.895	Yes
nad2:1127	0.999	nonsynonymous	TCG>TTG	Ser-to-Leu	0/6	1.000	NE	2/27	0.931	Yes
nad2:1246	0.995	nonsynonymous	CCA>TCA	Pro-to-Ser	0/5	1.000	NE	0/20	1.000	Yes
nad2:1247	0.683	nonsynonymous	CCA>CTA	Pro-to-Leu	5/0	0.000	NE	21/0	0.000	No
nad2:1276	1.000	nonsynonymous	CGT>TGT	Arg-to-Cys	0/7	1.000	NE	0/17	1.000	NE
nad2:1400	0.964	nonsynonymous	TCA>TTA	Ser-to-Leu	0/11	1.000	NE	3/18	0.857	Yes
nad2:1408	0.976	nonsynonymous	CCA>TCA	Pro-to-Ser	1/9	0.900	NE	4/18	0.818	Yes
nad2:1409	0.991	nonsynonymous	CCA>CTA	Pro-to-Leu	0/9	1.000	NE	4/17	0.810	Yes
nad2:1416	0.976	synonymous	CCC>CCT	Pro-to-Pro	1/8	0.889	NE	5/16	0.762	Yes
nad2:1457	0.997	nonsynonymous	TCA>TTA	Ser-to-Leu	0/6	1.000	NE	0/19	1.000	NE
nad4:30	0.971	synonymous	TTC>TTT	Phe-to-Phe	3/0	0.000	NE	7/0	0.000	NE
nad4:57	0.658	synonymous	CCC>CCT	Pro-to-Pro	2/1	0.333	NE	10/1	0.091	NE
nad4:156	0.592	synonymous	TCC>TCT	Ser-to-Ser	4/0	0.000	NE	25/0	0.000	No
nad4:261	0.650	synonymous	GAC>GAT	Asp-to-Asp	1/0	0.000	NE	20/0	0.000	No
nad4:819	0.842	synonymous	CCC>CCT	Pro-to-Pro	4/0	0.000	NE	19/0	0.000	NE
nad4:837	0.695	synonymous	TTC>TTT	Phe-to-Phe	4/0	0.000	NE	17/0	0.000	NE
nad4:1405	0.999	nonsynonymous	CGG>TGG	Arg-to-Trp	2/2	0.500	NE	2/15	0.882	NE
nad4:1417	0.999	nonsynonymous	CAC>TAC	His-to-Tyr	2/2	0.500	NE	2/16	0.889	NE
nad4:1433	0.999	nonsynonymous	CCG>CTG	Pro-to-Leu	2/2	0.500	NE	2/14	0.875	NE
nad4:1438	0.991	nonsynonymous	CGC>TGC	Arg-to-Cys	0/3	1.000	NE	0/15	1.000	NE
nad4L:2	0.914	nonsynonymous	ACG>ATG	Thr-to-Met	0/0	NA	NE	0/0	NA	NE
nad4L:8	0.887	nonsynonymous	CCT>CTT	Pro-to-Leu	7/3	0.300	NE	12/0	0.000	NE
nad4L:21	0.744	synonymous	TTC>TTT	Phe-to-Phe	10/2	0.167	NE	16/0	0.000	NE
nad4L:41	0.999	nonsynonymous	TCT>TTT	Ser-to-Phe	0/12	1.000	NE	0/17	1.000	NE

Site ID	Score	Codon effect	Codon change	AA change	L1 C/T	L1 T fraction	L1 support	L2 C/T	L2 T fraction	L2 support
nad4L:47	0.998	nonsynonymous	TCA>TTA	Ser-to-Leu	0/14	1.000	NE	0/18	1.000	NE
nad4L:55	0.992	nonsynonymous	CGG>TGG	Arg-to-Trp	0/13	1.000	NE	0/20	1.000	Yes
nad4L:86	0.982	nonsynonymous	CCT>CTT	Pro-to-Leu	0/15	1.000	NE	0/23	1.000	Yes
nad4L:95	1.000	nonsynonymous	TCA>TTA	Ser-to-Leu	0/14	1.000	NE	0/23	1.000	Yes
nad4L:100	1.000	nonsynonymous	CCA>TCA	Pro-to-Ser	0/14	1.000	NE	1/22	0.957	Yes
nad4L:110	0.999	nonsynonymous	TCA>TTA	Ser-to-Leu	0/13	1.000	NE	0/23	1.000	Yes
nad4L:131	1.000	nonsynonymous	TCG>TTG	Ser-to-Leu	0/11	1.000	NE	0/26	1.000	Yes
nad4L:150	0.814	synonymous	TCC>TCT	Ser-to-Ser	12/2	0.143	NE	24/4	0.143	Yes
nad4L:158	1.000	nonsynonymous	TCG>TTG	Ser-to-Leu	0/12	1.000	NE	0/29	1.000	Yes
nad4L:179	1.000	nonsynonymous	TCA>TTA	Ser-to-Leu	0/15	1.000	NE	0/27	1.000	Yes
nad4L:188	0.999	nonsynonymous	TCA>TTA	Ser-to-Leu	1/18	0.947	NE	3/32	0.914	Yes
nad4L:196	0.769	nonsynonymous	CCA>TCA	Pro-to-Ser	16/0	0.000	NE	33/1	0.029	No
nad4L:197	0.999	nonsynonymous	CCA>CTA	Pro-to-Leu	1/16	0.941	NE	3/34	0.919	Yes
nad4L:230	0.998	nonsynonymous	TCA>TTA	Ser-to-Leu	0/19	1.000	NE	0/36	1.000	Yes
nad4L:252	0.602	synonymous	TTC>TTT	Phe-to-Phe	16/0	0.000	NE	33/0	0.000	No
nad4L:281	0.999	nonsynonymous	TCT>TTT	Ser-to-Phe	0/12	1.000	NE	0/23	1.000	Yes
nad5:609	0.511	synonymous	GTC>GTT	Val-to-Val	3/0	0.000	NE	43/1	0.023	No
nad5:1567	0.748	synonymous	CTG>TTG	Leu-to-Leu	10/0	0.000	NE	26/0	0.000	No
nad5:1918	0.998	nonsynonymous	CGT>TGT	Arg-to-Cys	1/7	0.875	NE	10/87	0.897	Yes
nad5:1958	0.991	nonsynonymous	TCG>TTG	Ser-to-Leu	1/7	0.875	NE	13/87	0.870	Yes
nad6:25	0.808	nonsynonymous	CCT>TCT	Pro-to-Ser	9/0	0.000	NE	19/0	0.000	NE
nad6:26	0.998	nonsynonymous	CCT>CTT	Pro-to-Leu	0/9	1.000	NE	1/17	0.944	NE
nad6:88	0.981	nonsynonymous	CTC>TTC	Leu-to-Phe	0/9	1.000	NE	5/25	0.833	Yes
nad6:103	0.979	nonsynonymous	CGC>TGC	Arg-to-Cys	0/8	1.000	NE	1/26	0.963	Yes
nad6:144	0.628	synonymous	TTC>TTT	Phe-to-Phe	6/0	0.000	NE	34/0	0.000	No
nad6:161	1.000	nonsynonymous	CCA>CTA	Pro-to-Leu	1/4	0.800	NE	1/31	0.969	Yes
nad6:169	1.000	nonsynonymous	CAT>TAT	His-to-Tyr	1/4	0.800	NE	0/25	1.000	Yes
nad6:191	0.991	nonsynonymous	TCA>TTA	Ser-to-Leu	1/5	0.833	NE	4/22	0.846	Yes
nad6:446	0.999	nonsynonymous	TCC>TTC	Ser-to-Phe	0/9	1.000	NE	2/34	0.944	Yes

Site ID	Score	Codon effect	Codon change	AA change	L1 C/T	L1 T fraction	L1 support	L2 C/T	L2 T fraction	L2 support
nad6:447	0.502	synonymous	TCC>TCT	Ser-to-Ser	9/0	0.000	NE	39/0	0.000	No
nad7:24	0.542	synonymous	ATC>ATT	Ile-to-Ile	15/0	0.000	NE	15/0	0.000	NE
nad7:38	0.999	nonsynonymous	TCG>TTG	Ser-to-Leu	0/14	1.000	NE	1/15	0.938	NE
nad7:45	0.997	synonymous	TTC>TTT	Phe-to-Phe	3/11	0.786	NE	14/2	0.125	NE
nad7:77	0.991	nonsynonymous	TCA>TTA	Ser-to-Leu	0/15	1.000	NE	2/15	0.882	NE
nad7:83	1.000	nonsynonymous	TCA>TTA	Ser-to-Leu	1/15	0.938	NE	3/14	0.824	NE
nad7:137	0.999	nonsynonymous	TCA>TTA	Ser-to-Leu	2/19	0.905	Yes	3/12	0.800	NE
nad7:200	0.995	nonsynonymous	TCT>TTT	Ser-to-Phe	1/17	0.944	NE	2/12	0.857	NE
nad7:209	0.989	nonsynonymous	TCA>TTA	Ser-to-Leu	2/9	0.818	NE	0/10	1.000	NE
nad7:224	1.000	nonsynonymous	ACG>ATG	Thr-to-Met	2/11	0.846	NE	1/16	0.941	NE
nad7:244	0.994	nonsynonymous	CAT>TAT	His-to-Tyr	1/13	0.929	NE	0/21	1.000	Yes
nad7:251	0.935	nonsynonymous	TCA>TTA	Ser-to-Leu	2/12	0.857	NE	1/18	0.947	NE
nad7:276	0.505	synonymous	TGC>TGT	Cys-to-Cys	14/0	0.000	NE	21/0	0.000	No
nad7:315	0.610	synonymous	TTC>TTT	Phe-to-Phe	16/1	0.059	NE	22/0	0.000	No
nad7:316	0.997	nonsynonymous	CGT>TGT	Arg-to-Cys	0/17	1.000	NE	0/21	1.000	Yes
nad7:335	0.999	nonsynonymous	TCA>TTA	Ser-to-Leu	0/17	1.000	NE	1/23	0.958	Yes
nad7:344	0.999	nonsynonymous	TCA>TTA	Ser-to-Leu	0/17	1.000	NE	1/22	0.957	Yes
nad7:383	1.000	nonsynonymous	TCA>TTA	Ser-to-Leu	0/13	1.000	NE	1/24	0.960	Yes
nad7:533	0.992	nonsynonymous	TCC>TTC	Ser-to-Phe	0/39	1.000	Yes	1/27	0.964	Yes
nad7:534	0.523	synonymous	TCC>TCT	Ser-to-Ser	39/1	0.025	No	27/1	0.036	No
nad7:578	1.000	nonsynonymous	TCA>TTA	Ser-to-Leu	0/37	1.000	Yes	2/36	0.947	Yes
nad7:679	0.987	nonsynonymous	CCA>TCA	Pro-to-Ser	1/15	0.938	NE	6/28	0.824	Yes
nad7:698	1.000	nonsynonymous	TCG>TTG	Ser-to-Leu	0/15	1.000	NE	2/33	0.943	Yes
nad7:724	1.000	nonsynonymous	CAT>TAT	His-to-Tyr	0/20	1.000	Yes	1/39	0.975	Yes
nad7:734	1.000	nonsynonymous	TCG>TTG	Ser-to-Leu	0/21	1.000	Yes	1/34	0.971	Yes
nad7:739	0.999	nonsynonymous	CCT>TCT	Pro-to-Ser	0/22	1.000	Yes	2/34	0.944	Yes
nad7:740	0.999	nonsynonymous	CCT>CTT	Pro-to-Leu	0/20	1.000	Yes	1/35	0.972	Yes
nad7:769	1.000	nonsynonymous	CGC>TGC	Arg-to-Cys	1/21	0.955	Yes	1/20	0.952	Yes
nad7:926	0.994	nonsynonymous	TCA>TTA	Ser-to-Leu	9/17	0.654	Yes	1/39	0.975	Yes

Site ID	Score	Codon effect	Codon change	AA change	L1 C/T	L1 T fraction	L1 support	L2 C/T	L2 T fraction	L2 support
nad7:963	0.940	synonymous	TCC>TCT	Ser-to-Ser	25/3	0.107	Yes	30/9	0.231	Yes
nad7:1050	0.966	synonymous	CCC>CCT	Pro-to-Pro	7/11	0.611	NE	5/31	0.861	Yes
nad7:1057	0.998	nonsynonymous	CGT>TGT	Arg-to-Cys	0/17	1.000	NE	0/36	1.000	Yes
nad7:1079	0.999	nonsynonymous	TCT>TTT	Ser-to-Phe	2/14	0.875	NE	0/46	1.000	Yes
nad7:1103	1.000	nonsynonymous	TCT>TTT	Ser-to-Phe	0/13	1.000	NE	0/44	1.000	Yes
nad7:1124	1.000	nonsynonymous	CCA>CTA	Pro-to-Leu	2/9	0.818	NE	0/40	1.000	Yes
nad7:1137	0.941	synonymous	GTC>GTT	Val-to-Val	12/0	0.000	NE	37/0	0.000	No
nad7:1166	1.000	nonsynonymous	TCT>TTT	Ser-to-Phe	0/12	1.000	NE	0/35	1.000	Yes
nad9:92	0.995	nonsynonymous	TCT>TTT	Ser-to-Phe	2/2	0.500	NE	1/28	0.966	Yes
nad9:167	0.998	nonsynonymous	TCG>TTG	Ser-to-Leu	4/2	0.333	NE	0/35	1.000	Yes
nad9:190	1.000	nonsynonymous	CAT>TAT	His-to-Tyr	5/2	0.286	NE	0/36	1.000	Yes
nad9:298	0.996	nonsynonymous	CCG>TCG	Pro-to-Ser	7/5	0.417	NE	0/39	1.000	Yes
nad9:328	1.000	nonsynonymous	CGG>TGG	Arg-to-Trp	6/5	0.455	NE	0/34	1.000	Yes
nad9:368	1.000	nonsynonymous	TCC>TTC	Ser-to-Phe	2/4	0.667	NE	0/54	1.000	Yes
nad9:439	0.998	nonsynonymous	CTT>TTT	Leu-to-Phe	1/4	0.800	NE	0/58	1.000	Yes
nad9:534	0.524	synonymous	TTC>TTT	Phe-to-Phe	0/0	NA	NE	48/0	0.000	No
rpl10:9	0.540	synonymous	TTC>TTT	Phe-to-Phe	1/0	0.000	NE	1/6	0.857	NE
rpl10:86	0.894	nonsynonymous	TCA>TTA	Ser-to-Leu	2/2	0.500	NE	1/7	0.875	NE
rpl10:104	0.995	nonsynonymous	TCG>TTG	Ser-to-Leu	2/2	0.500	NE	1/6	0.857	NE
rpl10:136	0.794	nonsynonymous	CCA>TCA	Pro-to-Ser	0/4	1.000	NE	0/7	1.000	NE
rpl10:137	0.994	nonsynonymous	CCA>CTA	Pro-to-Leu	3/1	0.250	NE	5/2	0.286	NE
rpl10:242	0.994	nonsynonymous	TCG>TTG	Ser-to-Leu	1/1	0.500	NE	0/8	1.000	NE
rpl10:317	0.983	nonsynonymous	TCA>TTA	Ser-to-Leu	1/1	0.500	NE	0/14	1.000	NE
rpl10:333	0.804	synonymous	TAC>TAT	Tyr-to-Tyr	2/0	0.000	NE	12/0	0.000	NE
rpl10:361	0.532	synonymous	CTA>TTA	Leu-to-Leu	1/0	0.000	NE	11/0	0.000	NE
rpl16:321	0.837	synonymous	TCC>TCT	Ser-to-Ser	24/0	0.000	No	87/0	0.000	No
rps1:23	0.678	nonsynonymous	TCA>TTA	Ser-to-Leu	0/0	NA	NE	0/0	NA	NE
rps1:42	0.938	synonymous	TCC>TCT	Ser-to-Ser	0/0	NA	NE	0/0	NA	NE
rps1:66	0.615	synonymous	AAC>AAT	Asn-to-Asn	0/0	NA	NE	0/0	NA	NE

Site ID	Score	Codon effect	Codon change	AA change	L1 C/T	L1 T fraction	L1 support	L2 C/T	L2 T fraction	L2 support
rps1:125	0.949	nonsynonymous	CCT>CTT	Pro-to-Leu	0/0	NA	NE	0/0	NA	NE
rps1:158	0.996	nonsynonymous	CCT>CTT	Pro-to-Leu	0/0	NA	NE	0/0	NA	NE
rps1:209	0.978	nonsynonymous	TCC>TTC	Ser-to-Phe	86/0	0.000	No	34/0	0.000	No
rps1:318	0.550	synonymous	TCC>TCT	Ser-to-Ser	152/0	0.000	No	40/0	0.000	No
rps12:201	0.541	synonymous	ATC>ATT	Ile-to-Ile	277/42	0.132	Yes	609/36	0.056	Yes
rps3:64	1.000	nonsynonymous	CGG>TGG	Arg-to-Trp	2/8	0.800	NE	1/9	0.900	NE
rps3:69	0.983	synonymous	TTC>TTT	Phe-to-Phe	6/3	0.333	NE	6/4	0.400	NE
rps3:512	0.986	nonsynonymous	TCA>TTA	Ser-to-Leu	10/0	0.000	NE	20/0	0.000	No
rps3:699	0.577	synonymous	CCC>CCT	Pro-to-Pro	2/0	0.000	NE	28/0	0.000	No
rps3:1031	0.999	nonsynonymous	CCA>CTA	Pro-to-Leu	5/12	0.706	NE	6/8	0.571	NE
rps4:50	0.885	nonsynonymous	TCA>TTA	Ser-to-Leu	13/0	0.000	NE	21/0	0.000	No
rps4:145	0.998	nonsynonymous	CCG>TCG	Pro-to-Ser	7/16	0.696	Yes	4/30	0.882	Yes
rps4:176	0.996	nonsynonymous	TCA>TTA	Ser-to-Leu	9/15	0.625	Yes	4/28	0.875	Yes
rps4:196	1.000	nonsynonymous	CCC>TCC	Pro-to-Ser	2/14	0.875	NE	1/30	0.968	Yes
rps4:205	0.998	nonsynonymous	CAT>TAT	His-to-Tyr	3/14	0.824	NE	1/33	0.971	Yes
rps4:269	0.998	nonsynonymous	CCA>CTA	Pro-to-Leu	0/9	1.000	NE	4/29	0.879	Yes
rps4:278	0.972	nonsynonymous	CCA>CTA	Pro-to-Leu	0/9	1.000	NE	3/33	0.917	Yes
rps4:290	0.997	nonsynonymous	TCG>TTG	Ser-to-Leu	0/9	1.000	NE	3/36	0.923	Yes
rps4:302	0.998	nonsynonymous	CCG>CTG	Pro-to-Leu	0/8	1.000	NE	4/34	0.895	Yes
rps4:347	0.999	nonsynonymous	CCG>CTG	Pro-to-Leu	0/6	1.000	NE	3/34	0.919	Yes
rps4:452	0.545	nonsynonymous	ACC>ATC	Thr-to-Ile	3/0	0.000	NE	22/0	0.000	No
rps4:476	0.581	nonsynonymous	TCT>TTT	Ser-to-Phe	4/0	0.000	NE	25/0	0.000	No
rps4:494	0.804	nonsynonymous	TCC>TTC	Ser-to-Phe	0/4	1.000	NE	2/24	0.923	Yes
rps4:705	0.726	synonymous	TTC>TTT	Phe-to-Phe	14/0	0.000	NE	29/0	0.000	No
rps4:852	0.539	synonymous	ACC>ACT	Thr-to-Thr	11/0	0.000	NE	13/0	0.000	NE
rps4:926	0.995	nonsynonymous	TCG>TTG	Ser-to-Leu	0/5	1.000	NE	0/16	1.000	NE
rps4:937	0.991	nonsynonymous	CAT>TAT	His-to-Tyr	0/5	1.000	NE	1/17	0.944	NE
rps4:947	1.000	nonsynonymous	CCA>CTA	Pro-to-Leu	0/7	1.000	NE	2/20	0.909	Yes
rps4:962	0.999	nonsynonymous	TCT>TTT	Ser-to-Phe	0/5	1.000	NE	1/20	0.952	Yes

Site ID	Score	Codon effect	Codon change	AA change	L1 C/T	L1 T fraction	L1 support	L2 C/T	L2 T fraction	L2 support
rps4:1013	0.973	nonsynonymous	CCA>CTA	Pro-to-Leu	0/6	1.000	NE	1/17	0.944	NE
sdh3:54	0.936	synonymous	CTC>CTT	Leu-to-Leu	17/0	0.000	NE	47/0	0.000	No
sdh3:74	0.998	nonsynonymous	TCC>TTC	Ser-to-Phe	0/19	1.000	NE	1/45	0.978	Yes
sdh3:188	0.807	nonsynonymous	TCT>TTT	Ser-to-Phe	5/90	0.947	Yes	1/190	0.995	Yes
sdh3:204	0.972	synonymous	ATC>ATT	Ile-to-Ile	105/0	0.000	No	205/0	0.000	No
sdh3:284	0.859	nonsynonymous	TCG>TTG	Ser-to-Leu	85/0	0.000	No	185/0	0.000	No
sdh4:122	0.973	nonsynonymous	ACC>ATC	Thr-to-Ile	51/0	0.000	No	85/0	0.000	No
sdh4:155	0.993	nonsynonymous	CCA>CTA	Pro-to-Leu	36/18	0.333	Yes	46/52	0.531	Yes
sdh4:171	0.704	synonymous	ATC>ATT	Ile-to-Ile	57/0	0.000	No	103/0	0.000	No
sdh4:199	0.679	synonymous	CTA>TTA	Leu-to-Leu	52/0	0.000	No	111/0	0.000	No
sdh4:259	0.998	nonsynonymous	CAT>TAT	His-to-Tyr	20/15	0.429	Yes	27/57	0.679	Yes
sdh4:348	0.775	synonymous	CTC>CTT	Leu-to-Leu	12/1	0.077	NE	25/1	0.038	No
sdh4:353	0.946	nonsynonymous	TCT>TTT	Ser-to-Phe	2/8	0.800	NE	0/23	1.000	Yes

Notes: L1 = SRR13338383; L2 = SRR13338437; AA = amino acid. Site ID gives gene and 1-based CDS position. C/T = observed C/T read counts; T fraction = T/(C+T). Deepred-Mt prediction threshold: score ≥0.50. Support (Yes) requires C+T depth ≥20, ≥3 T reads, and T fraction ≥0.05; No = evaluable but unsupported; NE = insufficient coverage; NA = undefined fraction at zero coverage. Overall, 211/475 sites have RNA-seq support.

Table S2. Interspersed repeat pairs and HiFi support for alternative local connections in *Rhododendron spinuliferum* (n = 50 Vmatch records).

Repeat ID	Graph group	Length (bp)	Orientation	Identity (%)	Copy 1 location	Copy 2 location	Native reads	Alt. main	Alt. strict	Assessment
V01	V01	89001	Direct	100.00	ctg1:1–89001	ctg1:162412–251412	NA	NA	NA	Not assessable
V02	V02	1144	Direct	100.00	ctg1:161268–162411	ctg1:540624–541767	1082	NA	NA	Not assessable
V03	V03	1020	Direct	100.00	ctg1:9750–10769	ctg2:1–1020	564	566	461	Supported
V04	V03	1020	Direct	100.00	ctg1:172161–173180	ctg2:1–1020	564	566	461	Supported
V05	V05	721	Inverted	100.00	ctg1:503046–503766	ctg3:111214–111934	1323	23	18	Supported
V06	V06	554	Inverted	99.82	ctg1:438197–438750	ctg2:28431–28984	0	0	0	Insufficient
V07	V07	398	Inverted	99.75	ctg1:438459–438856	ctg2:28325–28722	0	0	0	Insufficient
V08	V08	386	Direct	100.00	ctg1:390465–390850	ctg3:61680–62065	1656	28	24	Supported
V09	V09	385	Inverted	99.22	ctg1:503912–504296	ctg3:15045–15429	0	0	0	Insufficient
V10	V10	350	Inverted	100.00	ctg1:304585–304934	ctg1:444858–445207	1303	9	8	Supported
V11	V11	337	Inverted	100.00	ctg1:393703–394039	ctg2:35399–35735	478	557	474	Supported
V12	V12	219	Inverted	100.00	ctg1:14988–15206	ctg1:32524–32742	1323	2	2	Insufficient
V13	V12	219	Inverted	100.00	ctg1:14988–15206	ctg1:194935–195153	1323	2	2	Insufficient
V14	V12	219	Inverted	100.00	ctg1:32524–32742	ctg1:177399–177617	1323	2	2	Insufficient
V15	V12	219	Inverted	100.00	ctg1:177399–177617	ctg1:194935–195153	1323	2	2	Insufficient
V16	V16	215	Inverted	100.00	ctg3:102011–102225	ctg3:197963–198177	1418	3	2	Insufficient
V17	V17	207	Inverted	99.52	ctg3:14999–15205	ctg3:63066–63272	0	0	0	Insufficient
V18	V18	194	Inverted	100.00	ctg1:439876–440069	ctg3:158581–158774	1294	0	0	Insufficient
V19	V17	182	Inverted	100.00	ctg3:15024–15205	ctg3:63066–63247	1406	1	1	Insufficient
V20	V20	174	Direct	100.00	ctg1:273–446	ctg1:143948–144121	1783	8	8	Supported
V21	V20	174	Direct	100.00	ctg1:143948–144121	ctg1:162684–162857	1782	8	8	Supported
V22	V22	167	Direct	100.00	ctg1:51013–51179	ctg1:126365–126531	1274	2	2	Insufficient
V23	V22	167	Direct	100.00	ctg1:126365–126531	ctg1:213424–213590	1274	2	2	Insufficient
V24	V24	168	Inverted	98.81	ctg1:18932–19099	ctg3:173748–173915	0	0	0	Insufficient
V25	V24	168	Inverted	98.81	ctg1:181343–181510	ctg3:173748–173915	0	0	0	Insufficient
V26	V26	161	Direct	98.14	ctg1:504136–504296	ctg3:63066–63226	0	0	0	Insufficient
V27	V27	159	Direct	98.11	ctg2:15698–15856	ctg3:111068–111226	1	0	0	Insufficient
V28	V28	151	Inverted	99.34	ctg1:14871–15021	ctg1:32746–32896	0	0	0	Insufficient
V29	V28	151	Inverted	99.34	ctg1:14871–15021	ctg1:195157–195307	0	0	0	Insufficient
V30	V28	151	Inverted	99.34	ctg1:32746–32896	ctg1:177282–177432	0	0	0	Insufficient
V31	V28	151	Inverted	99.34	ctg1:177282–177432	ctg1:195157–195307	0	0	0	Insufficient
V32	V28	142	Inverted	100.00	ctg1:32755–32896	ctg1:177282–177423	1326	1	1	Insufficient
V33	V33	134	Inverted	100.00	ctg1:330368–330501	ctg2:32040–32173	1302	1	1	Insufficient
V34	V34	139	Direct	97.84	ctg2:15225–15363	ctg3:14844–14982	0	0	0	Insufficient
V35	V34	130	Direct	98.46	ctg2:15238–15367	ctg3:14857–14986	0	0	0	Insufficient

Repeat ID	Graph group	Length (bp)	Orientation	Identity (%)	Copy 1 location	Copy 2 location	Native reads	Alt. main	Alt. strict	Assessment
V36	V36	122	Direct	98.36	ctg1:92234–92355	ctg3:202024–202145	1	0	0	Insufficient
V37	V37	112	Direct	100.00	ctg1:439698–439809	ctg3:189491–189602	1339	0	0	Insufficient
V38	V38	119	Inverted	98.32	ctg1:312172–312290	ctg1:413611–413729	1	0	0	Insufficient
V39	V39	122	Inverted	97.54	ctg1:133271–133392	ctg1:277863–277984	0	0	0	Insufficient
V40	V40	120	Direct	97.50	ctg3:54276–54395	ctg3:173052–173171	0	0	0	Insufficient
V41	V41	105	Inverted	100.00	ctg1:12282–12386	ctg1:23167–23271	1210	5	5	Supported
V42	V41	105	Inverted	100.00	ctg1:12282–12386	ctg1:185578–185682	1210	5	5	Supported
V43	V41	105	Inverted	100.00	ctg1:23167–23271	ctg1:174693–174797	1210	5	5	Supported
V44	V41	105	Inverted	100.00	ctg1:174693–174797	ctg1:185578–185682	1210	5	5	Supported
V45	V07	105	Inverted	100.00	ctg1:438752–438856	ctg2:28325–28429	1276	9	8	Supported
V46	V24	103	Inverted	99.03	ctg1:18997–19099	ctg3:173748–173850	0	0	0	Insufficient
V47	V24	103	Inverted	99.03	ctg1:181408–181510	ctg3:173748–173850	0	0	0	Insufficient
V48	V38	103	Inverted	99.03	ctg1:312188–312290	ctg1:413611–413713	0	0	0	Insufficient
V49	V49	106	Inverted	98.11	ctg1:113235–113340	ctg1:408515–408620	0	0	0	Insufficient
V50	V50	101	Direct	99.01	ctg1:135713–135813	ctg1:426951–427051	0	0	0	Insufficient

Notes: Coordinates are 1-based inclusive. Vmatch used -best 50; graph groups combine equivalent or overlapping repeat records, whose read counts must not be summed. Main filter: one continuous HiFi alignment across the repeat and ≥500 bp per flank, MAPQ ≥20, identity ≥99%, ΔAS ≥50, graph-unique 31-mers, and single-HSP BLASTN confirmation. Supported = ≥3 main-filter and ≥2 stringent-filter reads for one alternative connection; NA = not assessable. Insufficient support does not establish absence. V02 was not independently distinguishable; V01 (89,001 bp) exceeded the longest available read.

Table S3. Mitochondrial gene-copy annotation and exon coordinates in *Rhododendron spinuliferum*.

Panel A. Gene/copy records and genomic locations (n = 72)

Record ID	Gene	Product	Gene category	Record type	Functional status	Path	Topology	Start	End	Strand	Copy ID	Exons
ctg000001c.rps19.67	*rps19*	—	Small ribosomal subunit	pseudogene-like/partial fragment	partial/pseudogene-like fragment	ctg000001c	circular	37015	37254	+	copy_1	1
ctg000001c.rps3.77	*rps3*	ribosomal protein S3	Small ribosomal subunit	CDS	functional	ctg000001c	circular	37261	40108	+	copy_1	2
ctg000001c.rpl16.61	*rpl16*	ribosomal protein L16	Large ribosomal subunit	CDS	functional	ctg000001c	circular	40080	40514	+	copy_1	1
ctg000001c.trnM-CAU.92	*trnM-CAU*	tRNA-Met	Transfer RNA	tRNA	functional	ctg000001c	circular	40805	40874	+	copy_1	1
ctg000001c.trnG-GCC.100	*trnG-GCC*	tRNA-Gly	Transfer RNA	tRNA	functional	ctg000001c	circular	53806	53877	+	copy_1	1
ctg000001c.trnQ-UUG.120	*trnQ-UUG*	tRNA-Gln	Transfer RNA	tRNA	functional	ctg000001c	circular	56089	56160	+	copy_1	1
ctg000001c.trnfM-CAU.118	*trnfM-CAU*	tRNA-Met	Transfer RNA	tRNA	functional	ctg000001c	circular	82397	82470	-	copy_1	1
ctg000001c.nad2.5	*nad2*	NADH dehydrogenase subunit 2	NADH dehydrogenase	trans-spliced genomic component	functional; trans-spliced genomic component	ctg000001c	circular	110535	112285	-	component_ctg000001c	2
ctg000001c.sdh3.86	*sdh3*	succinate dehydrogenase subunit 3	Succinate dehydrogenase	CDS	functional	ctg000001c	circular	112893	113213	-	copy_1	1
ctg000001c.trnS-UGA.124	*trnS-UGA*	tRNA-Ser	Transfer RNA	tRNA	functional	ctg000001c	circular	119141	119227	+	copy_1	1
ctg000001c.trnM-CAU.116	*trnM-CAU*	tRNA-Met	Transfer RNA	tRNA	functional	ctg000001c	circular	132228	132301	-	copy_2	1
ctg000001c.trnD-GUC.96	*trnD-GUC*	tRNA-Asp	Transfer RNA	tRNA	functional	ctg000001c	circular	132451	132524	+	copy_1	1
ctg000001c.atp6.16	*atp6*	ATPase subunit 6	ATP synthase	CDS	functional	ctg000001c	circular	133480	134355	+	copy_1	1
ctg000001c.rpl5.132	*rpl5*	—	Large ribosomal subunit	pseudogene-like/partial fragment	partial/pseudogene-like fragment	ctg000001c	circular	146378	146488	+	copy_1	1
ctg000001c.rps19.70	*rps19*	—	Small ribosomal subunit	pseudogene-like/partial fragment	partial/pseudogene-like fragment	ctg000001c	circular	199426	199665	+	copy_2	1
ctg000001c.rps3.73	*rps3*	ribosomal protein S3	Small ribosomal subunit	CDS	functional	ctg000001c	circular	199672	202519	+	copy_2	2
ctg000001c.rpl16.64	*rpl16*	ribosomal protein L16	Large ribosomal subunit	CDS	functional	ctg000001c	circular	202491	202925	+	copy_2	1
ctg000001c.trnM-CAU.94	*trnM-CAU*	tRNA-Met	Transfer RNA	tRNA	functional	ctg000001c	circular	203216	203285	+	copy_3	1
ctg000001c.trnG-GCC.102	*trnG-GCC*	tRNA-Gly	Transfer RNA	tRNA	functional	ctg000001c	circular	216217	216288	+	copy_2	1
ctg000001c.trnQ-UUG.122	*trnQ-UUG*	tRNA-Gln	Transfer RNA	tRNA	functional	ctg000001c	circular	218500	218571	+	copy_2	1
ctg000001c.trnfM-CAU.114	*trnfM-CAU*	tRNA-Met	Transfer RNA	tRNA	functional	ctg000001c	circular	244808	244881	-	copy_2	1
ctg000001c.trnW-CCA.126	*trnW-CCA*	tRNA-Trp	Transfer RNA	tRNA	functional	ctg000001c	circular	276501	276574	-	copy_1	1
ctg000001c.nad9.55	*nad9*	NADH dehydrogenase subunit 9	NADH dehydrogenase	CDS	functional	ctg000001c	circular	277261	277833	-	copy_1	1
ctg000001c.trnK-UUU.106	*trnK-UUU*	tRNA-Lys	Transfer RNA	tRNA	functional	ctg000001c	circular	296870	296942	+	copy_1	1
ctg000001c.trnI-CAU.108	*trnI-CAU*	tRNA-Met	Transfer RNA	tRNA	functional	ctg000001c	circular	297180	297256	+	copy_1	1
ctg000001c.rps4.81	*rps4*	ribosomal protein S4	Small ribosomal subunit	CDS	functional	ctg000001c	circular	345580	346638	+	copy_1	1
ctg000001c.nad6.52	*nad6*	NADH dehydrogenase subunit 6	NADH dehydrogenase	CDS	functional	ctg000001c	circular	347289	347906	+	copy_1	1
ctg000001c.trnA-UGC.128	*trnA-UGC*	tRNA-Ala	Transfer RNA	tRNA	functional	ctg000001c	circular	349889	349973	+	copy_1	1
ctg000001c.ccmFC.28	*ccmFC*	cytochrome c biogenesis FC	Cytochrome c biogenesis	CDS	functional	ctg000001c	circular	353604	355878	+	copy_1	2
ctg000001c.ccmC.25	ccmC	cytochrome c biogenesis C	Cytochrome c biogenesis	CDS	functional	ctg000001c	circular	361921	362754	-	copy_1	1
ctg000001c.atp4.13	*atp4*	ATPase subunit 4	ATP synthase	CDS	functional	ctg000001c	circular	364539	365117	-	copy_1	1

Record ID	Gene	Product	Gene category	Record type	Functional status	Path	Topology	Start	End	Strand	Copy ID	Exons
ctg000001c.nad4L.49	*nad4L*	NADH dehydrogenase subunit 4L	NADH dehydrogenase	CDS	functional	ctg000001c	circular	365303	365605	-	copy_1	1
ctg000001c.nad1.1	*nad1*	NADH dehydrogenase subunit 1	NADH dehydrogenase	trans-spliced genomic component	functional; trans-spliced genomic component	ctg000001c	circular	397196	401019	-	component_ctg000001c	2
ctg000001c.matR.38	*matR*	maturase R	Maturase	CDS	functional	ctg000001c	circular	398331	400298	-	copy_1	1
ctg000001c.mttB.41	*mttB*	transport membrane protein	Transport membrane protein	CDS	functional	ctg000001c	circular	408625	409413	+	copy_1	1
ctg000001c.rps1.130	*rps1*	ribosomal protein S1	Small ribosomal subunit	CDS	functional	ctg000001c	circular	428455	428781	+	copy_1	1
ctg000001c.trnM-CAU.110	*trnM-CAU*	tRNA-Met	Transfer RNA	tRNA	functional	ctg000001c	circular	437245	437317	+	copy_4	1
ctg000001c.atp8.19	*atp8*	ATPase subunit 8	ATP synthase	CDS	functional	ctg000001c	circular	439995	440474	+	copy_1	1
ctg000001c.cox3.35	*cox3*	cytochrome c oxidase subunit 3	Cytochrome c oxidase	CDS	functional	ctg000001c	circular	441104	441901	+	copy_1	1
ctg000001c.sdh4.89	*sdh4*	succinate dehydrogenase subunit 4	Succinate dehydrogenase	CDS	functional	ctg000001c	circular	441829	442227	+	copy_1	1
ctg000001c.ccmB.22	ccmB	cytochrome c biogenesis B	Cytochrome c biogenesis	CDS	functional	ctg000001c	circular	456758	457378	+	copy_1	1
ctg000001c.rpl10.58	*rpl10*	ribosomal protein L10	Large ribosomal subunit	CDS	functional	ctg000001c	circular	457658	458149	-	copy_1	1
ctg000001c.rrn26.84	*rrn26*	26S ribosomal RNA	Ribosomal RNA	rRNA	functional	ctg000001c	circular	478897	482231	+	copy_1	1
ctg000001c.trnfM-CAU.112	*trnfM-CAU*	tRNA-Met	Transfer RNA	tRNA	functional	ctg000001c	circular	482611	482684	+	copy_3	1
ctg000001c.nad4.44	*nad4*	NADH dehydrogenase subunit 4	NADH dehydrogenase	CDS	functional	ctg000001c	circular	496868	502346	-	copy_1	3
ctg000001c.trnH-GUG.104	*trnH-GUG*	tRNA-His	Transfer RNA	tRNA	functional	ctg000001c	circular	513328	513401	-	copy_1	1
ctg000001c.trnE-UUC.98	*trnE-UUC*	tRNA-Glu	Transfer RNA	tRNA	functional	ctg000001c	circular	515807	515878	+	copy_1	1
ctg000001c.nad5.9	*nad5*	NADH dehydrogenase subunit 5	NADH dehydrogenase	trans-spliced genomic component	functional; trans-spliced genomic component	ctg000001c	circular	534854	536503	+	component_ctg000001c	2
ctg000001c.ccmFN.32	*ccmFN*	cytochrome c biogenesis FN	Cytochrome c biogenesis	CDS	functional	ctg000001c	circular	538530	540269	+	copy_1	1
ctg000002l.cob.1	cob	apocytochrome b	Cytochrome b	CDS	functional	ctg000002l	linear	16102	17283	+	copy_1	1
ctg000002l.cox1.4	*cox1*	cytochrome c oxidase subunit 1	Cytochrome c oxidase	CDS	functional	ctg000002l	linear	27325	28908	-	copy_1	1
ctg000003c.rrn18.41	*rrn18*	18S ribosomal RNA	Ribosomal RNA	rRNA	functional	ctg000003c	circular	15083	16902	+	copy_1	1
ctg000003c.rrn5.43	*rrn5*	5S ribosomal RNA	Ribosomal RNA	rRNA	functional	ctg000003c	circular	17052	17168	+	copy_1	1
ctg000003c.nad7.31	*nad7*	NADH dehydrogenase subunit 7	NADH dehydrogenase	CDS	functional	ctg000003c	circular	32917	39157	-	copy_1	5
ctg000003c.trnN-GUU.48	*trnN-GUU*	tRNA-Asn	Transfer RNA	tRNA	functional	ctg000003c	circular	65690	65760	-	copy_1	1
ctg000003c.nad1.1	*nad1*	NADH dehydrogenase subunit 1	NADH dehydrogenase	trans-spliced genomic component	functional; trans-spliced genomic component	ctg000003c	circular	100317	100701	-	component_ctg000003c	3
ctg000003c.atp9.19	*atp9*	—	ATP synthase	pseudogene-like/partial fragment	partial/pseudogene-like fragment	ctg000003c	circular	102025	102228	-	copy_1	1
ctg000003c.trnP-UGG.58	*trnP-UGG*	tRNA-Pro	Transfer RNA	tRNA	functional	ctg000003c	circular	103193	103267	-	copy_1	1
ctg000003c.trnF-GAA.54	*trnF-GAA*	tRNA-Phe	Transfer RNA	tRNA	functional	ctg000003c	circular	103524	103597	-	copy_1	1
ctg000003c.trnS-GCU.60	*trnS-GCU*	tRNA-Ser	Transfer RNA	tRNA	functional	ctg000003c	circular	103946	104033	-	copy_1	1
ctg000003c.nad3.28	*nad3*	NADH dehydrogenase subunit 3	NADH dehydrogenase	CDS	functional	ctg000003c	circular	110206	110562	+	copy_1	1
ctg000003c.rps12.38	*rps12*	ribosomal protein S12	Small ribosomal subunit	CDS	functional	ctg000003c	circular	110610	110987	+	copy_1	1
ctg000003c.cox2.25	*cox2*	cytochrome c oxidase subunit 2	Cytochrome c oxidase	CDS	functional	ctg000003c	circular	111286	112083	+	copy_1	1
ctg000003c.nad2.6	*nad2*	NADH dehydrogenase subunit 2	NADH dehydrogenase	trans-spliced genomic component	functional; trans-spliced genomic component	ctg000003c	circular	124044	127781	-	component_ctg000003c	3

Record ID	Gene	Product	Gene category	Record type	Functional status	Path	Topology	Start	End	Strand	Copy ID	Exons
ctg000003c.trnY-GUA.62	*trnY-GUA*	tRNA-Tyr	Transfer RNA	tRNA	functional	ctg000003c	circular	128288	128370	-	copy_1	1
ctg000003c.trnN-GUU.56	*trnN-GUU*	tRNA-Asn	Transfer RNA	tRNA	functional	ctg000003c	circular	129409	129480	-	copy_2	1
ctg000003c.trnC-GCA.52	*trnC-GCA*	tRNA-Cys	Transfer RNA	tRNA	functional	ctg000003c	circular	130546	130616	-	copy_1	1
ctg000003c.trnR-UCG.50	*trnR-UCG*	tRNA-Arg	Transfer RNA	tRNA	functional	ctg000003c	circular	137210	138324	+	copy_1	2
ctg000003c.atp1.16	*atp1*	ATPase subunit 1	ATP synthase	CDS	functional	ctg000003c	circular	188073	189602	+	copy_1	1
ctg000003c.sdh4.45	*sdh4*	—	Succinate dehydrogenase	pseudogene-like/partial fragment	partial/pseudogene-like fragment	ctg000003c	circular	190401	190541	+	copy_2	1
ctg000003c.atp9.22	*atp9*	ATPase subunit 9	ATP synthase	CDS	functional	ctg000003c	circular	197939	198163	+	copy_2	1
ctg000003c.nad5.11	*nad5*	NADH dehydrogenase subunit 5	NADH dehydrogenase	trans-spliced genomic component	functional; trans-spliced genomic component	ctg000003c	circular	198490	200771	+	component_ctg000003c	3

Panel B. Exon structures, CDS lengths, and codon attributes (n = 72)

Record ID	Exon coordinates	Annotated CDS (bp)	Complete CDS (bp)	Protein (aa)	Genomic start	Genomic stop	Start codon	Stop codon	RNA-editing note
ctg000001c.rps19.67	ctg000001c:37015-37254(+)	N/A	Not available*	—	N/A	N/A	N/A	N/A	No
ctg000001c.rps3.77	ctg000001c:37261-37334(+); ctg000001c:38491-40108(+)	1692	1692	563	ATG	TAA	ATG	TAA	No
ctg000001c.rpl16.61	ctg000001c:40080-40514(+)	435	435	144	GTG	TAA	Not available*	TAA	No
ctg000001c.trnM-CAU.92	ctg000001c:40805-40874(+)	N/A	N/A	—	N/A	N/A	N/A	N/A	No
ctg000001c.trnG-GCC.100	ctg000001c:53806-53877(+)	N/A	N/A	—	N/A	N/A	N/A	N/A	No
ctg000001c.trnQ-UUG.120	ctg000001c:56089-56160(+)	N/A	N/A	—	N/A	N/A	N/A	N/A	No
ctg000001c.trnfM-CAU.118	ctg000001c:82397-82470(-)	N/A	N/A	—	N/A	N/A	N/A	N/A	No
ctg000001c.nad2.5	ctg000001c:112133-112285(-); ctg000001c:110535-110926(-)	545	Not available*	—	Not available*	Not available*	Not available*	Not available*	No
ctg000001c.sdh3.86	ctg000001c:112893-113213(-)	321	321	106	ATG	TGA	ATG	TGA	No
ctg000001c.trnS-UGA.124	ctg000001c:119141-119227(+)	N/A	N/A	—	N/A	N/A	N/A	N/A	No
ctg000001c.trnM-CAU.116	ctg000001c:132228-132301(-)	N/A	N/A	—	N/A	N/A	N/A	N/A	No
ctg000001c.trnD-GUC.96	ctg000001c:132451-132524(+)	N/A	N/A	—	N/A	N/A	N/A	N/A	No
ctg000001c.atp6.16	ctg000001c:133480-134355(+)	876	876	291	ATG	TAG	ATG	TAG	No
ctg000001c.rpl5.132	ctg000001c:146378-146488(+)	N/A	Not available*	—	N/A	N/A	N/A	N/A	No
ctg000001c.rps19.70	ctg000001c:199426-199665(+)	N/A	Not available*	—	N/A	N/A	N/A	N/A	No
ctg000001c.rps3.73	ctg000001c:199672-199745(+); ctg000001c:200902-202519(+)	1692	1692	563	ATG	TAA	ATG	TAA	No
ctg000001c.rpl16.64	ctg000001c:202491-202925(+)	435	435	144	GTG	TAA	Not available*	TAA	No
ctg000001c.trnM-CAU.94	ctg000001c:203216-203285(+)	N/A	N/A	—	N/A	N/A	N/A	N/A	No
ctg000001c.trnG-GCC.102	ctg000001c:216217-216288(+)	N/A	N/A	—	N/A	N/A	N/A	N/A	No
ctg000001c.trnQ-UUG.122	ctg000001c:218500-218571(+)	N/A	N/A	—	N/A	N/A	N/A	N/A	No
ctg000001c.trnfM-CAU.114	ctg000001c:244808-244881(-)	N/A	N/A	—	N/A	N/A	N/A	N/A	No
ctg000001c.trnW-CCA.126	ctg000001c:276501-276574(-)	N/A	N/A	—	N/A	N/A	N/A	N/A	No
ctg000001c.nad9.55	ctg000001c:277261-277833(-)	573	573	190	ATG	TAA	ATG	TAA	No
ctg000001c.trnK-UUU.106	ctg000001c:296870-296942(+)	N/A	N/A	—	N/A	N/A	N/A	N/A	No
ctg000001c.trnI-CAU.108	ctg000001c:297180-297256(+)	N/A	N/A	—	N/A	N/A	N/A	N/A	No
ctg000001c.rps4.81	ctg000001c:345580-346638(+)	1059	1059	352	ACG	TAA	ACG (probable; source note)	TAA	No
ctg000001c.nad6.52	ctg000001c:347289-347906(+)	618	618	205	ATG	TAA	ATG	TAA	No
ctg000001c.trnA-UGC.128	ctg000001c:349889-349973(+)	N/A	N/A	—	N/A	N/A	N/A	N/A	No
ctg000001c.ccmFC.28	ctg000001c:353604-354376(+); ctg000001c:355329-355878(+)	1323	1323	441	ATG	CGA	ATG	Created by C-to-U RNA editing (source note)	Yes
ctg000001c.ccmC.25	ctg000001c:361921-362754(-)	834	834	277	ATG	TAA	ATG	TAA	No
ctg000001c.atp4.13	ctg000001c:364539-365117(-)	579	579	192	ATG	TAA	ATG	TAA	No
ctg000001c.nad4L.49	ctg000001c:365303-365605(-)	303	303	100	ACG	TAA	Not available*	TAA	No
ctg000001c.nad1.1	ctg000001c:400961-401019(-); ctg000001c:397196-397454(-)	318	Not available*	—	Not available*	Not available*	Not available*	Not available*	No
ctg000001c.matR.38	ctg000001c:398331-400298(-)	1968	1968	655	ATG	TAG	ATG	TAG	No
ctg000001c.mttB.41	ctg000001c:408625-409413(+)	789	789	262	ATA	TAG	Not available*	TAG	No
ctg000001c.rps1.130	ctg000001c:428455-428781(+)	327	327	108	ATG	TAA	ATG	TAA	No
ctg000001c.trnM-CAU.110	ctg000001c:437245-437317(+)	N/A	N/A	—	N/A	N/A	N/A	N/A	No
ctg000001c.atp8.19	ctg000001c:439995-440474(+)	480	480	159	ATG	TAA	ATG	TAA	No
ctg000001c.cox3.35	ctg000001c:441104-441901(+)	798	798	265	ATG	TGA	ATG	TGA	No
ctg000001c.sdh4.89	ctg000001c:441829-442227(+)	399	399	132	ATG	TGA	ATG	TGA	No
ctg000001c.ccmB.22	ctg000001c:456758-457378(+)	621	621	206	ATG	TGA	ATG	TGA	No
ctg000001c.rpl10.58	ctg000001c:457658-458149(-)	492	492	163	ATG	TAA	ATG	TAA	No
ctg000001c.rrn26.84	ctg000001c:478897-482231(+)	N/A	N/A	—	N/A	N/A	N/A	N/A	No
ctg000001c.trnfM-CAU.112	ctg000001c:482611-482684(+)	N/A	N/A	—	N/A	N/A	N/A	N/A	No
ctg000001c.nad4.44	ctg000001c:501886-502346(-); ctg000001c:499526-500463(-); ctg000001c:496868-496956(-)	1488	1488	495	ATG	TGA	ATG	TGA	No
ctg000001c.trnH-GUG.104	ctg000001c:513328-513401(-)	N/A	N/A	—	N/A	N/A	N/A	N/A	No

Record ID	Exon coordinates	Annotated CDS (bp)	Complete CDS (bp)	Protein (aa)	Genomic start	Genomic stop	Start codon	Stop codon	RNA-editing note
ctg000001c.trnE-UUC.98	ctg000001c:515807-515878(+)	N/A	N/A	—	N/A	N/A	N/A	N/A	No
ctg000001c.nad5.9	ctg000001c:534854-535248(+); ctg000001c:536354-536503(+)	545	Not available*	—	Not available*	Not available*	Not available*	Not available*	No
ctg000001c.ccmFN.32	ctg000001c:538530-540269(+)	1740	1740	579	ATG	TGA	ATG	TGA	No
ctg000002l.cob.1	ctg000002l:16102-17283(+)	1182	1182	393	ATG	TGA	ATG	TGA	No
ctg000002l.cox1.4	ctg000002l:27325-28908(-)	1584	1584	527	ATG	TAA	ATG	TAA	No
ctg000003c.rrn18.41	ctg000003c:15083-16902(+)	N/A	N/A	—	N/A	N/A	N/A	N/A	No
ctg000003c.rrn5.43	ctg000003c:17052-17168(+)	N/A	N/A	—	N/A	N/A	N/A	N/A	No
ctg000003c.nad7.31	ctg000003c:39015-39157(-); ctg000003c:38124-38192(-); ctg000003c:36174-36640(-); ctg000003c:34876-35119(-); ctg000003c:32917-33178(-)	1185	1185	394	ATG	TAG	ATG	TAG	No
ctg000003c.trnN-GUU.48	ctg000003c:65690-65760(-)	N/A	N/A	—	N/A	N/A	N/A	N/A	No
ctg000003c.nad1.1	ctg000003c:100317-100701(-); ctg000003c:157497-157579(-); ctg000003c:156071-156262(-)	660	Not available*	—	Not available*	Not available*	Not available*	Not available*	No
ctg000003c.atp9.19	ctg000003c:102025-102228(-)	N/A	Not available*	—	N/A	N/A	N/A	N/A	No
ctg000003c.trnP-UGG.58	ctg000003c:103193-103267(-)	N/A	N/A	—	N/A	N/A	N/A	N/A	No
ctg000003c.trnF-GAA.54	ctg000003c:103524-103597(-)	N/A	N/A	—	N/A	N/A	N/A	N/A	No
ctg000003c.trnS-GCU.60	ctg000003c:103946-104033(-)	N/A	N/A	—	N/A	N/A	N/A	N/A	No
ctg000003c.nad3.28	ctg000003c:110206-110562(+)	357	357	118	ATG	TAA	ATG	TAA	No
ctg000003c.rps12.38	ctg000003c:110610-110987(+)	378	378	125	ATG	TGA	ATG	TGA	No
ctg000003c.cox2.25	ctg000003c:111286-112083(+)	798	798	265	ATG	TAA	ATG	TAA	No
ctg000003c.nad2.6	ctg000003c:127621-127781(-); ctg000003c:125734-126306(-); ctg000003c:124044-124231(-)	922	Not available*	—	Not available*	Not available*	Not available*	Not available*	No
ctg000003c.trnY-GUA.62	ctg000003c:128288-128370(-)	N/A	N/A	—	N/A	N/A	N/A	N/A	No
ctg000003c.trnN-GUU.56	ctg000003c:129409-129480(-)	N/A	N/A	—	N/A	N/A	N/A	N/A	No
ctg000003c.trnC-GCA.52	ctg000003c:130546-130616(-)	N/A	N/A	—	N/A	N/A	N/A	N/A	No
ctg000003c.trnR-UCG.50	ctg000003c:137210-137243(+); ctg000003c:138265-138324(+)	N/A	N/A	—	N/A	N/A	N/A	N/A	No
ctg000003c.atp1.16	ctg000003c:188073-189602(+)	1530	1530	509	ATG	TGA	ATG	TGA	No
ctg000003c.sdh4.45	ctg000003c:190401-190541(+)	N/A	Not available*	—	N/A	N/A	N/A	N/A	No
ctg000003c.atp9.22	ctg000003c:197939-198163(+)	225	225	75	ATG	CGA	ATG	Created by C-to-U RNA editing (source note)	Yes
ctg000003c.nad5.11	ctg000003c:198490-198719(+); ctg000003c:199556-200771(+); ctg000003c:62719-62740(-)	1468	Not available*	—	Not available*	Not available*	Not available*	Not available*	No

Panel C. Duplicated/partial gene status and annotation notes (n = 72)

Record ID	Duplicate	Focus duplicate	Partial	Pseudo-like	Trans-spliced	Source annotation note
ctg000001c.rps19.67	No	No	Yes	Yes	No	manual curation: rps19-like partial/pseudogene fragment; not counted as functional PCG | manual curation: rps19-like partial/pseudogene fragment; not counted as a functional protein-coding gene
ctg000001c.rps3.77	Yes	Yes	No	No	No	—
ctg000001c.rpl16.61	Yes	Yes	No	No	No	start codon is not determined
ctg000001c.trnM-CAU.92	Yes	No	No	No	No	unknown tRNA sources
ctg000001c.trnG-GCC.100	Yes	No	No	No	No	mitochondrion-native
ctg000001c.trnQ-UUG.120	Yes	No	No	No	No	mitochondrion-native
ctg000001c.trnfM-CAU.118	Yes	No	No	No	No	mitochondrion-native
ctg000001c.nad2.5	No	No	Yes	No	Yes	partial genomic component of trans-spliced nad2; contains exon(s) unassigned local exon(s) of the five-exon locus. The complete coding sequence is assembled across ctg000001c and ctg000003c. | partial genomic component of trans-spliced nad2; contains exon(s) 1,2 of the five-exon locus. The complete coding sequence is assembled across ctg000001c and ctg000003c.
ctg000001c.sdh3.86	No	No	No	No	No	—
ctg000001c.trnS-UGA.124	No	No	No	No	No	mitochondrion-native

Record ID	Duplicate	Focus duplicate	Partial	Pseudo-like	Trans-spliced	Source annotation note
ctg000001c.trnM-CAU.116	Yes	No	No	No	No	plastid-derived
ctg000001c.trnD-GUC.96	No	No	No	No	No	plastid-derived
ctg000001c.atp6.16	No	No	No	No	No	manual curation: atp6 3' boundary extended to 134355 ǀ manual curation: 3' boundary extended to the first in-frame TAG stop codon
ctg000001c.rpl5.132	No	No	Yes	Yes	No	manual curation: rpl5-like pseudogene fragment; only 38 aa of a same-genus Rpl5 homolog are retained and the genomic segment contains internal stop codons
ctg000001c.rps19.70	No	No	Yes	Yes	No	manual curation: rps19-like partial/pseudogene fragment; not counted as functional PCG ǀ manual curation: rps19-like partial/pseudogene fragment; not counted as a functional protein-coding gene
ctg000001c.rps3.73	Yes	Yes	No	No	No	—
ctg000001c.rpl16.64	Yes	Yes	No	No	No	start codon is not determined
ctg000001c.trnM-CAU.94	Yes	No	No	No	No	unknown tRNA sources
ctg000001c.trnG-GCC.102	Yes	No	No	No	No	mitochondrion-native
ctg000001c.trnQ-UUG.122	Yes	No	No	No	No	mitochondrion-native
ctg000001c.trnfM-CAU.114	Yes	No	No	No	No	mitochondrion-native
ctg000001c.trnW-CCA.126	No	No	No	No	No	plastid-derived
ctg000001c.nad9.55	No	No	No	No	No	—
ctg000001c.trnK-UUU.106	No	No	No	No	No	mitochondrion-native
ctg000001c.trnI-CAU.108	No	No	No	No	No	mitochondrion-native
ctg000001c.rps4.81	No	No	No	No	No	start codon of gene rps4 is probably ACG
ctg000001c.nad6.52	No	No	No	No	No	—
ctg000001c.trnA-UGC.128	No	No	No	No	No	manual curation: corrected from the PMGA split feature 349889-349925_join_350733-350768 to the continuous 349889-349973 tRNA-Ala locus; exact same-genus BLASTN support. tRNAscan-SE independently detected the locus, but its low-score intron model was not retained ǀ plastid-derived
ctg000001c.ccmFC.28	No	No	No	No	No	stop codon is created by C to U RNA editing
ctg000001c.ccmC.25	No	No	No	No	No	—
ctg000001c.atp4.13	No	No	No	No	No	—
ctg000001c.nad4L.49	No	No	No	No	No	start codon is not determined
ctg000001c.nad1.1	No	No	Yes	No	Yes	partial genomic component of trans-spliced nad1; contains exon(s) unassigned local exon(s) of the five-exon locus. The complete coding sequence is assembled across ctg000001c and ctg000003c. ǀ partial genomic component of trans-spliced nad1; contains exon(s) 4,5 of the five-exon locus. The complete coding sequence is assembled across ctg000001c and ctg000003c.
ctg000001c.matR.38	No	No	No	No	No	—
ctg000001c.mttB.41	No	No	No	No	No	start codon is not determined
ctg000001c.rps1.130	No	No	No	No	No	manual curation: complete rps1 ORF supported by a full-length same-genus TBLASTN match to Rhododendron simsii
ctg000001c.trnM-CAU.110	Yes	No	No	No	No	plastid-derived
ctg000001c.atp8.19	No	No	No	No	No	—
ctg000001c.cox3.35	No	No	No	No	No	—
ctg000001c.sdh4.89	No	No	No	No	No	—
ctg000001c.ccmB.22	No	No	No	No	No	—
ctg000001c.rpl10.58	No	No	No	No	No	—
ctg000001c.rrn26.84	No	No	No	No	No	—
ctg000001c.trnfM-CAU.112	Yes	No	No	No	No	mitochondrion-native
ctg000001c.nad4.44	No	No	No	No	No	—
ctg000001c.trnH-GUG.104	No	No	No	No	No	plastid-derived
ctg000001c.trnE-UUC.98	No	No	No	No	No	mitochondrion-native
ctg000001c.nad5.9	No	No	Yes	No	Yes	partial genomic component of trans-spliced nad5; contains exon(s) unassigned local exon(s) of the five-exon locus. The complete coding sequence is assembled across ctg000001c and ctg000003c. ǀ partial genomic component of trans-spliced nad5; contains exon(s) 4,5 of the five-exon locus. The complete coding sequence is assembled across ctg000001c and ctg000003c.
ctg000001c.ccmFN.32	No	No	No	No	No	—
ctg000002l.cob.1	No	No	No	No	No	—

Record ID	Duplicate	Focus cate	dupli-Partial	Pseudo-like	Trans-spliced	Source annotation note
ctg000002l.cox1.4	No	No	No	No	No	—
ctg000003c.rrn18.41	No	No	No	No	No	—
ctg000003c.rrn5.43	No	No	No	No	No	—
ctg000003c.nad7.31	No	No	No	No	No	—
ctg000003c.trnN-GUU.48	Yes	No	No	No	No	unknown tRNA sources
ctg000003c.nad1.1	No	No	Yes	No	Yes	partial genomic component of trans-spliced nad1; contains exon(s) 1 of the five-exon locus. The complete coding sequence is assembled across ctg000001c and ctg000003c. | partial genomic component of trans-spliced nad1; contains exon(s) 1,2,3 of the five-exon locus. The complete coding sequence is assembled across ctg000001c and ctg000003c.
ctg000003c.atp9.19	No	No	Yes	Yes	No	manual curation: partial atp9 fragment; not counted as functional atp9 copy | manual curation: partial atp9 fragment; not counted as a functional atp9 copy
ctg000003c.trnP-UGG.58	No	No	No	No	No	mitochondrion-native
ctg000003c.trnF-GAA.54	No	No	No	No	No	mitochondrion-native
ctg000003c.trnS-GCU.60	No	No	No	No	No	mitochondrion-native
ctg000003c.nad3.28	No	No	No	No	No	—
ctg000003c.rps12.38	No	No	No	No	No	—
ctg000003c.cox2.25	No	No	No	No	No	—
ctg000003c.nad2.6	No	No	Yes	No	Yes	partial genomic component of trans-spliced nad2; contains exon(s) unassigned local exon(s) of the five-exon locus. The complete coding sequence is assembled across ctg000001c and ctg000003c. | partial genomic component of trans-spliced nad2; contains exon(s) 3,4,5 of the five-exon locus. The complete coding sequence is assembled across ctg000001c and ctg000003c.
ctg000003c.trnY-GUA.62	No	No	No	No	No	mitochondrion-native
ctg000003c.trnN-GUU.56	Yes	No	No	No	No	plastid-derived
ctg000003c.trnC-GCA.52	No	No	No	No	No	mitochondrion-native
ctg000003c.trnR-UCG.50	No	No	No	No	No	unknown tRNA sources
ctg000003c.atp1.16	No	No	No	No	No	—
ctg000003c.sdh4.45	No	No	Yes	Yes	No	manual curation: partial sdh4 fragment; not counted as functional sdh4 copy | manual curation: partial sdh4 fragment; not counted as a functional sdh4 copy
ctg000003c.atp9.22	No	No	No	No	No	stop codon is created by C to U RNA editing
ctg000003c.nad5.11	No	No	Yes	No	Yes	partial genomic component of trans-spliced nad5; contains exon(s) unassigned local exon(s) of the five-exon locus. The complete coding sequence is assembled across ctg000001c and ctg000003c. | partial genomic component of trans-spliced nad5; contains exon(s) 1,2,3 of the five-exon locus. The complete coding sequence is assembled across ctg000001c and ctg000003c.

Panel D. Per-exon genomic coordinates (n = 91)

Gene	Category	Copy ID	Path	Record type	Locus exon	Local exon	Start	End	Strand	Length	Annotation note
atp1	ATP synthase	copy_1	ctg000003c	CDS	1	1	188073	189602	+	1530	—
atp4	ATP synthase	copy_1	ctg000001c	CDS	1	1	364539	365117	-	579	—
atp6	ATP synthase	copy_1	ctg000001c	CDS	1	1	133480	134355	+	876	manual curation: atp6 3' boundary extended to 134355 | manual curation: 3' boundary extended to the first in-frame TAG stop codon
atp8	ATP synthase	copy_1	ctg000001c	CDS	1	1	439995	440474	+	480	—
atp9	ATP synthase	copy_1	ctg000003c	pseudogene-like/partial fragment	1	1	102025	102228	-	204	manual curation: partial atp9 fragment; not counted as functional atp9 copy | manual curation: partial atp9 fragment; not counted as a functional atp9 copy
atp9	ATP synthase	copy_2	ctg000003c	CDS	1	1	197939	198163	+	225	stop codon is created by C to U RNA editing
ccmB	Cytochrome c bio-genesis	copy_1	ctg000001c	CDS	1	1	456758	457378	+	621	—
ccmC	Cytochrome c bio-genesis	copy_1	ctg000001c	CDS	1	1	361921	362754	-	834	—

Gene	Category	Copy ID	Path	Record type	Locus exon	Local exon	Start	End	Strand	Length	Annotation note
ccmFC	Cytochrome c biogenesis	copy_1	ctg000001c	CDS	1	1	353604	354376	+	773	stop codon is created by C to U RNA editing
ccmFC	Cytochrome c biogenesis	copy_1	ctg000001c	CDS	2	2	355329	355878	+	550	stop codon is created by C to U RNA editing
ccmFN	Cytochrome c biogenesis	copy_1	ctg000001c	CDS	1	1	538530	540269	+	1740	—
cob	Cytochrome b	copy_1	ctg000002l	CDS	1	1	16102	17283	+	1182	—
cox1	Cytochrome c oxidase	copy_1	ctg000002l	CDS	1	1	27325	28908	-	1584	—
cox2	Cytochrome c oxidase	copy_1	ctg000003c	CDS	1	1	111286	112083	+	798	—
cox3	Cytochrome c oxidase	copy_1	ctg000001c	CDS	1	1	441104	441901	+	798	—
matR	Maturase	copy_1	ctg000001c	CDS	1	1	398331	400298	-	1968	—
mttB	Transport membrane protein	copy_1	ctg000001c	CDS	1	1	408625	409413	+	789	start codon is not determined
nad1	NADH dehydrogenase	component_ctg000003c	ctg000003c	trans-spliced genomic component	1	—	100317	100701	-	385	trans-spliced genomic component
nad1	NADH dehydrogenase	component_ctg000003c	ctg000003c	trans-spliced genomic component	2	—	157497	157579	-	83	trans-spliced genomic component
nad1	NADH dehydrogenase	component_ctg000003c	ctg000003c	trans-spliced genomic component	3	—	156071	156262	-	192	trans-spliced genomic component
nad1	NADH dehydrogenase	component_ctg000001c	ctg000001c	trans-spliced genomic component	4	—	400961	401019	-	59	trans-spliced genomic component
nad1	NADH dehydrogenase	component_ctg000001c	ctg000001c	trans-spliced genomic component	5	—	397196	397454	-	259	trans-spliced genomic component
nad2	NADH dehydrogenase	component_ctg000001c	ctg000001c	trans-spliced genomic component	1	—	112133	112285	-	153	trans-spliced genomic component
nad2	NADH dehydrogenase	component_ctg000001c	ctg000001c	trans-spliced genomic component	2	—	110535	110926	-	392	trans-spliced genomic component
nad2	NADH dehydrogenase	component_ctg000003c	ctg000003c	trans-spliced genomic component	3	—	127621	127781	-	161	trans-spliced genomic component
nad2	NADH dehydrogenase	component_ctg000003c	ctg000003c	trans-spliced genomic component	4	—	125734	126306	-	573	trans-spliced genomic component
nad2	NADH dehydrogenase	component_ctg000003c	ctg000003c	trans-spliced genomic component	5	—	124044	124231	-	188	trans-spliced genomic component
nad3	NADH dehydrogenase	copy_1	ctg000003c	CDS	1	1	110206	110562	+	357	—
nad4	NADH dehydrogenase	copy_1	ctg000001c	CDS	1	1	501886	502346	-	461	—
nad4	NADH dehydrogenase	copy_1	ctg000001c	CDS	2	2	499526	500463	-	938	—
nad4	NADH dehydrogenase	copy_1	ctg000001c	CDS	3	3	496868	496956	-	89	—
nad4L	NADH dehydrogenase	copy_1	ctg000001c	CDS	1	1	365303	365605	-	303	start codon is not determined
nad5	NADH dehydrogenase	component_ctg000003c	ctg000003c	trans-spliced genomic component	1	—	198490	198719	+	230	trans-spliced genomic component
nad5	NADH dehydrogenase	component_ctg000003c	ctg000003c	trans-spliced genomic component	2	—	199556	200771	+	1216	trans-spliced genomic component

Gene	Category	Copy ID	Path	Record type	Locus exon	Local exon	Start	End	Strand	Length	Annotation note
nad5	NADH dehydrogenase	component_ctg000003c	ctg000003c	trans-spliced genomic component	3	—	62719	62740	-	22	trans-spliced genomic component
nad5	NADH dehydrogenase	component_ctg000001c	ctg000001c	trans-spliced genomic component	4	—	534854	535248	+	395	trans-spliced genomic component
nad5	NADH dehydrogenase	component_ctg000001c	ctg000001c	trans-spliced genomic component	5	—	536354	536503	+	150	trans-spliced genomic component
nad6	NADH dehydrogenase	copy_1	ctg000001c	CDS	1	1	347289	347906	+	618	—
nad7	NADH dehydrogenase	copy_1	ctg000003c	CDS	1	1	39015	39157	-	143	—
nad7	NADH dehydrogenase	copy_1	ctg000003c	CDS	2	2	38124	38192	-	69	—
nad7	NADH dehydrogenase	copy_1	ctg000003c	CDS	3	3	36174	36640	-	467	—
nad7	NADH dehydrogenase	copy_1	ctg000003c	CDS	4	4	34876	35119	-	244	—
nad7	NADH dehydrogenase	copy_1	ctg000003c	CDS	5	5	32917	33178	-	262	—
nad9	NADH dehydrogenase	copy_1	ctg000001c	CDS	1	1	277261	277833	-	573	—
rpl10	Large ribosomal subunit	copy_1	ctg000001c	CDS	1	1	457658	458149	-	492	—
rpl16	Large ribosomal subunit	copy_1	ctg000001c	CDS	1	1	40080	40514	+	435	start codon is not determined
rpl16	Large ribosomal subunit	copy_2	ctg000001c	CDS	1	1	202491	202925	+	435	start codon is not determined
rpl5	Large ribosomal subunit	copy_1	ctg000001c	pseudogene-like/partial fragment	1	1	146378	146488	+	111	manual curation: rpl5-like pseudogene fragment; only 38 aa of a same-genus Rpl5 homolog are retained and the genomic segment contains internal stop codons
rps1	Small ribosomal subunit	copy_1	ctg000001c	CDS	1	1	428455	428781	+	327	manual curation: complete rps1 ORF supported by a full-length same-genus TBLASTN match to Rhododendron simsii
rps12	Small ribosomal subunit	copy_1	ctg000003c	CDS	1	1	110610	110987	+	378	—
rps19	Small ribosomal subunit	copy_1	ctg000001c	pseudogene-like/partial fragment	1	1	37015	37254	+	240	manual curation: rps19-like partial/pseudogene fragment; not counted as functional PCG | manual curation: rps19-like partial/pseudogene fragment; not counted as a functional protein-coding gene
rps19	Small ribosomal subunit	copy_2	ctg000001c	pseudogene-like/partial fragment	1	1	199426	199665	+	240	manual curation: rps19-like partial/pseudogene fragment; not counted as functional PCG | manual curation: rps19-like partial/pseudogene fragment; not counted as a functional protein-coding gene
rps3	Small ribosomal subunit	copy_1	ctg000001c	CDS	1	1	37261	37334	+	74	—
rps3	Small ribosomal subunit	copy_2	ctg000001c	CDS	1	1	199672	199745	+	74	—
rps3	Small ribosomal subunit	copy_1	ctg000001c	CDS	2	2	38491	40108	+	1618	—
rps3	Small ribosomal subunit	copy_2	ctg000001c	CDS	2	2	200902	202519	+	1618	—
rps4	Small ribosomal subunit	copy_1	ctg000001c	CDS	1	1	345580	346638	+	1059	start codon of gene rps4 is probably ACG
rrn18	Ribosomal RNA	copy_1	ctg000003c	rRNA	1	1	15083	16902	+	1820	—
rrn26	Ribosomal RNA	copy_1	ctg000001c	rRNA	1	1	478897	482231	+	3335	—
rrn5	Ribosomal RNA	copy_1	ctg000003c	rRNA	1	1	17052	17168	+	117	—

Gene	Category	Copy ID	Path	Record type	Locus exon	Local exon	Start	End	Strand	Length	Annotation note
sdh3	Succinate dehydrogenase	copy_1	ctg000001c	CDS	1	1	112893	113213	-	321	—
sdh4	Succinate dehydrogenase	copy_1	ctg000001c	CDS	1	1	441829	442227	+	399	—
sdh4	Succinate dehydrogenase	copy_2	ctg000003c	pseudogene-like/partial fragment	1	1	190401	190541	+	141	manual curation: partial sdh4 fragment; not counted as functional sdh4 copy | manual curation: partial sdh4 fragment; not counted as a functional sdh4 copy
trnA-UGC	Transfer RNA	copy_1	ctg000001c	tRNA	1	1	349889	349973	+	85	manual curation: corrected from the PMGA split feature 349889-349925_join_350733-350768 to the continuous 349889-349973 tRNA-Ala locus; exact same-genus BLASTN support. tRNAscan-SE independently detected the locus, but its low-score intron model was not retained | plastid-derived
trnC-GCA	Transfer RNA	copy_1	ctg000003c	tRNA	1	1	130546	130616	-	71	mitochondrion-native
trnD-GUC	Transfer RNA	copy_1	ctg000001c	tRNA	1	1	132451	132524	+	74	plastid-derived
trnE-UUC	Transfer RNA	copy_1	ctg000001c	tRNA	1	1	515807	515878	+	72	mitochondrion-native
trnF-GAA	Transfer RNA	copy_1	ctg000003c	tRNA	1	1	103524	103597	-	74	mitochondrion-native
trnG-GCC	Transfer RNA	copy_1	ctg000001c	tRNA	1	1	53806	53877	+	72	mitochondrion-native
trnG-GCC	Transfer RNA	copy_2	ctg000001c	tRNA	1	1	216217	216288	+	72	mitochondrion-native
trnH-GUG	Transfer RNA	copy_1	ctg000001c	tRNA	1	1	513328	513401	-	74	plastid-derived
trnI-CAU	Transfer RNA	copy_1	ctg000001c	tRNA	1	1	297180	297256	+	77	mitochondrion-native
trnK-UUU	Transfer RNA	copy_1	ctg000001c	tRNA	1	1	296870	296942	+	73	mitochondrion-native
trnM-CAU	Transfer RNA	copy_1	ctg000001c	tRNA	1	1	40805	40874	+	70	unknown tRNA sources
trnM-CAU	Transfer RNA	copy_2	ctg000001c	tRNA	1	1	132228	132301	-	74	plastid-derived
trnM-CAU	Transfer RNA	copy_3	ctg000001c	tRNA	1	1	203216	203285	+	70	unknown tRNA sources
trnM-CAU	Transfer RNA	copy_4	ctg000001c	tRNA	1	1	437245	437317	+	73	plastid-derived
trnN-GUU	Transfer RNA	copy_1	ctg000003c	tRNA	1	1	65690	65760	-	71	unknown tRNA sources
trnN-GUU	Transfer RNA	copy_2	ctg000003c	tRNA	1	1	129409	129480	-	72	plastid-derived
trnP-UGG	Transfer RNA	copy_1	ctg000003c	tRNA	1	1	103193	103267	-	75	mitochondrion-native
trnQ-UUG	Transfer RNA	copy_1	ctg000001c	tRNA	1	1	56089	56160	+	72	mitochondrion-native
trnQ-UUG	Transfer RNA	copy_2	ctg000001c	tRNA	1	1	218500	218571	+	72	mitochondrion-native
trnR-UCG	Transfer RNA	copy_1	ctg000003c	tRNA	1	1	137210	137243	+	34	unknown tRNA sources
trnR-UCG	Transfer RNA	copy_1	ctg000003c	tRNA	2	2	138265	138324	+	60	unknown tRNA sources
trnS-GCU	Transfer RNA	copy_1	ctg000003c	tRNA	1	1	103946	104033	-	88	mitochondrion-native
trnS-UGA	Transfer RNA	copy_1	ctg000001c	tRNA	1	1	119141	119227	+	87	mitochondrion-native
trnW-CCA	Transfer RNA	copy_1	ctg000001c	tRNA	1	1	276501	276574	-	74	plastid-derived
trnY-GUA	Transfer RNA	copy_1	ctg000003c	tRNA	1	1	128288	128370	-	83	mitochondrion-native
trnfM-CAU	Transfer RNA	copy_1	ctg000001c	tRNA	1	1	82397	82470	-	74	mitochondrion-native
trnfM-CAU	Transfer RNA	copy_2	ctg000001c	tRNA	1	1	244808	244881	-	74	mitochondrion-native
trnfM-CAU	Transfer RNA	copy_3	ctg000001c	tRNA	1	1	482611	482684	+	74	mitochondrion-native

Notes: Panels A–C are linked by Record ID; coordinates are 1-based inclusive. Paths: ctg000001c = ctg1, ctg000002l = ctg2, and ctg000003c = ctg3. Totals: 32 functional PCG types (34 copy loci), 3 rRNA types (3 loci), 19 tRNA types (27 loci), and 5 partial/pseudogene-like fragments. The genes *rpl16* and *rps3* have two functional copies each. *nad1*, *nad2*, and *nad5* are trans-spliced; their component features are not independent complete genes. *Not available = not established by the original annotation; no missing CDS or codon values were inferred. Source: curated mitochondrial GenBank/GFF3 and trans-spliced exon annotations.
